# Novel Click Coupling Chemistry to Explore Glycan Recognition

**DOI:** 10.1021/acscentsci.4c02124

**Published:** 2025-04-23

**Authors:** Tianwei Jia, Akul Y. Mehta, Catherine A. Tilton, Ea Kristine Clarisse Tulin, Lauren E. Pepi, Lukas Muerner, Stephan von Gunten, Jamie Heimburg-Molinaro, Sean R. Stowell, Richard D. Cummings

**Affiliations:** † Department of Surgery, 1859 Beth Israel Deaconess Medical Center, Harvard Medical School, National Center for Functional Glycomics, CLS 11087-3 Blackfan Circle, Boston, Massachusetts 02115, United States; ‡ Institute of Pharmacology, 27210 University of Bern, Inselspital, INO-F, Bern 3010, Switzerland; § Joint Program in Transfusion Medicine, 1861 Brigham and Women’s Hospital, Harvard Medical School, 630E New Research Building, 77 Avenue Louis Pasteur, Boston, Massachusetts 02115, United States

## Abstract

Specific recognition of glycans by proteins is important in many biological processes and immune responses. Here we present a general approach for derivatizing free glycans with a novel linker MTZ (3-(methoxyamino)­propylamine added to a bioorthogonal-functional tetrazine tag) that exploits click chemistry to generate multiple platforms of glycan coupling. This derivatization preserves glycan integrity, is reversible and quantifiable, and incorporates a bioorthogonal tetrazine tag for click coupling. A library of ABO­(H) blood group MTZ-glycans was efficiently conjugated to avidin Luminex beads through a Biotin-PEG11-TCO (*trans*-cyclooctene) spacer, generating a multiplex array that was reproducibly interrogated in a high-throughput Luminex approach with multiple lectins and antibodies. We also rapidly profiled antiglycan IgG, IgM, and IgA antibodies in multiple, serially diluted human serum samples, revealing unique repertoires of antiglycan responses in each sera. Glycans were efficiently coupled to bovine serum albumin (BSA) at a high density (∼19–24 glycans/BSA) to generate a neoglycoprotein library that was useful in microarray formats that provided results equivalent to those obtained from the Luminex approach. Neoglycoproteins have many uses, including serving as acceptors for glycosyltransferases, as we demonstrate for assays of ST6Gal1 sialyltransferase. These facile and efficient technologies significantly expand the toolbox available to explore glycan–GBP interactions.

## Graphical abstract

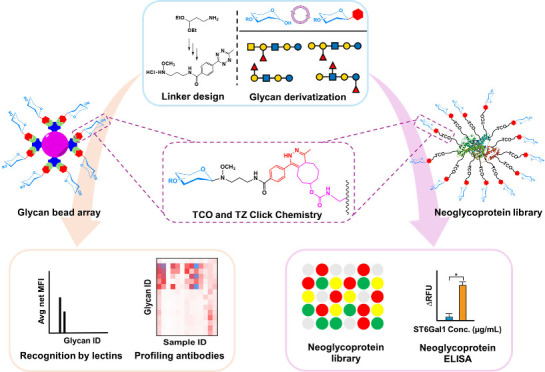

## Introduction

Glycans on glycoproteins, glycolipids, and proteoglycans are expressed by all organisms.^1,2^ While glycans have multiple functions, their primary functions often arise through their recognition by glycan-binding proteins (GBPs), which mediate numerous biological recognition processes including cell trafficking, adhesion, migration, and fertilization.^3–7^ Glycans also have roles in pathological processes where they are also recognized, and these include pathogen infection, cancer, inflammation, and autoimmune diseases.^8^ Changes in glycosylation patterns and roles in tumorigenesis and inflammation have also been observed.^9,10^ In addition, glycans serve as antigens in the ABO­(H) blood groups system, where they are expressed on the surfaces of erythrocytes and endothelial cells, and are crucial determinants of patient outcome in blood transfusion and organ transplantation.^11–13^ The presence of anti-ABO­(H) antibodies in recipients may lead to hyperacute antibody-mediated rejection initiated by ABO­(H) antigens in ABO-incompatible (ABOi) transplantation.^14,15^ Low titers of antibodies to blood group antigens, isohemagglutinins, are a characteristic and diagnostic feature of certain primary antibody deficiencies (PADs) such as common variable immunodeficiency (CVID).^16^ Individuals vary tremendously, however, in their expression of antiglycan antibodies, even those against the ABO­(H) antigens.^17,18^ Therefore, the suitability of donor cells and organs relies on the precise detection and quantification of specific anti-ABO antibodies.^19–21^ Because of these properties, studies on glycan function and their recognition by GBPs and antibodies have inspired the development of multiple therapeutic and diagnostic tools.^22–24^

Unlike the rapid advancements in nucleic acid and protein research, however, progress in understanding glycan structures and functional recognition has been slow, due to the heterogeneous nature of glycan structures and variability in post-translational modifications.^5^ To overcome the limitation of access to glycans, numerous efforts have been made to generate pure complex glycans through chemical and/or chemoenzymatic synthesis approaches.^25^ Yet, synthesis remains difficult due to the extensive manipulation of protecting groups needed and the limitation of selective glycosylation steps.^26^ In addition, the lack of available glycosyltransferases and their strict substrate and acceptor specificities limit the broad chemoenzymatic synthesis of complex glycans.^27^ On the other hand, glycans from natural sources such as tissues, bacteria, plants, and human milk oligosaccharides (HMOs) provide materials to partly address these limitations.^28–33^ However, natural sources of glycans and synthetic glycans require derivatization with proper functional tags at their anomeric positions to enable their isolation, purification, detection and quantification, and subsequent studies of their interactions with antibodies and GBPs in multiple formats.

In this regard multiple advancements have been made in bioconjugation,^34^ bioorthogonal chemistries,^35,36^ and the development of linkers or tags for glycan derivatization.^26,37–42^ Although various methods have been developed for glycan derivatization and subsequent microarray applications, there is a need for comprehensive methods that span the initial design of a multifunctional linker to its broad application across multiple analysis techniques, including multiplex bead assays, neoglycoprotein microarrays, and ELISA technologies. Here we present an approach that employs a novel linker and its derivatization to glycans to explore glycan–GBP interactions. This approach involves a multifunctional linker synthesis and glycan derivatization, which permits a high affinity, catalyst-free, and rapid coupling reaction through “click” chemistry, followed by the generation of a Luminex bead array and the fabrication of a neoglycoprotein library, which have various applications for investigating glycan–GBP interactions. We demonstrate that this method is general and ideal for various analytical technologies, including Luminex-based multiplexed glycan bead arrays, microarrays, and ELISAs, which facilitate the comprehensive profiling of GBP and antibody interactions with glycans.

## Results

### Design and Synthesis of MTZ Linker

Underivatized glycans, lacking intrinsic chromophores and possessing strong hydrophilicity, are difficult to separate and purify, which complicates their isolation and studies of their recognition and limits their use in various applications. To address such limitations, we developed a novel multifunctional linker, 3-(methoxyamino)­propylamine added to a bioorthogonal-functional tetrazine tag (MTZ linker), which was designed to have several key features. These include (1) the *N*-alkyl-O-methyl oxyamine conjugated with the hemiacetal of glycan reducing end under mildly acidic conditions resulting in the exclusive formation of the ring-closed product with high stereoselectivity for the β anomer.^26,40,43–45^ (2) From a broad perspective, a cleavable linker is an ideal tool for derivatizing glycans for biological studies and facilitating more detailed glycomics research after linker removal. The designed alkoxyamine-*N*-glycosides can be easily reversibly cleaved by *N*-chlorosuccinimide (NCS) to release the original hemiacetals.^46^ (3) The resulting MTZ linker contains an aromatic group, which enhances sensitivity for multidimensional chromatographic separation and facilitates the quantitation of glycan fractions before further applications. (4) The 1,2,4,5-tetrazine (TZ) functional group, which reacts with *trans*-cyclooctene (TCO) through an inverse electron demand Diels–Alder (iEDDA) “click” reaction with high affinity and a catalyst-free and fast reaction rate (∼1–10^6^ M^–1^ s^–1^),^47^ was introduced into the linker to enhance versatility for applications such as array printing, bead assays, and neoglycoprotein synthesis.

The multifunctional MTZ linker was synthesized from 3-aminopropionaldehyde diethyl acetal (Scheme 1). Briefly, the starting material was protected with an Fmoc group, followed by hydrolysis to yield aldehyde compound **2**, which was subsequently condensed with methoxyamine. The resulting imine was reduced by using NaBH_3_CN to afford compound **3**. Compound **4**, with its Fmoc group deprotected, was coupled with the carboxyl group of tetrazine to yield Boc-protected compound **5**. This was treated with 4 M HCl in dioxane to produce the MTZ hydrochloride salt linker in quantitative yield. The ^1^H NMR spectrum of MTZ showed resonances at 3.00 and 3.76 ppm, corresponding to the methyl and methoxy protons, respectively. In the ^13^C NMR spectrum, the corresponding carbon resonances for the methyl and methoxy groups were observed at 20.13 and 61.35 ppm, respectively. Detailed information on synthesis is provided in the Supporting Information.

**Scheme 1:**
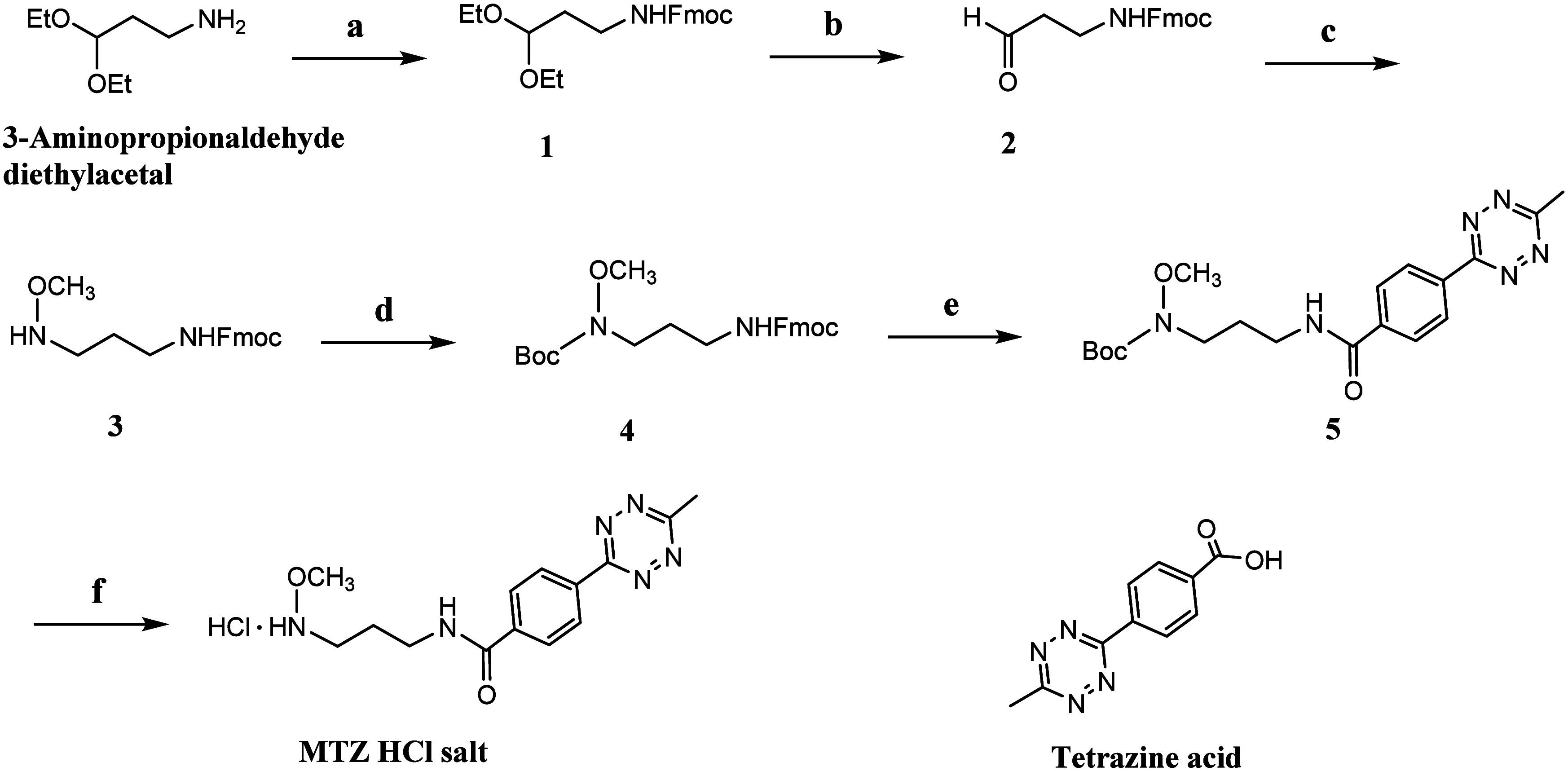
Reagents and Conditions: (a) Fmoc-Cl, DIPEA, DCM, rt, quant yield; (b) 4 M HCl in dioxane, DCM, rt, 75%; (c) (1) CH_3_ONH_2_ · HCl, DIPEA, DCM, rt, (2) NaBH_3_CN DCM/HOAc, 5/1, 0 °C, 81%; (d) (Boc)_2_O, DIPEA, DCM, rt, 79%; (e) (1) 20% piperidine in DMF (v/v), rt, 30 min, (2) Tetrazine acid, HATU, DIPEA, DMF, rt, 68%; (f) 4 M HCl in dioxane, DCM, rt, quant yield

### Derivatization of Free Oligosaccharides

With this versatile MTZ linker, we derivatized the prototypical glycans lacto-*N*-neotetraose (LNnT) and 3′-sialyllactose (3′-SL) using our previously reported reaction conditions with slight modifications.^26^ The free reducing glycans were mixed with 100 equiv of the MTZ linker and 25 equiv of 2-amino-5-methoxybenzoic acid (2-AM) in a DMSO/AcOH solution and shaken for 4 h at 65 °C. The resulting LNnT-MTZ and 3′-SL-MTZ were identified by MALDI-TOF mass spectrometry (Figure S1). LNnT-MTZ was used to investigate the hydrolysis of alkoxyamine-N-glycosides and the reversible release of the free glycan using NCS. LNnT-MTZ was treated with NCS in water for 2 h, resulting in the cleavage of the glycoside to yield the original LNnT, as confirmed by MALDI-TOF MS, with an over 85% yield. Furthermore, the UV–vis absorption spectra of LNnT-MTZ demonstrated a strong absorption peak at 270 nm in the UV region and a weaker absorption peak at 520 nm in the visible region. Additionally, fluorescence analysis revealed that both MTZ and LNnT-MTZ are equivalently fluorescent with an excitation at 520 nm and an emission at 580 nm (Figure S2).

To further explore the versatility of the linker, we generated a multiplex glycan bead array and neoglycoprotein preparations by derivatizing MTZ to 14 blood group glycans that represent various human blood group antigens (Figure 1). The purity of the blood group glycans was tested using ESI-LC-MS. The majority of the compounds were determined to be greater than 80% pure; however, a few did contain isomers to the expected structure (Figure S3). The MTZ-linked blood group glycans were purified by C-18 SPE and analyzed by MALDI-TOF mass spectrometry (Figure S4).

**Figure 1:**
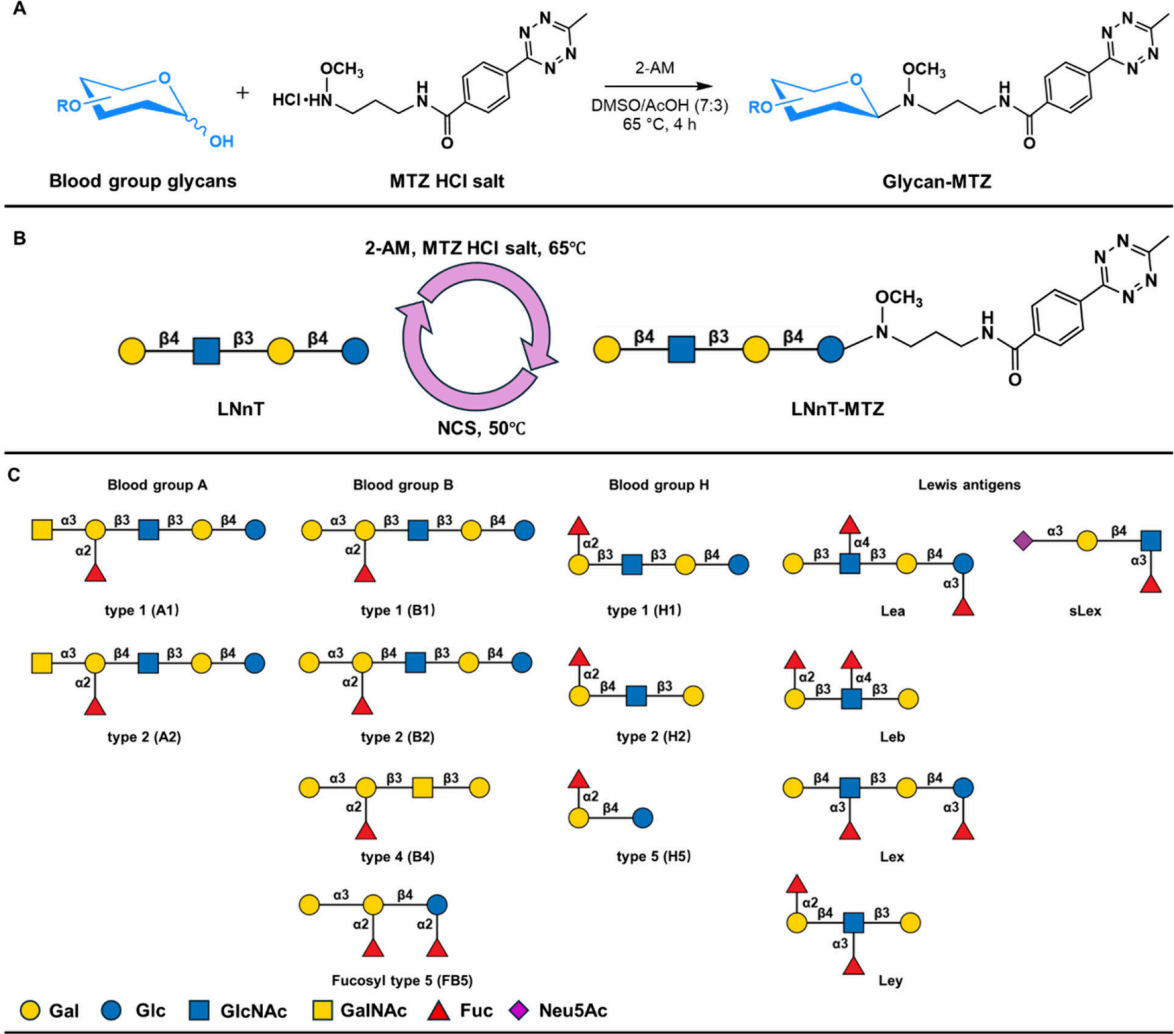
(A) Derivatization of blood group glycans with MTZ HCl salt. (B) Schematic of derivatization of LNnT and cleavage of the linker. (C) Structures of 14 commercial blood group glycans with their name/designation.

### Optimization and Conjugation of Glycan-MTZ with Luminex Beads

These glycans were used in a novel strategy to generate a multiplex glycan bead array, as depicted in Figure 2. Briefly, we exploited the derivatization of Luminex avidin beads with Biotin-PEG11-TCO, which provides Luminex-TCO beads. Various glycan-MTZ compounds were used to efficiently modify the TCO through a “click reaction”, forming glycan derivatized beads. To explore the optimal conditions of glycan conjugation to Luminex beads, a titration curve of Biotin-PEG11-TCO and glycan-MTZ was evaluated (Figure S5). To address the scalability of glycan bead preparation, an excess amount of Biotin-PEG11-TCO (0.5 μg per 100,000 Luminex beads) was used to ensure saturation of the Luminex beads, even though other concentrations yielded similar results (Figure S5A). Subsequently, glycan-MTZ in the amount of 1 μg per 100,000 TCO beads was required to achieve the maximum signal (Figure S5B). The successful generation of TCO-modified beads was confirmed using a Sulfo-Cy3-Methyltetrazine dye. As shown in Figure S6, TCO-modified beads exhibited orange fluorescence in the Cy3 channel after staining with Sulfo-Cy3-Methyltetrazine dye, whereas avidin beads showed no fluorescence in the Cy3 channel and retained their original fluorescence in the Cy5 channel. Under optimal conditions, the glycan beads were prepared, and the remaining TCO reaction sites were evaluated using Sulfo-Cy3-Methyltetrazine fluorescent dye. All glycan-coated beads retained their original magenta fluorescence in the Cy5 channel, and no fluorescence from the Sulfo-Cy3-Methyltetrazine dye was observed in the Cy3 channel, indicating that derivation was highly efficient and the TCO reactive sites were fully saturated with glycan-MTZ (Figure S7). These images confirmed that the bead regions were successfully modified with Biotin-PEG11-TCO.

**Figure 2:**
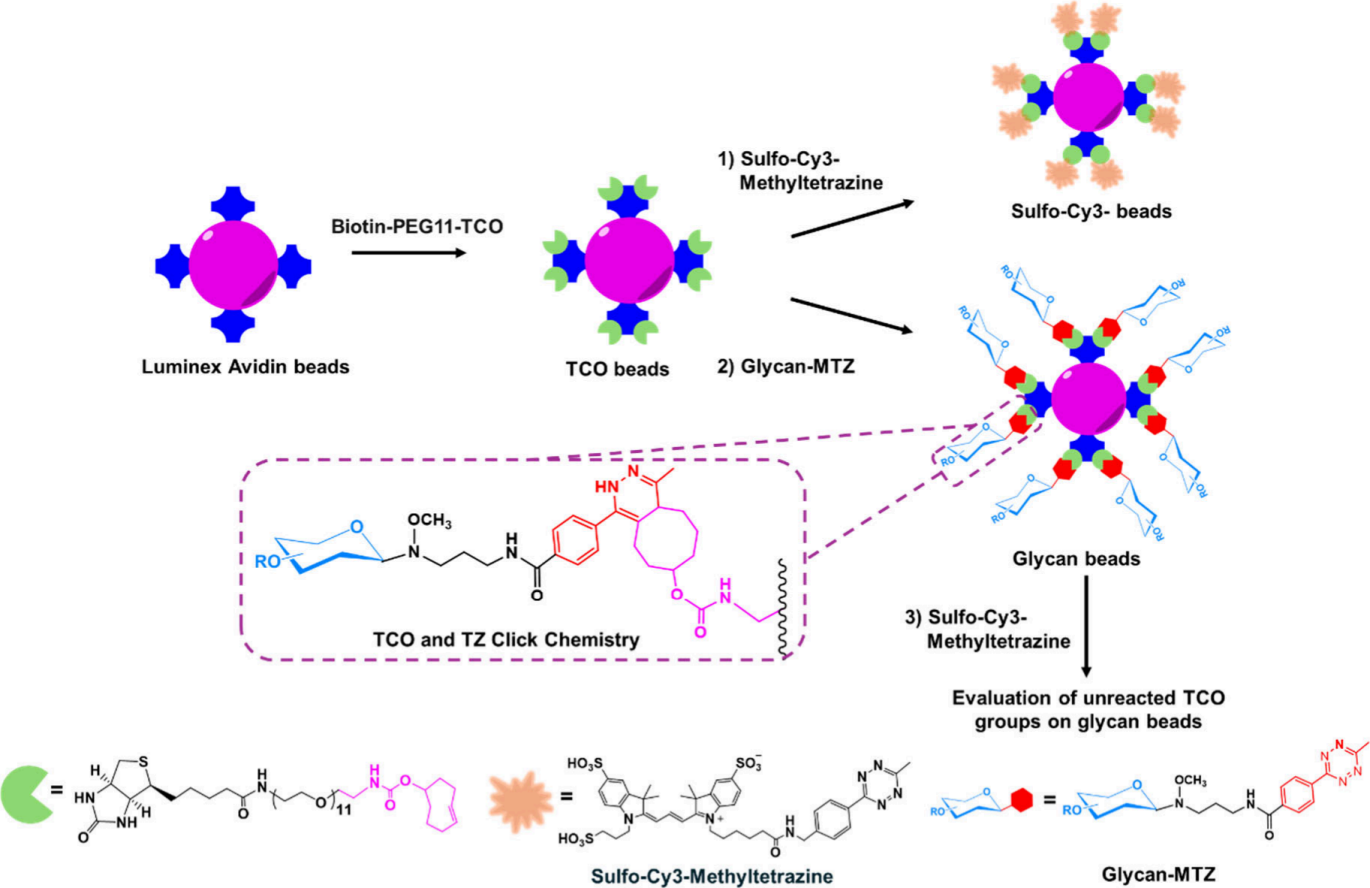
Schematic of multiplex glycan bead array preparation. Luminex Avidin beads of various bead regions were individually coated with Biotin-PEG11-TCO. (1) The TCO beads were stained with Sulfo-Cy3-Methyltetrazine to check the availability of TCO on beads (higher signal indicative of more reactive surface). (2) Beads from individual bead regions were coated with pure Glycan-MTZ derivatives. (3) Coated beads were stained with Sulfo-Cy3-Methyltetrazine to ensure that the TCO sites on the beads were occupied by the glycan (lower signal indicative of more thorough coating by the glycan).

### Recognition of Glycans by Plant Lectins

Luminex technology offers a multiplexed platform allowing the simultaneous detection of binding to multiple ligands within a single well in a microplate by mixing different glycan beads together. The multiplexed glycan bead array, containing 14 ABO­(H) blood group glycans, was evaluated using six well-characterized fluorescently labeled plant lectins containing APDye-532 to probe each of the glycan beads in the mix. As shown in Figure 3, each lectin binds to a specific glycan motif. *Aleuria aurantia* lectin (AAL) binds to fucose^48^ and recognizes a broad spectrum of α-linked fucose-containing glycans with discrimination between different glycan structures. AAL binds to blood group H glycans and Lewis antigens, which contain α-fucose in the α-2, α-3, and α-4 linkages. Neither blood group A glycans [GalNAcα1–3­(Fucα1–2)­Galβ1–3/4GlcNAc] nor blood group B glycans [Galα1–3­(Fucα1–2)­Galβ1–3/4GlcNAc/GalNAc] are recognized by AAL, except for the fucosyl B type 5 (FB5) glycan, which contains an extra fucose residue (Figure 3A). *Griffonia simplicifolia* lectin-I (GSL-I) has strong interactions with glycans containing terminal α-Gal residues.^49^ Consequently, blood group B glycans exhibit weak binding with GSL-I due to the inhibition caused by the proximal fucose residue (Figure 3B).^50^ *Dolichos biflorus* agglutinin (DBA)^51^ and *Helix pomatia* agglutinin (HPA)^52^ both bind to a variety of glycans with terminal α-GalNAc, including blood group A glycans [GalNAcα1–3­(Fucα1–2)­Gal]. Therefore, all of the blood group A glycans were recognized by DBA and HPA (Figure 3C,D). The weak binding of DBA to H5 glycan [Fucα1–2Galβ1–4Glc] was also observed. *Peanut* agglutinin (PNA) binds to glycans with the terminal Galβ1–3GalNAc motif.^53^ Since this glycan motif is not present in the glycan library, no binding signal was detected (Figure 3E). *Ulex europaeus* agglutinin-I (UEA-I) can bind to α1–2-linked fucose as in the terminal Fucα1–2Galβ1–4GlcNAc/Glc motif,^54^ as indicated in Figure 3F. Blood group H type 2 and type 5 [Fucα1–2Galβ1–4GlcNAc/Glc] were bound by UEA-I, unlike blood group H type 1 [Fucα1–2Galβ1–3GlcNAc], which includes a different Gal linkage. Moreover, Fucα1–3 on the GlcNAc residue as in the Le^y^ structure is also tolerated by UEA-I. Furthermore, we calculated the apparent *K*_d_ of interactions with various lectins to specific glycans using the Luminex glycan bead array. These studies revealed that AAL has high-affinity binding (*K*_d_ = 4.0 nM) to glycan H2, while lectin HPA shows similarly strong binding (*K*_d_ = 4.6 nM) to glycan A1, without significant binding to the control glycan (Figure S8). Taken together, the lectin evaluations demonstrated that all the glycans were well conjugated with Luminex beads and presented in a manner promoting high affinity interactions, confirming the successful preparation of the glycan bead array.

**Figure 3:**
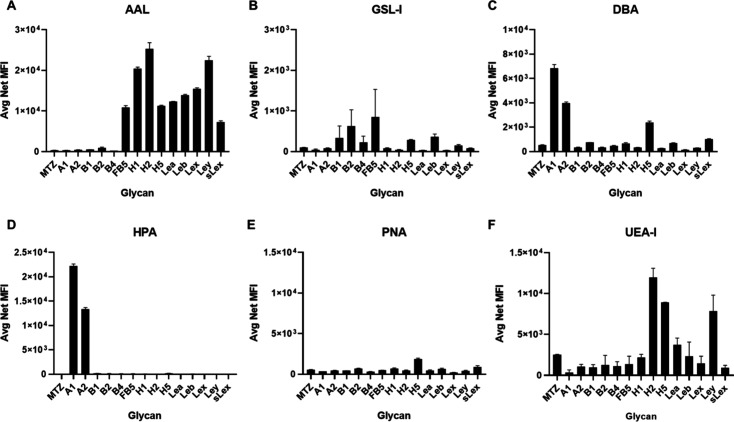
Binding of six lectins on the Luminex glycan bead array. Glycan names on *x*-axis correspond to Figure 1C. (A) AAL, (B) GSL-I, (C) DBA, (D) HPA, (E) PNA, and (F) UEA-I. The *y*-axis represents the average net (background subtracted) median fluorescence intensity (MFI). The data are presented as an average net MFI of two replicates; each experiment was repeated two times. Error bars: ±1 SD.

### Recognition of Glycans by Antiglycan Antibodies

We tested the interactions of these glycans within the Luminex glycan bead array with three different antiglycan monoclonal antibodies produced in our laboratory.^55^ As shown in Figure 4A, the antiglycan antibody (Tn4–31L) binds specifically to the H2 glycan [Fucα1–2Galβ1–4GlcNAcβ1–3Gal] but neither to glycan H1 [Fucα1–2Galβ1–3GlcNAcβ1–3Galβ1–4Glc] nor to glycan H5 [Fucα1–2Galβ1–4Glc], indicating that Tn4–31L effectively discriminates between different glycan linkages and sequences. The other antiglycan antibody (OmcFL3–02) exhibits high affinity binding to glycan H2 and weak binding to glycan H5. Glycan H1, which has a similar motif, was not bound by the OmcFL3–02 antibody (Figure 4B). In addition, neither Tn4–31L nor OmcFL3–02 recognized the Le^y^ glycan [Fucα1–2Galβ1–4­(Fucα1–3)­GlcNAcβ1–3Gal], which contains an additional substituent attached to GlcNAc that blocks antibody recognition but is recognized by lectin UEA-I. These data illustrate the importance of glycan structure in the interaction of glycans and antibodies and the lack of cross reactivity as a result of high-density conjugation on beads. As expected, no binding signal was observed with the antiglycan antibody PBMC3–02, as this antibody only recognizes glycans with terminal α2–6 sialic acid residues, which are not present in this ABO­(H) library (Figure 4C). These data demonstrate the ability of the general method of preparing the Luminex glycan beads to evaluate antibody–glycan interactions.

**Figure 4:**
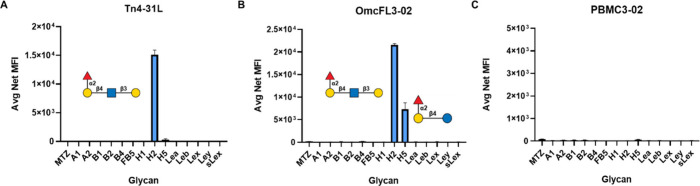
Recognition of antiglycan antibodies by Luminex glycan bead array. Antiglycan antibody: (A) Tn4–31L, (B) OmcFL3–02, and (C) PBMC3–02. Glycan names on *x*-axis correspond to Figure 1C. The *y*-axis represents the average net (background subtracted) median fluorescence intensity (MFI). Data are presented as an average net MFI of two replicates; each experiment was repeated two times. Error bars = ±1 SD.

### Profiling of Antiglycan lgG, IgM, and IgA Antibodies in Human Samples

We next profiled the specific antiglycan IgG, IgM, and IgA antibodies in 13 individual human sera, with each sample diluted serially from 1:100 to 1:6400. Among these 13 human samples tested, the blood types of only two donors were available: serum sample 6, which is blood group A, and serum sample 7 which is blood group O. An example of the binding data is provided in Figure 5A–C, which illustrates the evaluation of antiglycan IgG, IgM, and IgA levels, respectively, at three different dilutions of sample 2. The antibody repertoires for each individual and each glycan, based on the 1:200 dilution samples, are illustrated in Figure 5D–F. A detailed evaluation of all samples, serially diluted from 1:100 to 1:6400, is provided in the Supporting Information (Figure S9). We observed unusual reactivity with the 1:100 sample vs the 1:200 sample, and we speculate that this behavior may be attributed to either increased aggregation of IgM at higher concentrations and/or increased nonspecific binding in the no-glycan control at lower dilutions for certain serum samples.

**Figure 5:**
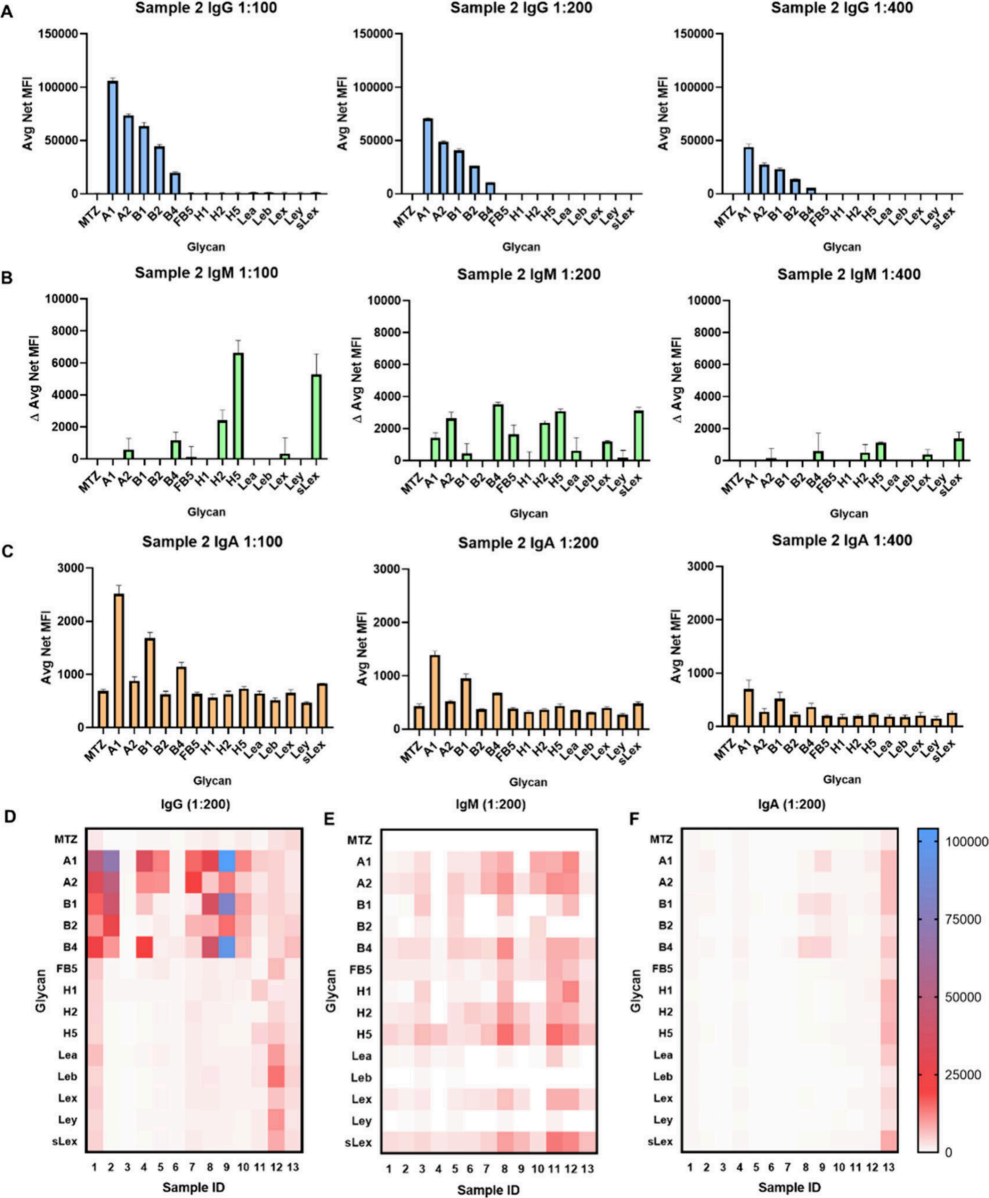
Example: Profiling of antiglycan antibodies, showcasing (A) IgG, (B) IgM, and (C) IgA, across three different dilutions of sample 2. Heat map of natural antiglycan IgG, IgM, and IgA in 13 samples. The levels represent the 1:200 dilution of samples, and the color codes are indicated in the key. Each color code represents the average net (background subtracted) median fluorescence intensity (MFI) in parts D and F. Each color code represents the average net MFI subtracted MTZ (no glycan control) in panel E. Glycan names on *x*-axis correspond to Figure 1C. The data are presented as an average net MFI of two replicates; each experiment was repeated two times. A detailed evaluation of each sample, serially diluted from 1:100 to 1:6400, is provided in the Supporting Information (Figure S9).

Although the repertoire of antiglycan antibodies in each individual tested is unique, some general trends could be observed. In most samples, antiglycan IgG showed the highest signals, and antiglycan IgA indicated the lowest signals in the same individual. In addition, even for antibodies recognizing a common motif, such as the antiblood group A or antiblood group B antibody, we observed a different pattern of binding to glycans between individuals. Furthermore, within the same individual, the same type of antibody (such as antiblood group A or B) has a different pattern of binding with glycans providing information about the specificity and diversity of the immune response. Comparing the different isotypes of antiglycan antibodies, antiglycan IgM antibodies recognized the greatest range of glycans, and most antiglycan IgG and IgA antibodies recognized blood group A and B glycans. Interestingly, antibody reactivities of antiglycan IgM antibodies against Lewis structures Le^x^ and sLe^x^ could be detected in nearly all samples. Humans commonly generate serum antibodies to microbial glycans, including blood group antigens,^13^ and recognition of Le^x^ and sLe^x^ might arise as it is known that the common microbe *Helicobacter pylori* can express Le^x^ and sLe^x^ in its glycans.^56^ Exceptionally, in the 1:200 dilution sample 3, no reactivity was detected for antiglycan IgG and IgA antibodies, and only reactivity was observed for antiglycan IgM antibodies. The strongest antiglycan reactivity was found in sample 9 of the IgG isotype, which showed the strongest binding to A1, B1, and B4 glycans. Low reactivity was observed for antiglycan IgM and IgA antibodies in sample 9. The reactivity of antiglycan IgM antibodies was higher than antiglycan IgG antibodies in sample 11, and all three antibody isotypes showed similar signal levels in sample 13. Additionally, antiglycan IgG antibodies recognized the Lewis structure glycans in sample 12. Of the 2 serum samples from donors with known blood types, their antiglycan repertoires were as predicted; antiglycan IgG antibodies in sample 6 (blood type A) only showed low binding to the B4 glycan, and antiglycan IgG antibodies in sample 7 (blood type O) showed binding to the A1, A2, B1, B2, and B4 glycan. These results indicated that the Luminex bead array has the potential to function in a high-throughput platform to identify tremendous variations in IgM, IgG, and IgA antibodies and their serum titers to different ABO­(H) and related antigens.

### General Preparation of Neoglycoproteins

Bovine serum albumin (BSA), a ∼66 kDa protein, is a commonly used carrier in the preparation of neoglycoproteins due to its affordability, lack of glycosylation, and the abundance of nucleophilic amino acid side chains, which facilitate chemical conjugation.^17,57–59^ BSA was preincubated with EDTA buffer (BBS-EDTA) to remove any metal impurities in BSA (e.g., copper) catalyzing *trans*-cyclooctene into inactive *cis*-cyclooctene.^60^ The strategy for the preparation is shown in Figure 6.

**Figure 6:**
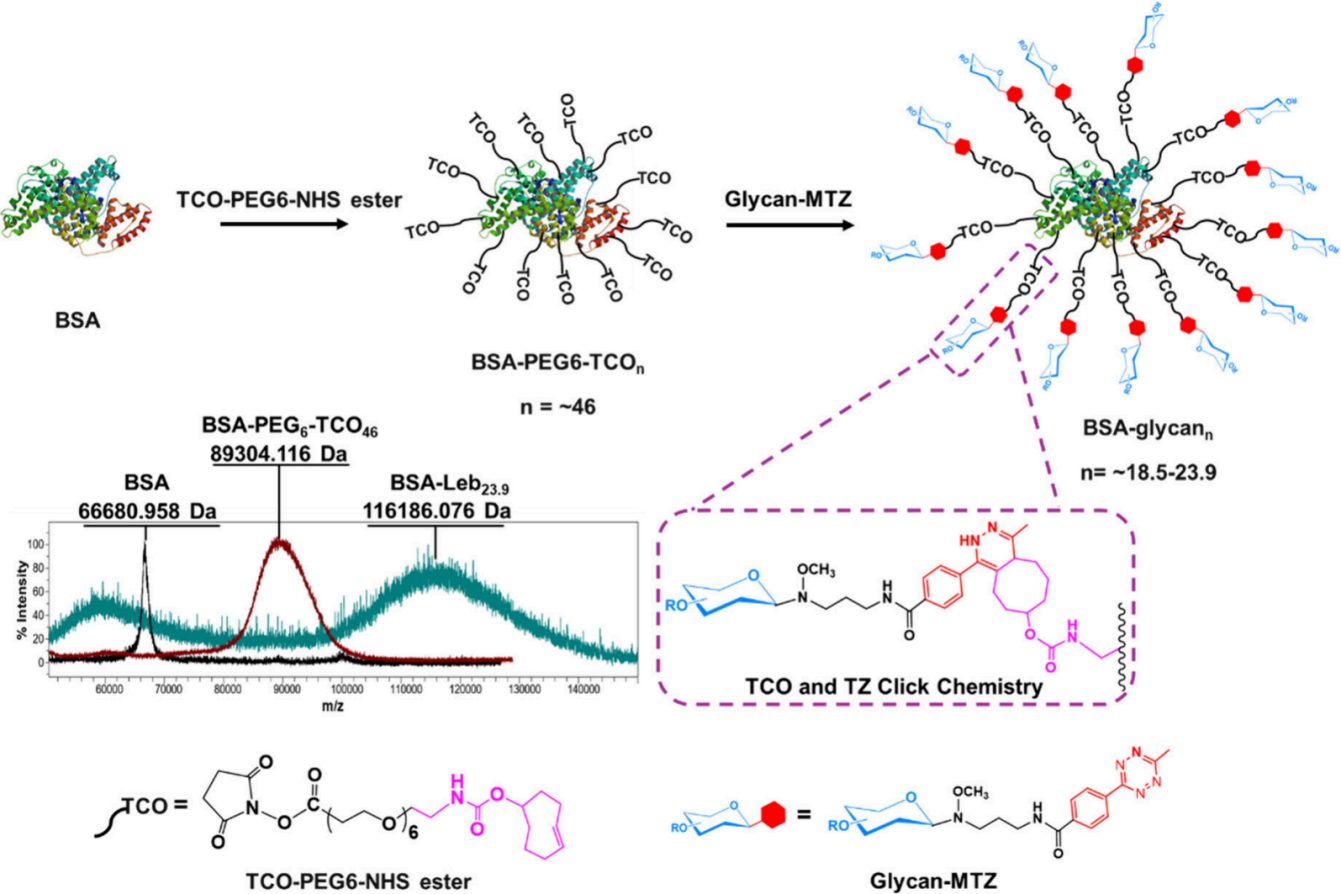
Schematic of neoglycoprotein library preparation.

First, to functionalize surface lysine residues of BSA, TCO-PEG6-*N*-hydoxy-succinimide (TCO-PEG6-NHS ester) linkers were introduced to react with the lysine amine groups. To maximize the degree of conjugation to BSA, various equivalents of TCO-PEG6-NHS ester (10–100 equiv) were incubated with BSA. The resulting BSA-PEG6-TCO conjugates were assessed by MALDI-TOF mass spectrometry (Table S1, Figure S10). As reported,^61^ approximately 20% of the total lysine residues in BSA (60 residues) are buried to varying extents within the protein interior. The optimized protocol with 100 equiv of TCO achieved a TCO:BSA ratio of 46:1, indicating approximately 80% of the lysine residues were modified with TCO-PEG6-NHS ester. Next, glycan 3′-SL-MTZ was utilized to investigate the conjugation between BSA-PEG6-TCO_46_ and glycan-MTZ. We incubated BSA-PEG6-TCO_46_ with 10–500 equiv of 3′-SL-MTZ and analyzed the resulting BSA-3′-SL by MALDI-TOF mass spectrometry (Table S1, Figure S11). The maximum degree of 3′-SL-MTZ conjugation was achieved in 100 equiv with the average number of 3′-SL:BSA ratio of 21:1. The finding that a higher concentration of 3′-SL did not increase the average number of glycans conjugated to BSA is likely due to the steric hindrance effect, where the reacted and bound 3′-SL interferes with access to the unreacted sites. Under optimal conditions, we prepared a neoglycoprotein library, with different glycans conjugated to BSA, resulting in a consistent average number of glycans ranging from 18.5 to 23.9 (Table 1, Figure S12).

**Table 1:** Summary of Average Number of Glycans Conjugated to BSA in the Neoglycoprotein Library

Glycan ID	Average Loading #	Glycan ID	Average Loading #	Glycan ID	Average Loading #
A1	23.7	FB5	22.3	Leb	23.9
A2	21.5	H1	21.9	Lex	22.8
B1	19.7	H2	22.7	Ley	19.6
B2	18.5	H5	20.6	sLex	18.8
B4	19.5	Lea	20.2		

### Microarray Prepared with the Neoglycoprotein Library

A key application of neoglycoproteins is their use in microarray formats to evaluate glycan-mediated recognition events.^17,57^ We separately printed the neoglycoproteins on nitrocellulose slides and epoxide coated slides for comparison. Nitrocellulose slides allow the immobilization of neoglycoproteins through noncovalent interactions, whereas neoglycoproteins rely on the amine or thiol functional group to react with the epoxide group to immobilize on the slide surface.^62^ Binding of AAL, which binds to fucose-containing glycans, was used to evaluate the neoglycoprotein microarray printed on both types of slides. As expected, AAL binds to fucosyl B type 5 (FB5) glycan, which contains an extra fucose unit, as well as blood group H glycans and Lewis antigens, which contain α-fucose in α-2, α-3, and α-4 linkages (Figure 7A,B). Interestingly, we observed no binding to A1 glycan, whereas there was significant binding of A2 and B2 glycan; only weak binding was observed to B1 and B4 with AAL, which generally shows a lack of binding to blood group A and B in the glycan microarray.^26,41^ A2 and B2 glycans have the Galβ1–4GlcNAc linkage, whereas A1, B1, and B4 glycans contain the Galβ1–3GlcNAc/GalNAc sequence (Figure 1). The results demonstrate that neoglycoproteins in microarray formats provide valuable linkage-specific information in recognition by AAL. Because approximately 80% of lysine residue amines in BSA are occupied by linkers, this results in fewer available reaction sites for the epoxide group on the epoxy slides. This may explain the roughly 2-fold lower signal we observed using the epoxy slides compared to the nitrocellulose slide. Next, the neoglycoprotein library printed on nitrocellulose slides was evaluated with plant lectins HPA and GSL-I. As shown in Figure 7C and D, blood group A glycans were bound by HPA, and blood group B glycans were bound by GSL-I. Furthermore, GSL-I showed weak binding to Le^b^ [Fucα1–2Galβ1–3­(Fucα1–4)­GlcNAcβ1–3Gal] and Le^y^ [Fucα1–2Galβ1–4­(Fucα1–3)­GlcNAcβ1–3Gal]. These results suggest this new approach for neoglycoprotein microarrays in which glycans are coupled at high densities and relatively similar degrees of conjugation can enhance studies to explore GBP–glycan interactions.

**Figure 7:**
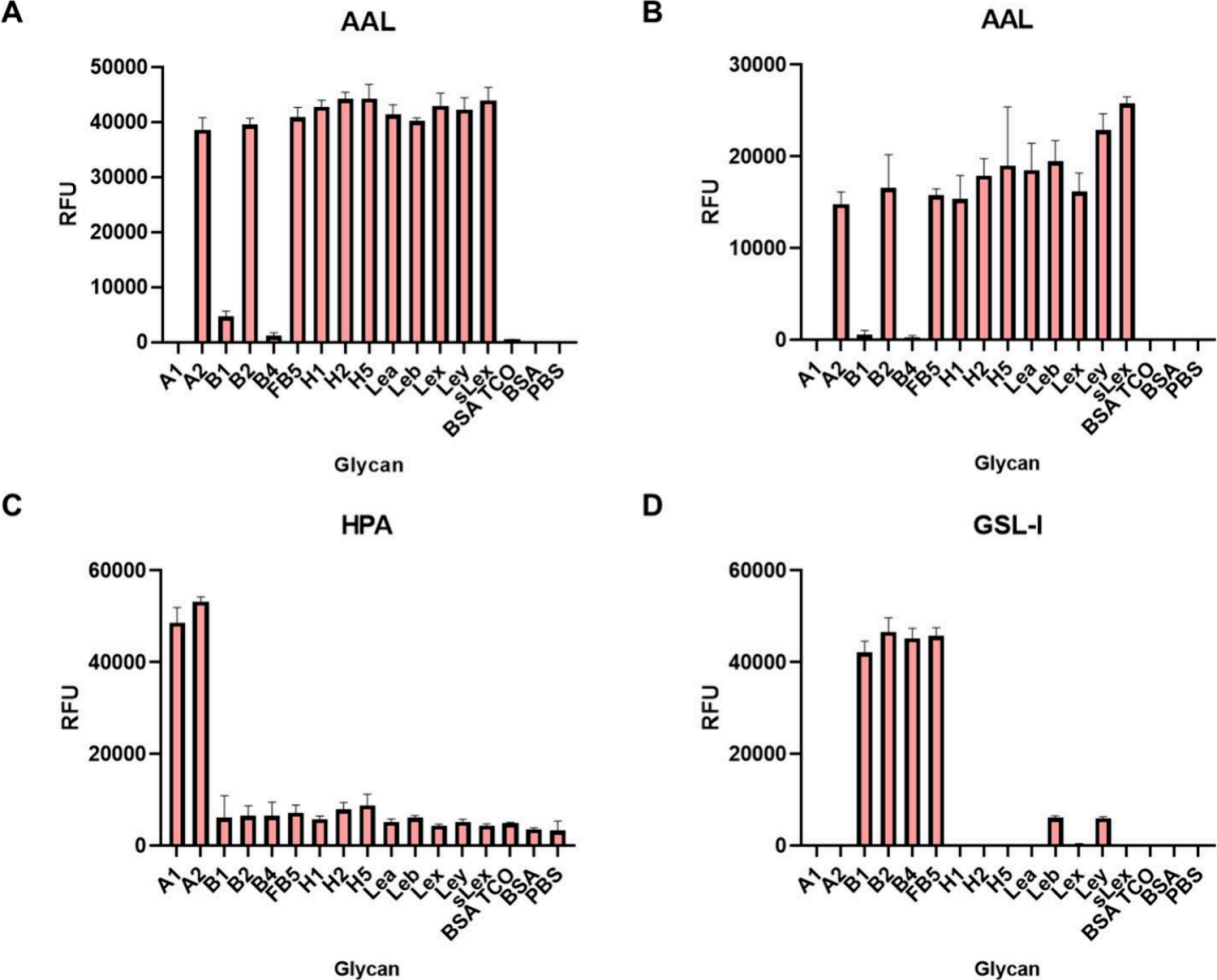
Recognition of three lectins (20 μg/mL) by neoglycoprotein microarrays. (A, C, and D) Printed on nitrocellulose slides. (B) Printed on an epoxide slide. Glycan names on *x*-axis correspond to Figure 1C. Error bar represents mean ± 1 SD from analyses of four independent experiments. RFU = relative fluorescence units.

### Neoglycoprotein ELISA

We further evaluated the performance of neoglycoproteins in ELISA assays to explore GBP binding as well as their potential use as acceptors to measure glycosyltransferase activities and specificities. The optimum immobilization of neoglycoprotein BSA-LNnT in a 96-well ELISA plate format was investigated by testing various concentrations of BSA-LNnT (0–500 ng/well). After immobilization, the neoglycoprotein BSA-LNnT was detected with fluorescein-labeled *Ricinus communis* agglutinin-I (RCA-I), which binds to glycans with terminal β-linked galactose. The binding signal reached its maximum at 125 ng/well, while 250 ng/well exhibited relatively lower variation (Figure S13). Consequently, BSA-LNnT at 250 ng/well was used in further assays to ensure reproducibility of the ELISA assay. All the BSA-LNT and BSA-LNnT used in the assay were characterized by MALDI-TOF mass spectrometry (Figure S14).

We next tested the feasibility of using this neoglycoprotein as an acceptor for the recombinant sialyltransferase activity, ST6Gal1. BSA-LNnT immobilized in a 96-well plate was incubated with ST6Gal1 and freshly prepared donor substrate CMP-Sia (Figure 8A). Detection of product formation utilized binding by fluorescence-labeled *Sambucus nigra* agglutinin (SNA), which binds α2,6-linked sialic acid,^63^ as well as the VLRB-based mAb PBMC3–02, which also binds α2,6-linked sialic acid. The ST6Gal1-treated samples demonstrated a significant increase in binding by both SNA and mAb PBMC3–02 compared to the nontreated group (Figure 9A,B).

**Figure 8:**
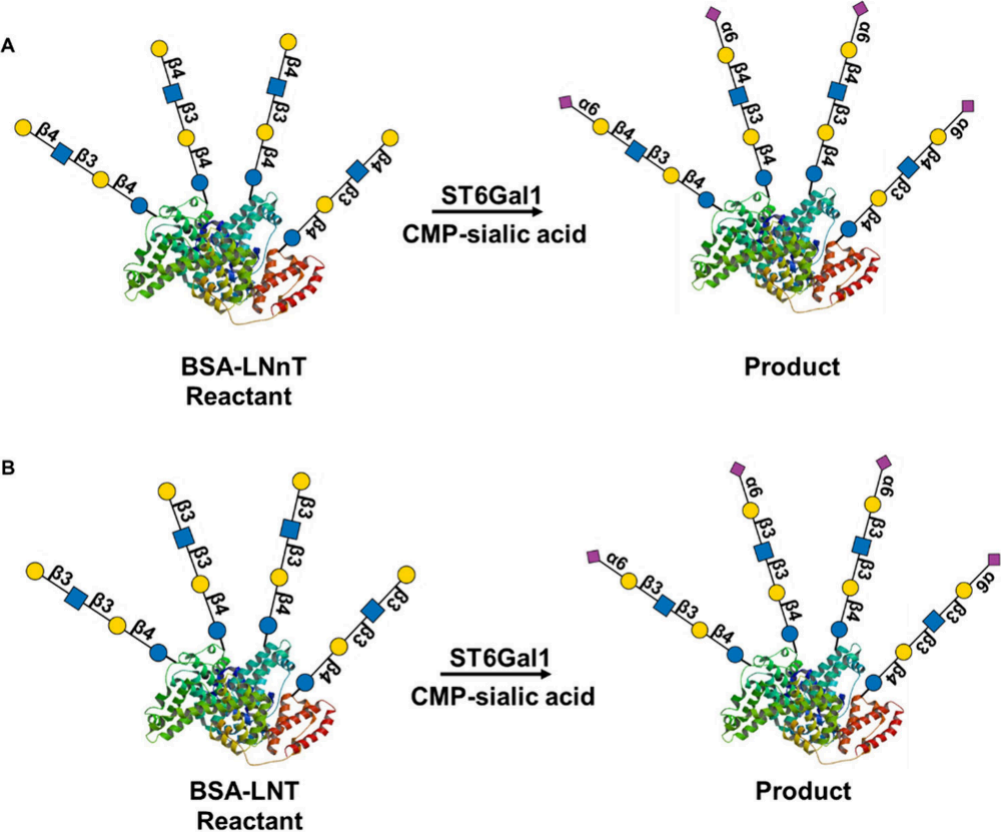
Schematic diagram of the reactant and product structure: (A) BSA-LNnT and (B) BSA-LNT.

**Figure 9:**
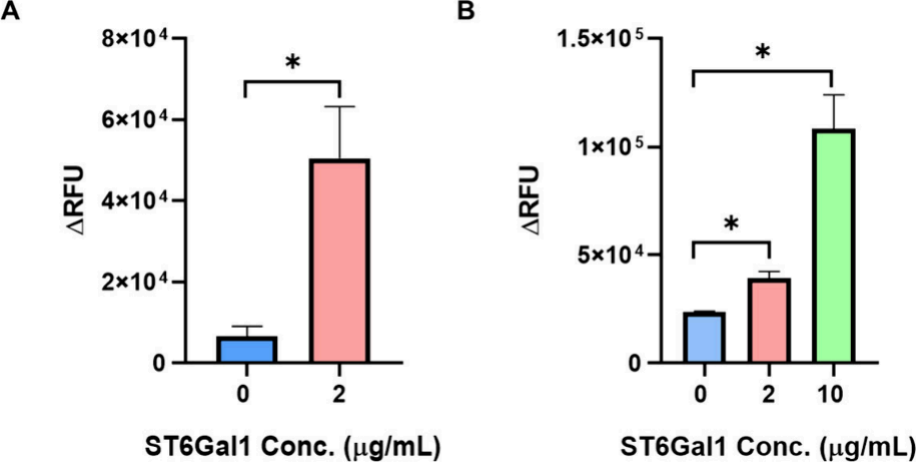
Demonstration of analysis of recombinant ST6Gal1 activity using the ELISA assay. (A) Detection with fluorescence-labeled SNA. (B) Detection with PBMC3–02 antibody and fluorescein-labeled secondary antibody. Error bar represents means ± 1 SD from analyses of two independent experiments (ns *p* > 0.05, * *p* ≤ 0.05).

Using this approach, we determined the *K*_m_ of ST6Gal1 for the donor CMP-Sia using a fixed concentration of ST6Gal1 (Figure 10A). The *K*_m_ of CMP-Sia for the recombinant human ST6Gal1 in this assay format, detected with fluorescently labeled SNA, was calculated as 141.4 μM. This compares favorably to the reported *K*_m_ of CMP-Sia of 42.7 μM for the recombinant rat ST6Gal1.^64^ Thus, the CMP-Sia donor concentration was fixed at 500 μM to ensure nonlimiting substrate concentration. As expected, a linear relationship was observed between the signal and the ST6Gal1 concentration. The fitted equation was *Y* = 29870*X* + 7475, with an *R* ^2^ value of 0.9917, indicating the potential application of this approach for diagnostics (Figure 10B).^65^ To assess the binding specificity after treatment with ST6Gal1, a lectin recognition assay was performed. Figure 11A shows that lectins HPA, GSL-I, and ConA did not bind to the ST6Gal1-treated samples, whereas specific binding by SNA was observed. These results provide further evidence that ST6Gal1 modified the LNnT to form α2–6 glycosidic linkages on the galactose residue.

**Figure 10:**
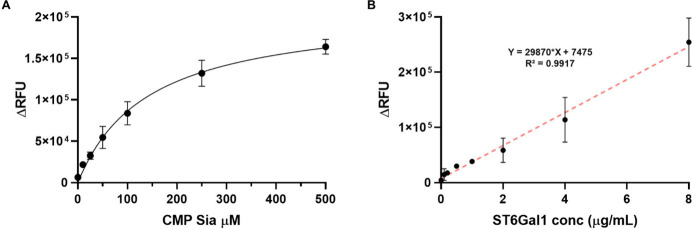
Optimization of the neoglycoprotein ELISA assay. (A) Evaluation of various concentrations of CMP-Sia. (B) Enzymatic activity as a function of the dilution factor of ST6Gal1. The data are presented as an average RFU of two replicates; each experiment was repeated two times.

**Figure 11:**
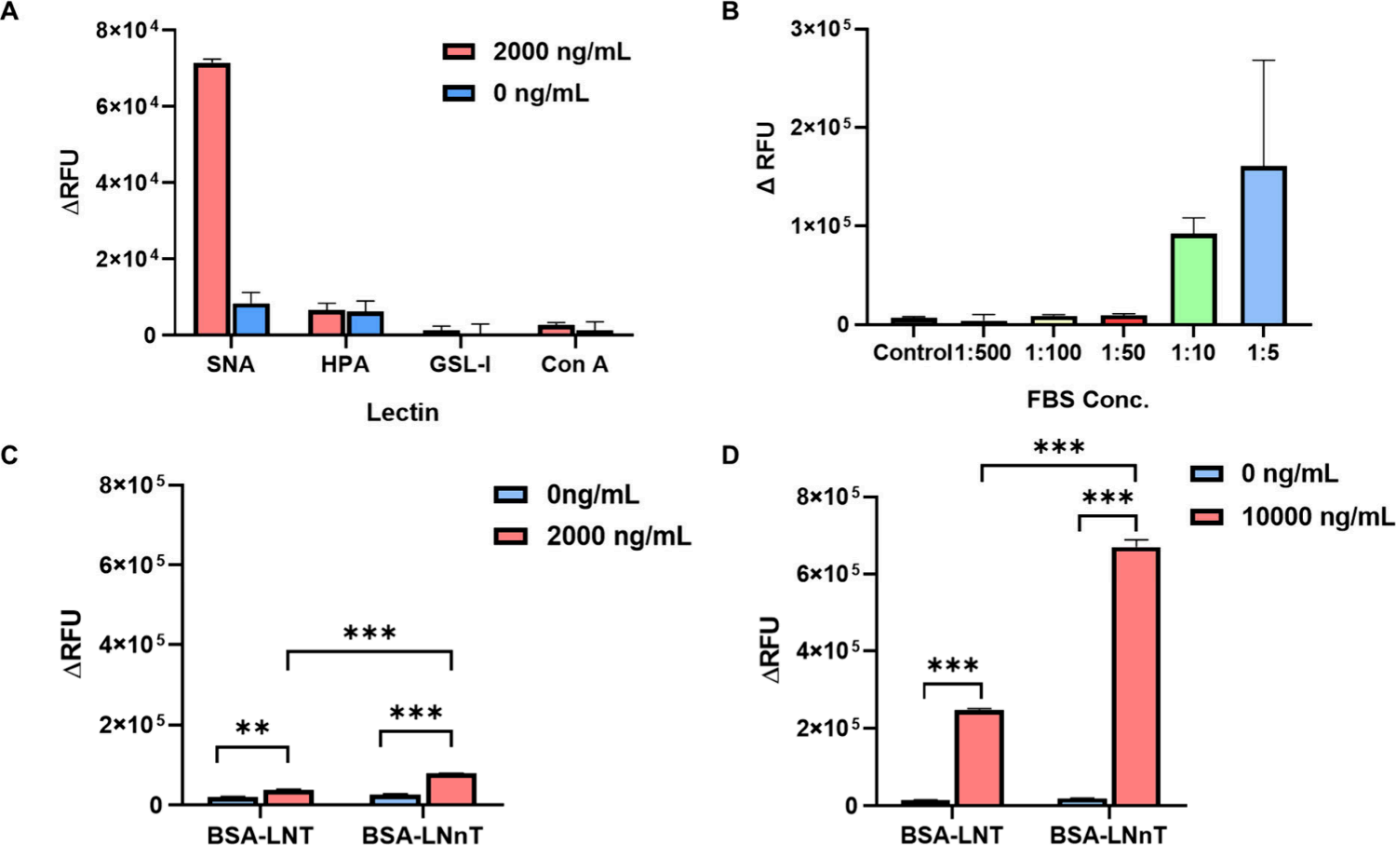
Analysis of ST6Gal1 activity using a neoglycoprotein ELISA assay. (A) Specificity test. (B) Evaluation of ST6Gal1 activity in the FBS sample. Evaluation of ST6Gal1 activity with BSA-LNT and BSA-LNnT in (C) 2,000 ng/mL and (D) 10,000 ng/mL of enzyme. The data are presented as an average RFU of two replicates; each experiment was repeated two times. Error bars = ±1 SD (ns *p* > 0.05, * *p* ≤ 0.05, ** *p* ≤ 0.01, *** *p* ≤ 0.001).

Mammalian serum contains soluble, active sialyltransferases, including ST6Gal1, which are released from cells by proteolysis.^66,67^ To assess the application of an ELISA assay for enzyme activity in biological samples, we measured the ST6Gal1 activity in fetal bovine serum (FBS). A significant signal was observed in samples diluted 1:10 and 1:5, suggesting that the presented neoglycoprotein ELISA is an acceptor of serum ST6Gal1 (Figure 11B). In an additional experiment, we aimed to gain insight on ST6Gal1 activity for different substrates. For this purpose, Lacto-*N*-tetraose (LNT) (Galβ1–3GlcNAcβ1–3Galβ1–4Glc) was used to prepare neoglycoproteins using the same strategy. LNT is an isomer of LNnT, which differs only in the type of glycosidic linkage within nonreducing end disaccharides. Consequently, we tested the ST6Gal1 activity with BSA-LNT and BSA-LNnT (Figure 8). In terms of activity, ST6Gal1 demonstrated activity primarily for BSA-LNnT but also modified BSA-LNT at different concentrations of ST6Gal1 (Figure 11C,D). This indicates that ST6Gal1 exhibits higher activity for BSA-LNnT compared with BSA-LNT, consistent with the specificity of the enzyme for type 2 LacNAc. Interestingly, as we noted earlier, recombinant ST6Gal1 can weakly modify type 1 LacNAc on glycan microarrays, where hundreds of different glycan acceptors are all tested simultaneously.^68^ We conclude that neoglycoproteins prepared by this general method have versatile applications in exploring GBP–glycan interactions.

## Discussion

Here we have provided a general approach for glycan derivatization useful in preparing various platforms to explore protein–glycan interactions. Unlike previously reported linkers used for glycan derivatization via reductive amination,^39,41,42^ which permanently maintain the glycan in their ring-open form and potentially affect lectin binding and glycan recognition, our approach leverages the novel multifunctional MTZ linker, which preserves a closed ring at the glycan reducing end. Although other *N*-alkyl-O-methyl oxyamine linkers also preserve glycan integrity,^37,38,40^ the multifunctional MTZ linker incorporates a tetrazine group, eliminating the need for additional synthesis steps to introduce a fluorescent label. Importantly, the click reaction between tetrazine and TCO enables rapid, straightforward, and highly efficient conjugation across various platforms compared to amine-mediated conjugation methods. In contrast, our previously reported F-MAPA linker required an additional deprotection and purification step prior to its application on different platforms.^26^ These approaches will be invaluable to generally explore the complex protein–glycan interactome found in nature, in which all organisms generate glycan-binding proteins and recognize glycans in their own glycoconjugates as well as those of other organisms.^69^

Studies of glycan recognition by antibodies is of special importance, as there is a growing appreciation that human serum contains a tremendous variety of antiglycan antibodies.^13,17,18,70–73^ Furthermore, the assessment of antibodies with specificity for blood group antigens (isohemagglutinins) and potentially to other glycans is of diagnostic relevance for the detection and classification of different PAD entities.^16,74^ However, studies of serum antibody binding to glycans, which often require high-throughput technologies, are difficult to conduct with glycan microarrays^18,75^ as they can require relatively large amounts of materials, both glycans and antibodies, and coupling efficiencies of glycans are not highly efficient, nor easily adaptable for large numbers of samples with multiple serum dilutions, and not readily amenable to examination of all classes of immunoglobulin reactivities. These platforms are also largely confined to the research space, making it difficult to rapidly adapt this technology to clinical practice.

To address these limitations, there have been a few studies demonstrating the utility of Luminex-based multiplex glycan arrays and bead-based automated approaches which can provide very useful information.^76–81^ This technology relies on microspheres or magnetic microspheres that are color-coded into numerous spectrally distinct sets, also known as regions.^79,82^ Luminex bead regions, each coated with different glycans, have been used to study GBP–glycan interactions.^77–79,82^ However, these approaches to conjugate glycans to the Luminex beads typically require multiple buffer solutions and washing steps, resulting in inefficiencies in time and consumed glycans. Here, the procedure we have developed promotes rapid, simple, and efficient procedures to conjugate the glycan and Luminex beads through a strong noncovalent binding between biotin and avidin, as well as the click-based covalent binding between TCO and tetrazine, both of which form bonds rapidly.^83,84^ Therefore, the conjugation is completed in 2 h by using a single buffer solution. Furthermore, the use of commercially available avidin Luminex beads ensures the formation of relatively homogeneous and high-density glycan Luminex beads. The rapidity and ease of forming these Luminex-based multiplex glycan arrays using biotin/avidin reaction and click chemistry are major advantages over other technologies using less efficient coupling strategies.^78^ Considering the common adoption of Luminex technology in modern diagnostic approaches, integrating this method into clinical practice is likely to be significantly more practical and achievable compared to other alternative strategies.

Antiglycan antibodies in human serum play an integral role in human immune defense, homeostasis, and autoimmunity.^58^ Antibody profiling in healthy subjects provides fundamental information about the immune system and enhances our insights into how the immune system responds to infection, disease onset and progression, vaccination, and managing treatments.^17^ While prior studies have profiled IgG and IgM responses in serum, there is a lack of studies reporting on titers of IgG, IgM, and IgA antiglycan antibodies in individual human serum samples. IgA (mainly IgA1) is the second most common immunoglobulin in human serum, while IgA2 is present in secretions. IgA2 is known to have antiglycan binding specificity toward pathogens and commensal microbes,^85^ but there are few studies on serum IgA1 binding to glycans. We observed relatively low titer binding of serum IgA to blood group antigens in some samples, e.g., samples 8 and 9 (Figure S9), which matched to a great degree the IgG reactivity in the same samples, whereas other samples, e.g., sample 6, exhibited extremely low to no IgA binding to glycans in the Luminex bead array (Figure S9). The roles of IgA in serum toward glycan antigens, which might be displayed on microbes or invasive organisms, are an area needing further study, but these results suggest highly varied and different individual responses.

In particular, our study provides interesting insights into the immune status and history of the individual. Detailed characterization of ABO antibodies offers clinical guidance for pretransplant extracorporeal antibody removal therapy and reduces clinical unpredictability in ABOi transplantation.^86^ Our findings revealed that the repertoire of antiglycan antibodies in each individual tested is unique. Differences in antibody levels between different individuals can indicate variations in immune responses due to factors like age, blood type, health status, exposure to pathogens, or genetic background.^87^ Of course, to have a better understanding of antiglycan antibody patterns larger samples with various conditions would need to be analyzed.

Neoglycoproteins have been widely used in vaccine development,^88,89^ diagnostics,^90–93^ fluorescence probes,^94^ and antibody analysis.^75^ We synthesized a library of neoglycoproteins to gain insight into the ability to probe the interaction between glycans and GBPs. We present a strategy that is simple and efficient and achieves comparable glycan loading to different reported methods and commercial conjugation kits.^38,57,95–97^ The resulting neoglycoprotein library was successfully printed on two different slides and evaluated with plant lectins. The newly discovered patterns of binding suggest that the neoglycoprotein microarray is a complement to other glycan microarray platforms to study glycan–protein interactions.

Moreover, glycosyltransferases catalyzed nucleotide sugars to generate the desired glycosidic linkage. Glycosyltransferases not only play an important role in cell–cell interaction, immune response, and progression of disease but also serve as an indispensable tool for complex glycan structure synthesis.^98,99^ Determining the activity of glycosyltransferases and specific product formation is typically difficult and has several limitations, including the requirement of complex methodologies, such as novel nucleotide sugar derivatives, simple monosaccharide acceptors, HPLC, and mass spectrometry.^98,100^ We have also recently used glycan microarrays to assay enzyme activities, in particular the sialyltransferase ST6Gal1,^68^ but these require precious reagents, e.g. individual slide arrays, and are not amenable to detailed enzymatic studies.

The feasibility of using neoglycoproteins to assay glycosyltransferases was demonstrated in our earlier studies using neoglycoproteins to assay serum β1,4-galactosyltransferases using GlcNAc-BSA conjugates in ELISA-type formats,^101^ but the formation of such conjugates was inefficient and limited to simple sugars and thus was not pursued. Here we explored the use of novel MTZ-derived neoglycoproteins to measure the activity of the sialyltransferase ST6Gal1. This enzyme is commonly expressed in mammalian cells and is required to generate addition of α2,6-sialyl linkage to terminal *N*-acetyllactosamine chain of glycoproteins and glycolipids.^102^ In addition, ST6Gal1, as a biomarker, has been implicated in the progression of numerous diseases, particularly cancer.^103–109^ This approach is easily adaptable to other donor nucleotide sugars and other reagents to detect product formation using specific neoglycoprotein acceptors and controls. The success of this approach raises the potential of using such high-density neoglycoproteins to assay multiple glycosyltransferases in single serum samples for comparative analyses with diagnostic implications.

In summary, we present a versatile platform for glycan derivatization to Luminex beads and the preparation of neoglycoproteins. The power of these methods was demonstrated by various applications, including recognition by antibodies and lectins and use in measurements of glycosyltransferase activity. More importantly, this method can be easily adapted for the conjugation of various avidin- or amine-carrying biomolecules, and it can also be used to couple with other functional-group-carrying biomolecules by simply modifying the biotin and NHS ester linker, although the reaction conditions may need adjustment. These new approaches for glycan conjugation greatly expand the toolbox of methods available to explore glycan–GBP interactions.

## Methods

### Derivatization of Free Oligosaccharides with MTZ HCl Salt

A 0.5 M solution of sodium acetate (NaOAc) and 1 M 2-amino-5-methoxybenzoic acid (2-AM) in DMSO/AcOH (7:3 V/V) were freshly prepared. To 100 μg of glycan, 100 equiv of NaOAc, 100 equiv of MTZ HCl salt, and 25 equiv of 2-AM were added, with the final volume adjusted to 50 μL. The mixture was vortexed and heated at 65 °C on a shaker for 4 h. After the reaction, 20 vols of ethyl acetate were added, vortexed, and frozen at −20 °C for 20 min to precipitate the glycan-MTZ. The precipitate was collected by centrifugation at 10K relative centrifugal force (RCF) for 10 min, and ethyl acetate was removed. The sample was dried in a centrifugal evaporator. Dried samples were dissolved in 200 μL of Milli-Q water and loaded on a C18 SPE cartridge (500 mg). After sample loading, the cartridge was washed with 6 × 1 mL of Milli-Q water, and glycan-MTZ was eluted with 20% acetonitrile. Fractions were combined and lyophilized. Glycan-MTZ was quantified by absorption at 520 nm. The UV–vis absorption and fluorescence spectra were measured using a SpectraMax i3x with 100 μL of MTZ and MTZ-LNnT in Milli-Q water per well.

### Cleavage of MTZ Linker

LNnT-MTZ (2.1 mg, 2.12 μmol) was dissolved in 100 μL of water containing NCS (6.35 μmol, 3 equiv). The mixture was stirred at 50 °C for 2 h, and the MALDI-TOF showed no LNnT-MTZ remaining. The mixture was purified by a P2 biogel filtration. The fractions were combined and lyophilized to afford LNnT which is weighed by analytical balance (1.3 mg, 86.78%).

### Conjugation of Glycan-MTZ with Luminex Beads

Briefly, the stock MagPlexAvidin beads (2.5 × 10^6^ beads/mL) were vigorously vortexed for 30 s, and 200 μL (5 × 10^5^ beads) were transferred into a 1.7 mL microcentrifuge tube. The beads were separated by a magnetic separator and washed with 200 μL of PBS-TBN (containing 0.1% BSA, 0.02% Tween 20, and 0.05% NaN_3_) once. Beads were resuspended in 90 μL of PBS-TBN, to which Biotin-PEG11-TCO (10 μL, 250 μg/mL) was added, and the beads were incubated on a rotator at RT for 30 min. After that, the beads were separated by magnetic separator and washed with 200 μL of PBS-TBN two times. The recovered beads were resuspended in 200 μL of PBS-TBN, and 1 μL of beads was mixed with Sulfo-Cy3-Tetrazine (1 μL, 0.1 mM) in a final volume of 50 μL PBS-TBN. The mixture was incubated for 1 h to confirm the conjugation of Biotin-PEG11-TCO with the beads. After 1 h, the beads were separated by magnetic separator and washed with 200 μL of PBS-TBN two times. The beads were resuspended in 100 μL of PBS-TBN and were transferred to 96-well plate with a clear bottom (Corning REF 3880). The samples were scanned using an ImageXpress Pico microscope under Cy5 and Cy3 channels. The remaining beads were separated by magnetic separator and resuspended in 90 μL of PBS-TBN, to which glycan-MTZ (10 μL, 5 μg) was added. The beads were incubated on a rotator at RT for 1.5 h. After that, the beads were separated by magnetic separator and washed with 200 μL of PBS-TBN two times. Finally, the beads were suspended in 400 μL of PBS-TBN and stored in the dark at 4 °C. The count of beads was identified with the ImageXpress Pico microscope (Molecular Devices), and the stored concentration of beads was adjusted to 1 × 10^6^ beads/mL.

### Recognition of Glycans by Plant Lectins

Plant lectins (i.e., AAL, GSL-I, DBA, HPA, PNA, UEA-I, Vector Laboratories) conjugated with APDye 532 were evaluated in this assay. Before the assay, 1000 beads/well for each glycan were mixed in a 1.7 mL microcentrifuge tube to create an array. 20 μL of mixed beads suspension was added to each well in 96-well plate (white, round-bottom), followed by the addition of 30 μL of PBS-TBN containing 1 μg of the lectin. The mixture was incubated on a shaker at room temperature for 1 h. The mixed beads were separated using a magnetic separator for 3 min, after which the supernatant was carefully removed. The wells were washed twice with PBS-TBN. The beads were resuspended in 100 μL of PBS-TBN, and the fluorescence intensities were measured on a Luminex FLEXMAP 3D instrument.

### Recognition of Glycans by Antiglycan Antibodies

Antiglycan monoclonal antibodies were produced in our laboratory using immunized lamprey through the smart antiglycan reagents (SAGRs) technology.^55,110^ Briefly, 1000 beads/well for each glycan were mixed in a 1.7 mL microcentrifuge tube to create an array. 20 μL of mixed beads suspension was added to each well of a 96-well plate (white, round-bottom), followed by the addition of 30 μL of PBS-TBN containing 0.5 μg antiglycan antibodies. The mixture was incubated on a shaker at room temperature for 1 h. The 96-well plate was placed on a magnetic separator, and separation was allowed to occur for 3 min; the supernatant was removed, and the wells were washed with PBS-TBN (twice). After washing, 50 μL of PBS-TBN containing 0.5 μg of Alexa Flour 546 nm labeled goat-antirat IgG was added to each well and incubated for 1 h. The plate was washed twice with PBS-TBN; beads were resuspended in 100 μL of PBS-TBN, and the fluorescence intensities were measured on a Luminex FLEXMAP 3D instrument.

### Profiling of Antiglycan lgG, IgM, and IgA Antibodies in Human Serum Sample

Briefly, 1000 beads/well for each glycan were mixed in a 1.7 mL microcentrifuge tube to create an array. 10 μL of mixed beads suspension and 30 μL of PBS-TBN were added to each well of a white, round-bottom 96-well plate, followed by the addition of 10 μL of human serum sample with serial dilution (1:100, 1:200, 1:400, 1:800, 1:1600, 1:3200, 1:6400). The mixture was incubated on a shaker at room temperature for 1 h. The 96-well plate was placed on a magnetic separator for 3 min to allow separation. The supernatant was removed, and the wells were washed twice with PBS-TBN. After washing, 50 μL of PBS-TBN containing PE-labeled antihuman IgG, IgM, or IgA (diluted 1:100 from the stock solution) was added to the corresponding wells and incubated for 1 h. The plate was washed twice with PBS-TBN, and beads were resuspended in 100 μL of PBS-TBN and the fluorescence intensities were measured on a Luminex FLEXMAP 3D instrument.

### Preparation of Copper-free BSA

BSA (2 g) was dissolved in 40 mL of BBS-EDTA (50 mM borate, 25 mM NaCl, 2 mM EDTA, pH 8.0) and incubated at 4 °C for 16 h. The protein was subsequently dialyzed (10 000 Da MWCO, 4 × 15 mL) with BBS-EDTA and left for another 5 h at 21 °C. After that, the protein was dialyzed (10 000 Da MWCO, 4 × 15 mL) with BBS without EDTA and stored at −20 °C.

### BSA Conjugated with TCO-PEG6-NHS Ester

BSA conjugated with different equivalents (10, 25, 50, 100 equiv) *trans*-cyclooctene–PEG6–NHS ester (TCO-PEG6-NHS ester) were prepared by the same procedure. To a solution of copper-free BSA (2 mg, 190 μL) was added TCO–PEG6–NHS (60 μL, 100 equiv, 50 mM in DMSO), and the solution was incubated for 7 h at RT. The protein was purified via ultrafiltration (30 kDa, 6 × 2 mL) into Milli-Q water and stored at −80 °C. Protein concentration was identified by a Nanodrop One^C^ spectrophotometer, and the resulting bioconjugates were assessed by Bruker ultrafleXtreme MALDI TOF/TOF mass spectrometer. 2,5-Dihydroxybenzoic acid (Sigma-Aldrich) was used as the MALDI matrix and was prepared by dissolving 15 mg in 1 mL of 70/30 acetonitrile/water with 0.1% trifluoroacetic acid.

### General Preparation of Neoglycoproteins

A 100 μg portion of BSA-PEG6-TCO_46_ was mixed with glycan-MTZ (100 equiv) in Milli-Q water with a final volume of 125 μL. This solution was incubated overnight at room temperature. The protein was purified via ultrafiltration (30 kDa, 6 × 2 mL) into Milli-Q water and stored at −20 °C. Protein concentration was identified by BCA assay. Modification with different equivalents (10–500 equiv) 3′-sialyllactose-MTZ was achieved by the same protocol. Neoglycoproteins were analyzed by a Bruker ultrafleXtreme MALDI TOF/TOF mass spectrometer.

### Microarray Printing

Neoglycoproteins and BSA controls were diluted in PBS buffer (pH 7.4) to a concentration of 100 μg/mL and were plated into a 384-well plate, which was interfaced onto the sciFLEXARRAYER SX microarray printer (Scienion). A 16 subarray layout was set up on the printer to print 1 drop of each probe at 330 pL (within 5% variation) in 4 replicates per array. Nitrocellulose slides (Oncyte SuperNOVA from Grace Bio-Laboratories) and epoxy slides (sciCHIP EPOXY from Scienion) were placed on the deck for printing, and the humidity was adjusted to 70% prior to printing. Following the printing, the slides were kept on the deck for 1 h at room temperature, after which the nitrocellulose slides were transferred to a slide tube and placed in the cold room overnight, while the epoxy slides were left on the deck at 70% humidity overnight. The nitrocellulose slides were blocked using SuperG Plus protein preservative buffer (Grace Bio-Laboratories) following the manufacturer’s protocol. The epoxy slides were blocked with 50 mM ethanolamine in 100 mM borate buffer (pH 8.5) for 1 h, followed by dip-washing 10× in PBST (PBS + 0.05% Tween-20), dip-washing 10× in water, and centrifuged to dryness. The slides were put into separate tubes and stored at −20 °C prior to use.

### Microarray Analysis

Biotinylated lectins AAL and GSL-I were obtained from Vector Laboratories, and biotinylated HPA obtained from Sigma-Aldrich was used to characterize the binding on the microarrays. Briefly, slides were taken out of the freezer, and the 16-well ProPlate (Grace Bio-Laboratories) was put on them. TSMW buffer (20 mM Tris-HCl, 150 mM sodium chloride, 0.2 mM calcium chloride, 0.2 mM magnesium chloride, and 0.05% Tween-20) was put on the slides for 30 min. The samples were diluted in TSMBB (TSMW buffer +1% BSA) for the epoxy slides or Super G BB (TSMBB buffer +1% v/v SuperG Blocking Buffer (Grace Biolabs) for the nitrocellulose slides. The lectins were diluted to a concentration of 20 μg/mL. The buffer was aspirated out, and the sample was put in the wells for incubation for 1 h at room temperature while shaking on an orbital shaker. The arrays were washed with TSMW buffer 4 times and TSM buffer (TSMW buffer without 0.05% Tween-20), following which the secondary (Streptavidin-Cy5 at 0.5 μg/mL for the biotinylated lectins) was incubated in TSMBB for 1 h at room temperature over the array and kept on the orbital shaker. After which, washes were done with 4× TSMW buffer, 4× TSM buffer, and 4× milli-Q water and dried. The slides were scanned using the GenePix 4400A scanner (Molecular Devices) with a PMT setting of 450 and a laser power of 70 to obtain the raw image. The gal file generated by the printer was used to align the spots and quantify the signal intensity of the spots using GenePix Pro 7.3 software to produce the GPR file. Microsoft Excel was used to average the signal of 4 spots to get the average RFU (relative fluorescence units), standard deviation, and %CV.

### Optimization of Neoglycoprotein Immobilized with 96-Well Plate

Briefly, neoglycoprotein BSA-LNnT with different concentrations (10–500 ng/well, 50 μL, in BBS pH 8.0) was immobilized 24 h at 4 °C in a 96-well plate (Thermo Scientific, 437111). BSA-PEG6-TCO_46_ was defined as control (0 ng/well). The following day, the wells were washed with washing buffer (200 μL, 0.1% Tween-20 in PBS) 3 times, blocked with 20 mg/mL BSA in PBS (100 μL) for 1 h at room temperature, and washed three times with 200 μL, 0.1% Tween-20 in PBS. 50 μL of lectin RCA labeled with APdye-532 (10 μg/mL, in PBS) was added to each well and incubated for 1 h. After incubation, the wells were washed three times with 200 μL of washing buffer. The fluorescence signal was measured at 530 nm using a SpectraMax i3x instrument (Molecular Devices) with 100 μL of PBS per well.

### General Procedure of Neoglycoprotein ELISA

Briefly, neoglycoprotein BSA-LNnT (250 ng/well, 50 μL, in BBS pH 8.0) was immobilized 24 h at 4 °C in a 96-well plate (Thermo Scientific, 437111). The following day, the wells was washed with washing buffer (200 μL, 0.1% Tween-20 in PBS) 3 times and blocked with 20 mg/mL BSA in PBS (100 μL) for 1 h at room temperature and washed three times with 200 μL of 0.1% Tween-20 in PBS.

### Feasibility of Evaluation of ST6Gal1 Sialyltransferases Based on ELISA

After coating with BSA-LNnT, 30 μL of MES buffer (pH 6.5), 10 μL of ST6Gal1 sialyltransferases (10 μg/mL), and 10 μL of substrate (5 mM) were added into each well. Additionally, a blank containing no ST6Gal1 control was included. The microplate was incubated at 37 °C for 2 h and washed with washing buffer three times. 50 μL of SNA lectin labeled with APdye-532 (10 μg/mL, in PBS) was added into each well and incubated for 1 h at RT. After incubation, the wells were washed three times with 200 μL of washing buffer. The fluorescence signal was measured at 530 nm using a SpectraMax i3x instrument with 100 μL of PBS per well. Background subtraction was performed as follows: signals obtained from BSA-LNnT treated with PBS were subtracted from the signals obtained from the corresponding assay treated with lectin SNA labeled with APdye-532.

The same procedure was performed for the PBMC3–02 antibody (produced in our lab). Briefly, the BSA-LNnT coated plate was treated with 10 μL of ST6Gal1 (10 and 50 μg/mL) and CMP-sialic acid (2.5 mM) as described above. After, 50 μL of PBMC3–02 antibody (10 μg/mL, in PBS) was added into each well and incubated for 1 h at RT. After incubation, the wells were washed three times with 200 μL of washing buffer. Secondary Alexa Fluor-488 labeled goat antirat antibody was added and incubated 1 h at RT followed by washing 3 times with washing buffer. The fluorescence signal was measured at 488 nm using a SpectraMax i3x instrument with 100 μL of PBS per well. Background subtraction was performed as follows: signals obtained from BSA-LNnT treated with PBS were subtracted from the signals obtained from the corresponding assay treated with Alexa Fluor-488 labeled goat antirat antibody.

### Optimization of Neoglycoprotein ELISA

30 μL of MES buffer (pH 6.5) and 10 μL of the different concentrations of CMP-sialic acid were added into the BSA-LNnT coated plate with fixed concentrations of ST6Gal1 (10 μL, 10 μg/mL). The microplate was incubated at 37 °C for 2 h and washed with washing buffer three times. 50 μL of SNA lectin labeled with APdye-532 (10 μg/mL, in PBS) was added into each well and incubated for 1 h at RT. After incubation, the wells were washed three times with 200 μL of washing buffer. Fluorescence signal measurements and background subtraction were performed as previously described. The same procedure was performed with different ST6Gal1 and fixed concentration of CMP-sialic acid (2.5 mM).

### Specificity Assay

After coating the plate with BSA-LNnT, it was treated with 30 μL of MES buffer (pH 6.5), 10 μL of ST6Gal1 sialyltransferase (10 μg/mL), and 10 μL of CMP-sialic acid substrate (2.5 mM), followed by incubation at 37 °C for 2 h. The plate was washed three times with washing buffer. 50 μL of lectin labeled with APdye-532 (10 μg/mL, in PBS) was added into each well and incubated for 1 h at RT. After incubation, the wells were washed three times with 200 μL of washing buffer. Fluorescence signal measurement and background subtraction were performed as previously described.

### Evaluation of ST6Gal1 Sialyltransferases in Fetal Bovine Serum (FBS) Sample

30 μL of MES buffer (pH 6.5) and 10 μL of the different dilutions of FBS sample (1:5, 1:10, 1:50, 1:100, 1:500) were added into BSA-LNnT coated plate with fixed concentrations of CMP-sialic acid substrate (2.5 mM). The microplate was incubated at 37 °C for 2 h and washed with washing buffer three times. SNA lectin labeled with APdye-532 recognition, washing step, and measurement were performed as previously described.

### Evaluation of ST6Gal1 Sialyltransferases with BSA-LNT and BSA-LNnT

Briefly, BSA-LNT and BSA-LNnT coated plate was treated with 10 μL of ST6Gal1 (10 and 50 μg/mL), 10 μL of CMP-sialic acid (2.5 mM), and 30 μL of MES buffer (pH 6.5). The microplate was incubated at 37 °C for 2 h and washed with washing buffer three times. Lectin SNA labeled with APdye-532 was added; the washing step and measurement were performed as previously described.

## Supplementary Material



## Data Availability

All the relevant data are available through the Harvard Dataverse: 10.7910/DVN/JQYJDG.

## Notes

Details of synthesis of MTZ, supporting figures and tables, NMR, MALDI and LC-ESI-MS spectra (PDF)
