# Supplementary material for: Novel Click Coupling Chemistry to Explore Glycan Recognition
Source: ACS Cent Sci. 2025 Apr 23;11(5):753–69. doi: 10.1021/acscentsci.4c02124 (PMC12123462; doi:10.1021/acscentsci.4c02124)

Supplementary Information

Novel Click Coupling Chemistry to Explore Glycan Recognition

Tianwei Jia[1], Akul Y. Mehta[1], Catherine A. Tilton[1], Ea Kristine Clarisse Tulin[1], Lauren E. Pepi[1], Lukas Muerner[1,2], Stephan von Gunten[2], Jamie Heimburg-Molinaro[1], Sean R. Stowell[3], and Richard D. Cummings[1]*

[1]Department of Surgery, Beth Israel Deaconess Medical Center, Harvard Medical School, National Center for Functional Glycomics, CLS 11087-3 Blackfan Circle, Boston, Massachusetts 02115, USA.

[2]Institute of Pharmacology, University of Bern, Inselspital, INO-F, Bern 3010, Switzerland.

[3]Joint Program in Transfusion Medicine, Brigham and Women's Hospital, Harvard Medical School, 630E New Research Building, 77 Avenue Louis Pasteur, Boston, MA 02115, USA.

*Corresponding author: Richard D. Cummings at rcummin1@bidmc.harvard.edu

Experimental Section

Materials and Equipment

3-aminopropionaldehyde diethyl acetal and 7Methoxyamine hydrochloride purchased from Santa Cruz Biotechnology. Sodium cyanoborohydride ($NaBH_3CN$) purchased from Chem-Impex. 4-(6-Methyl-1,2,4,5-tetrazin-3-yl)benzoic acid (Tetrazine acid) purchased from Ambeed Inc. *N,N*-Diisopropylethylamine (DIPEA) purchased from Oakwood Chemical. HATU purchased from AA Blocks. 4M HCl in 1,4-dioxane, piperidine and Fmoc-Cl purchased from Sigma-Aldrich. Di-tert-butyl Dicarbonate purchased from TCI. CMP-Sialic acid (sodium salt) purchased from MedChem Express. TCO-PEG6-NHS ester, Biotin-PEG11-TCO and Sulfo-Cy3-Tetrazine were purchased from Broadpharm. Blood group glycans were purchased from ELICITYL. All the plant lectins used in the assay were purchased from Vector Labs. Alexa Fluor 546 labeled goat-anti-rat IgG (cat# 737671) was purchased from Invitrogen. Anti-human IgG (cat# 109-116-190), IgM (cat# 109-116-129) and IgA (cat# 109-115-011) were purchased from Jackson ImmunoResearch Labs. BSA was purchased from Fisher Bioreagents. Human serum samples used in the study were obtained from Clinical Research Center at BIDMC. A- and O- blood group type samples were obtained from Sean R. Stowell's laboratory at Brigham & Women's Hospital under an approved BWH IRB protocol (#2023P0003321) and a BIDMC exempt IRB protocol (#2016P000014). Ultrapure water obtained from a Millipore water purification system (18.2 MΩ cm^{-1}, Milli-Q, Merck Millipore, Darmstadt, Germany) was used in all experiments. Fluorescence intensity was detected by Molecular Devices SpectraMax i3x Multi-Mode microplate reader. The Luminex beads image was procced by Molecular Devices ImageXpress pico. Luminex assays were measured on a Luminex FLEXMAP 3D instrument. An Ultraflextreme MALDI-TOF/TOF system from Bruker was used for MALDI-TOF MS analysis of the glycans and their conjugates. 2,5-Dihydroxybenzoic acid (15 mg/ml in ACN: H_2O = 7:3 with 0.1% TFA) was used as the matrix. ESI-MS analysis was performed on a TSQ Altis MS from Thermo Fisher Scientific coupled to a Vanquish LC system equipped with a PGC column (cat# 35003-101030). ^{1}H and ^{13}C NMR spectra were recorded on Varian Mercury 400 MHz.

Synthesis of MTZ linker

Synthesis of Compound 2. To a solution of 3-aminopropionaldehyde diethyl acetal (2 g, 13.59 mmol) and Fmoc-Cl (3.69g, 14,26mmol) in DCM was added DIPEA (4.73 mL, 27.17 mmol) at 0°C. The solution was allowed to warm to room temperature and stirred for overnight. The mixture was washed with saturated NaCl, dried with Na_2SO_4 and concentrated to provide the crude compound 1 with quantitative yield which is used directly in the next step.

To a solution of compound 1 in DCM, 4 M HCl in dioxane (7.44 mL) was added and the solution stirred at 0°C. The reaction was monitored by TLC until the disappearance of

compound 1. After completion of reaction, the mixture was washed with saturated $NaHCO_3$ and organic phases were extracted with DCM, dried with Na_2SO_4. The crude product was concentrated under reduced pressure and purified by flash chromatography over silica gel using (hexane/ethyl acetate, from 0 to 35 %) as the eluent to afford compound 2 as a white solid (3 g, 75%). ^{1}H NMR (400 MHz, $CDCl_3$) δ 9.80 (s, 1H), 7.76 (d, *J* = 7.5 Hz, 2H), 7.57 (d, *J* = 7.5, 1.0 Hz, 2H), 7.41-7.38 (m, 2H), 7.33-7.29 (m, 2H), 5.18 (s, 1H), 4.39 (d, *J* = 6.9 Hz, 2H), 4.19 (t, *J* = 6.9 Hz, 1H), 3.49 (d, *J* = 6.0 Hz, 2H), 2.73 (t, *J* = 5.8 Hz, 2H). ^{13}C NMR (100 MHz, $CDCl_3$) δ 196.41, 151.57, 139.12, 136.57, 122.94, 122.28, 120.25, 115.22, 61.94, 42.50, 39.29, 29.72. See NMR spectra in **Figure S15**.

Synthesis of Compound 3. To a solution of compound 2 (3 g, 10.16 mmol) and $CH_3ONH_2 \cdot HCl$ (1.7 g, 20.32 mmol) in DCM, DIPEA (7.08 mL, 40.63 mmol) was added. The mixture was stirred overnight at room temperature and the mixture was washed with saturated NaCl, dried with Na_2SO_4 and concentrated to provide the crude imine intermediate. The imine intermediate was dissolved in DCM/HOAc (4/1, v/v,), and then $NaBH_3CN$ (958.96 mg, 15.26 mmol) was added at 0°C and stirred for 1 h. The mixture was washed with saturated $NaHCO_3$, and organic phases were extracted with DCM, dried with Na_2SO_4. The crude product was concentrated under reduced pressure and purified by flash chromatography over silica gel using (hexane/ethyl acetate, from 0 to 50 %) as the eluent to afford compound 3 as a colorless oil (2.7 g, 81%). ^{1}H NMR (400 MHz, $CDCl_3$) δ 7.76 (d, *J* = 7.5 Hz, 2H), 7.61 – 7.57 (m, 2H), 7.47-7.31 (m, 2H), 7.32-7.28 m, 2H), 5.28 (s, 1H), 4.40 (d, *J* = 6.9 Hz, 2H), 4.20 (t, *J* = 6.9 Hz, 1H), 3.58 (s, 3H), 3.29 (d, *J* = 6.4 Hz, 2H), 2.99 (t, *J* = 6.5 Hz, 2H), 1.75 (t, *J* = 6.6 Hz, 2H). ^{13}C NMR (100 MHz, $CDCl_3$) δ 156.58, 143.97, 141.32, 127.66, 127.02, 125.00, 119.95, 119.93, 77.37, 77.05, 76.73, 66.53, 61.88, 49.10, 47.30, 39.15, 27.09. See NMR spectra in **Figure S15**.

Synthesis of Compound 4. To a solution of compound 3 (2.7 g, 8.27 mmol) and $(Boc)_2O$ (3.61 g, 16.54 mmol) in DCM, DIPEA (1.73 mL, 9.93mmol) was added. The resultant solution was stirred at room temperature under N_2 for 18 h and washed with saturated NaCl, dried with Na_2SO_4 and concentrated to provide the crude product. The crude product was purified by column chromatography over silica gel using (hexane/ethyl acetate, from 0 to 35 %) as the eluent to afford Compound 4 (2.79 g, 79%).^{1}H NMR (400 MHz, $CDCl_3$) δ 7.76 (dd, *J* = 7.5, 0.9 Hz, 2H), 7.62 – 7.57 (m, 2H), 7.41– 7.38 (m, 2H), 7.33 – 7.29 (m, 2H), 5.19 (s, 1H), 4.39 (d, *J* = 7.0 Hz, 2H), 4.22 (t, *J* = 7.0 Hz, 1H), 3.68 (s, 3H), 3.51 (d, *J* = 6.9 Hz, 2H), 3.31 – 3.19 (m, 2H), 1.85 – 1.76 (m, 2H), 1.50 (s, 9H). ^{13}C NMR (100 MHz, $CDCl_3$) δ 171.10, 156.40, 144.01, 141.31, 127.62, 127.00, 125.07, 119.92, 81.55, 66.60, 62.18, 60.36, 47.30, 45.78, 38.08, 28.29, 27.36, 21.01, 14.18. See NMR spectra in **Figure S15**.

Synthesis of Compound 5. To a solution of compound 4 (1 g, 2.34 mmol) in 20% piperidine in DMF. The mixture solution was stirred at room temperature under N_2 for 30 min. The organic solvent was removed by a high vacuum oil pump to afford crude product. To a solution of Tetrazine acid (481.56 mg, 2.23 mmol) and HATU (936.09 mg, 2.46 mmol)

in DMF was added DIPEA (606.06 mg, 4.69 mmol) and the solution was stirred under at RT for 30 min. After 30 min, the above crude product was injected into the reaction. The resultant solution was stirred at room temperature under N_2 for overnight and washed with saturated NaCl, and organic phases were extracted with ethyl acetate, dried with Na_2SO_4 and concentrated to provide the crude product. The crude product was purified by column chromatography over silica gel using (hexane/ethyl acetate, from 0 to 35 %) as the eluent to afford Compound 5 (641mg, 68%). ^{1}H NMR (400 MHz, $CDCl_3$) δ 8.65 (d, *J* = 8.5 Hz, 2H), 8.04 (d, *J* = 8.5 Hz, 2H), 3.70 (s, 3H), 3.62 (t, *J* = 6.1 Hz, 2H), 3.52 (q, *J* = 6.0 Hz, 2H), 3.10 (s, 3H), 1.94 – 1.88 (m, 2H), 1.50 (s, 9H). ^{13}C NMR (100 MHz, $CDCl_3$) δ 167.49, 166.27, 163.61, 156.74, 138.13, 134.26, 128.03, 127.87, 81.79, 62.19, 45.43, 36.71, 28.30, 26.80, 21.18. See NMR spectra in **Figure S15**.

Synthesis of Compound MTZ HCl salt. Compound 5 (200 mg, 1.59 mmol) in DCM was treated with 4M HCl in 1,4-dioxane (1.24 mL). The mixture was stirred at rt for overnight, and cold Et_2O was added until no more precipitate formed. If there are starting material remains, concentrate the solution and repeat this step. The precipitate was collected and washed with cold Et_2O to afford MTZ HCl salt as pink solid (167 mg, 99%). These pink solids were dissolved in di-H_2O and lyophilized. ^{1}H NMR (400 MHz, D_2O) δ 8.41 (d, *J* = 8.6 Hz, 2H), 7.89 (d, *J* = 8.5 Hz, 2H), 3.76 (s, 3H), 3.45 (t, *J* = 6.8 Hz, 2H), 3.32 – 3.24 (m, 2H), 3.00 (s, 3H), 2.02 – 1.89 (m, 2H). ^{13}C NMR (100 MHz, D_2O) δ 170.18, 167.58, 163.62, 137.07, 134.49, 128.17, 128.01, 61.35, 46.76, 37.10, 23.73, 20.13. ESI HRMS, calculated for $C_{14}H_{18}ClN_6O_2^-$ $[M\text{-}H]^-$ 337.1185, found 337.1180. See NMR spectra in **Figure S15**.

Supplementary Tables

Table S1. Summary of optimization of TCO -PEG6-NHS and 3’-SL.

TCO-PEG6-NHS linker (eq)	Average Loading #	3’-SL (eq)	Average Loading #
10	6	10	1
25	15	25	5
50	29	50	9
100	46	100	21
		200	21
		500	20

Supplementary Figures and Legends

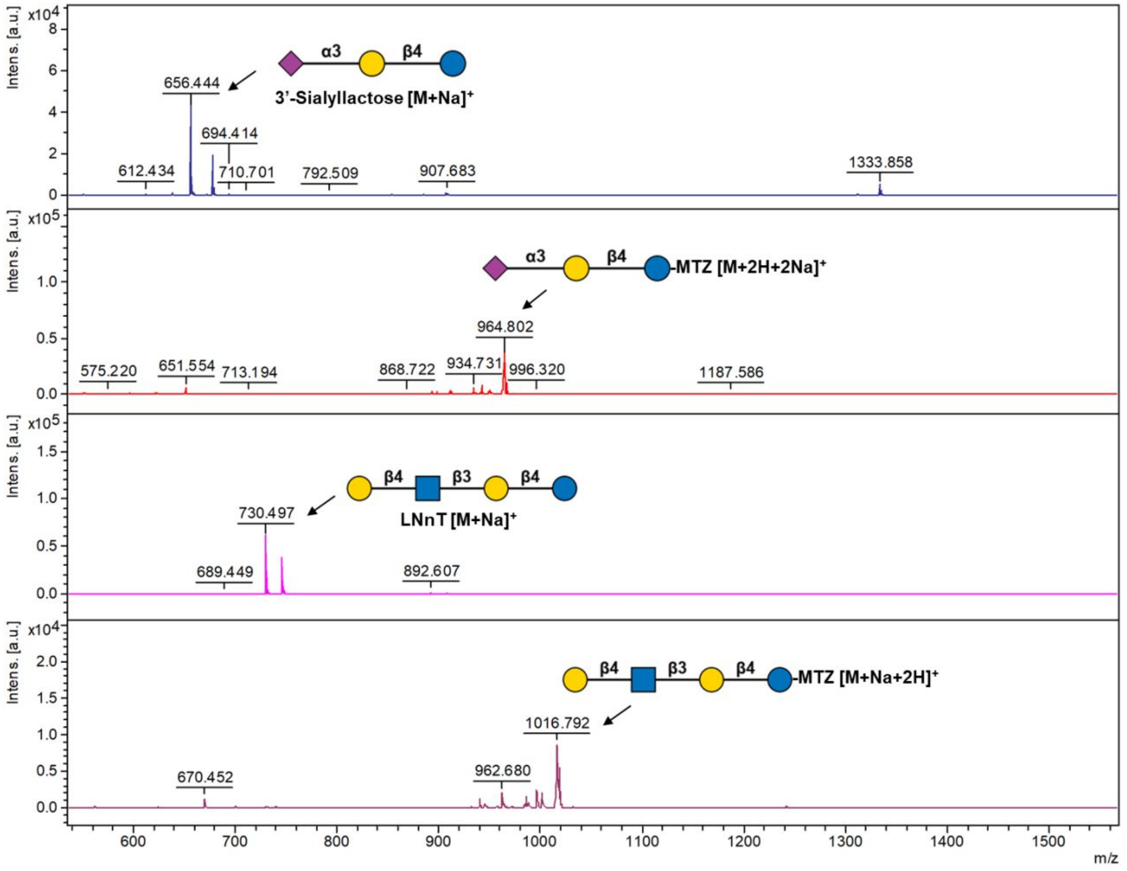

Figure S1. MALDI-TOF Mass spectrometry of 3'-SL-MTZ and LNnT-MTZ.

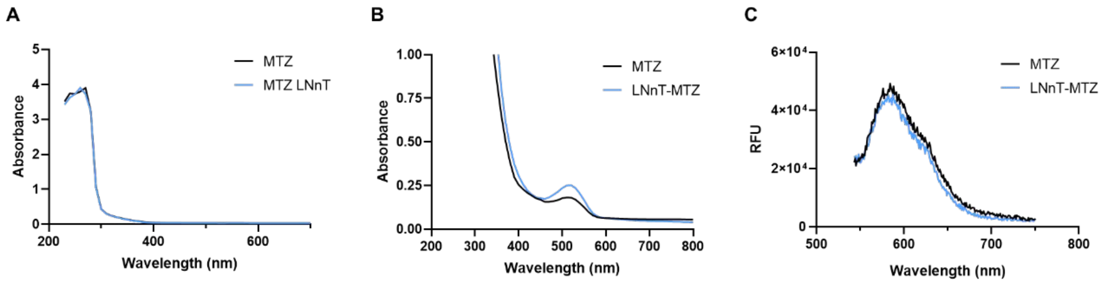

Figure S2. UV-vis absorption spectra of A) 31.25 µM MTZ and 31.25 µM LNnT-MTZ showing strong absorption at 270 nm, and B) 2 mM MTZ and 3.17 mM LNnT-MTZ revealing weaker absorption at 520 nm. Fluorescence spectra of C) 62.5 µM MTZ and 62.5 µM LNnT-MTZ highlight fluorescence with excitation at 520 nm and emission at 580 nm.

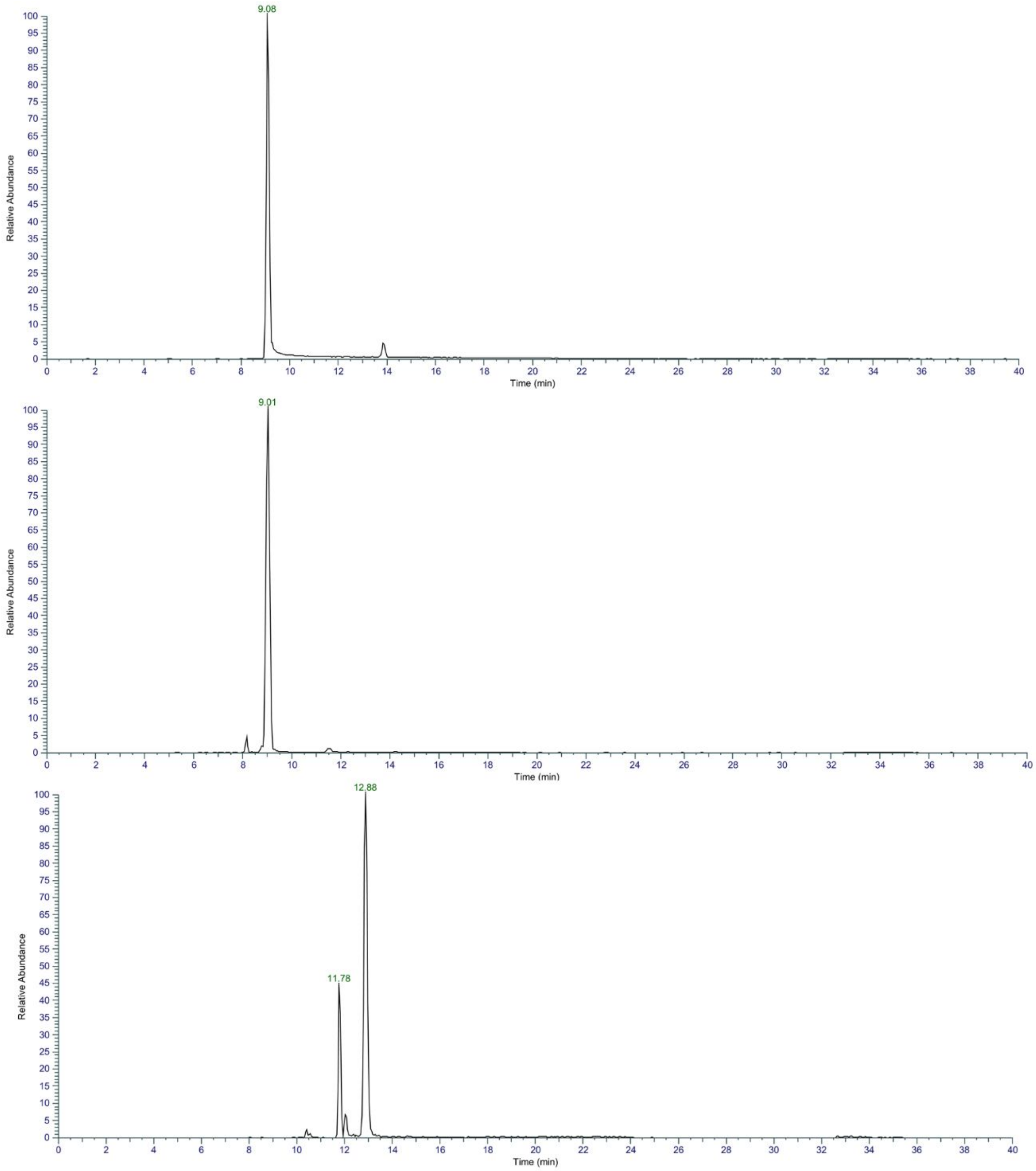

Figure S3. ESI-LC-MS chromatograms of starting material H5 (top), B4 (middle) and A1 (bottom). All ESI raw files are available at the Dataverse link provided within the manuscript.

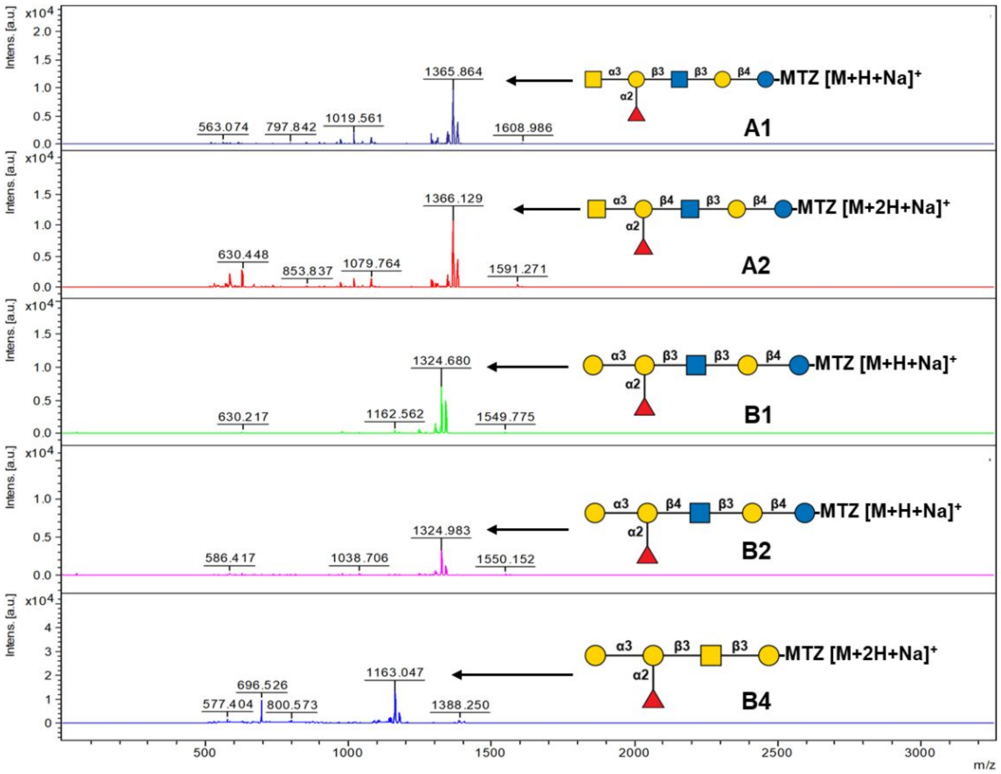

Intens. [a.u.]
x10^4
1365.864
563.074
797.842
1019.561
1608.986
α3 β3 β3 β4
α2
-MTZ [M+H+Na]+
A1
1366.129
630.448
853.837
1079.764
1591.271
α3 β4 β3 β4
α2
-MTZ [M+2H+Na]+
A2
1324.680
630.217
1162.562
1549.775
α3 β3 β3 β4
α2
-MTZ [M+H+Na]+
B1
1324.983
586.417
1038.706
1550.152
α3 β4 β3 β4
α2
-MTZ [M+H+Na]+
B2
1163.047
696.526
577.404
800.573
1388.250
α3 β3 β3
α2
-MTZ [M+2H+Na]+
B4
500
1000
1500
2000
2500
3000
m/z

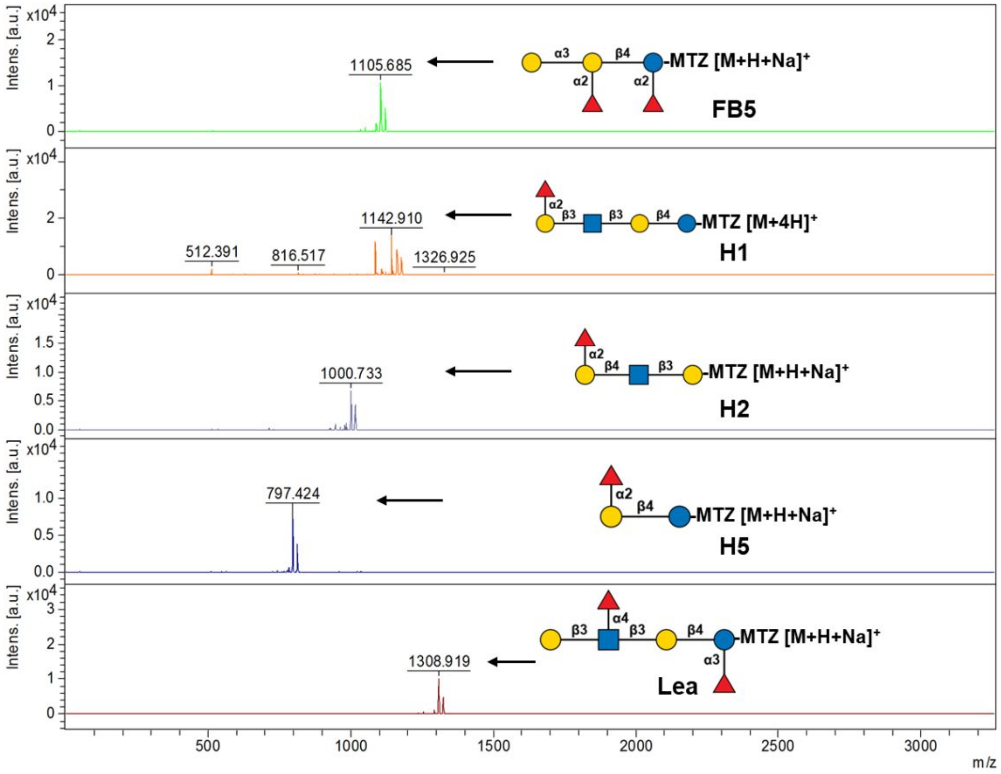

Intens. [a.u.]
x10^4
1105.685
α3 β4
α2 α2
-MTZ [M+H+Na]+
FB5
1142.910
512.391
816.517
1326.925
α2 β3 β3 β4
-MTZ [M+4H]+
H1
1000.733
α2 β4 β3
-MTZ [M+H+Na]+
H2
797.424
α2 β4
-MTZ [M+H+Na]+
H5
1308.919
α4 β3 β3 β4
α3
-MTZ [M+H+Na]+
Lea
500
1000
1500
2000
2500
3000
m/z

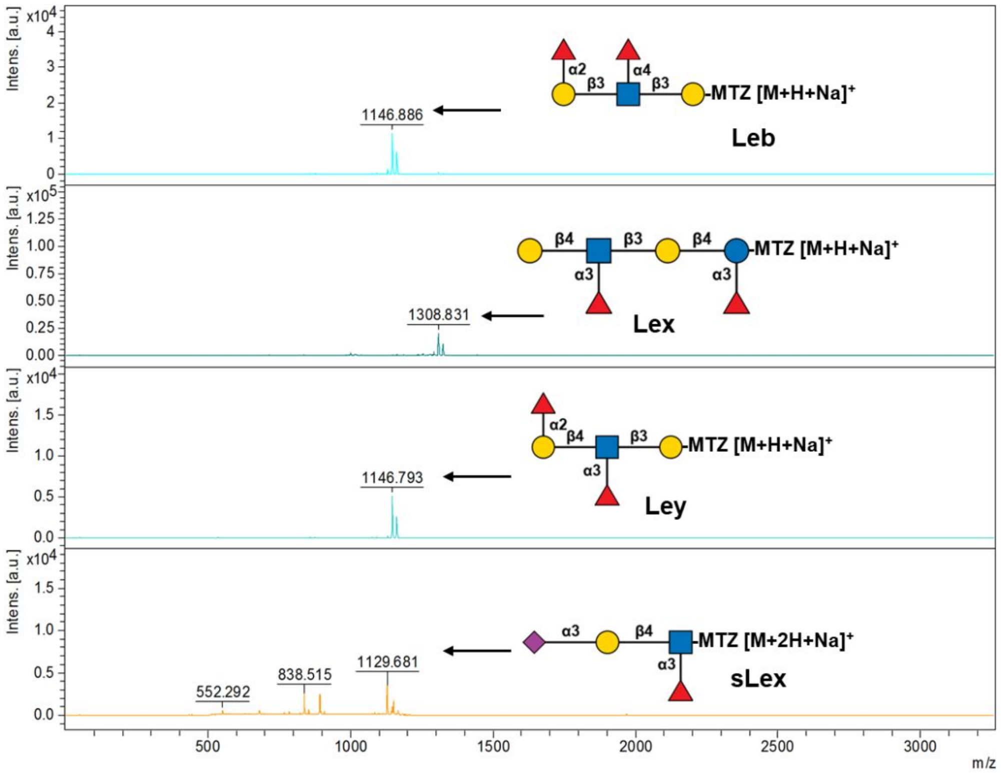

Figure S4. MALDI-TOF Mass spectrometry of 14 MTZ-linked blood group glycans.

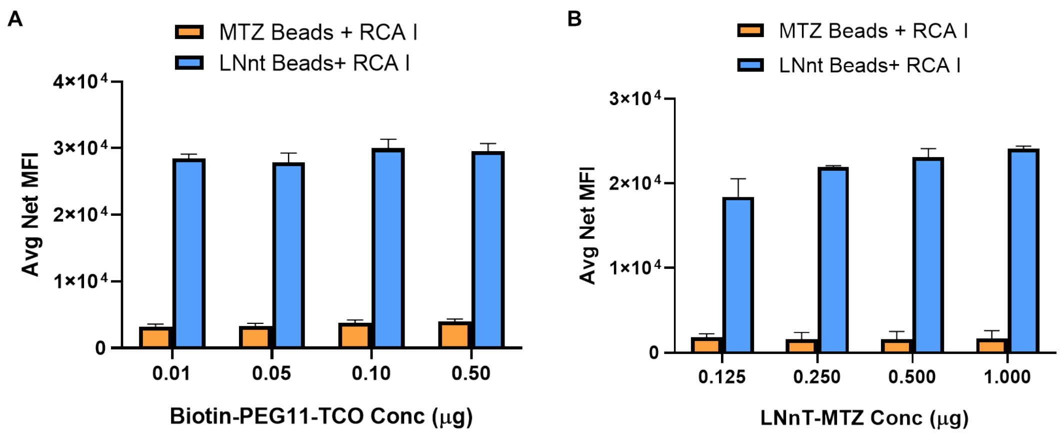

Figure S5. Optimization of glycan beads preparation. Evaluation of various concentration of A) Biotin-PEG11-TCO linker and B) LNnT-MTZ. The y-axis represents the average net (background subtracted) median fluorescence intensity (MFI). The data are presented as an average net MFI of two replicates; each experiment was repeated two times. Error bars = +/- 1 SD.

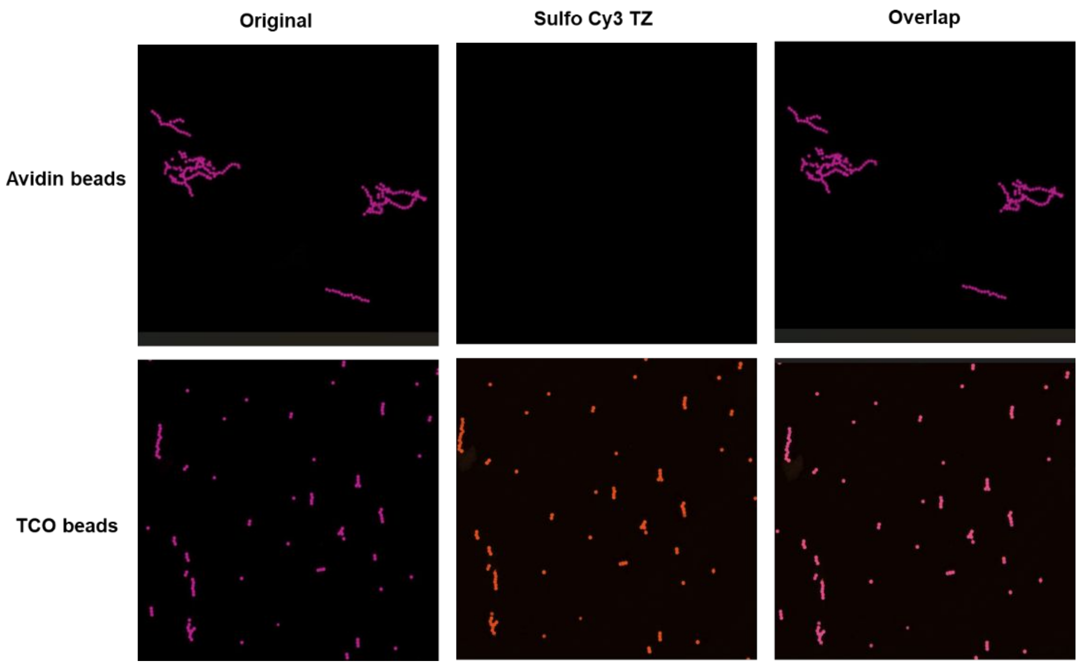

Figure S6. The image of TCO beads stain with sulfo-Cy3-methyltetrazine dye.

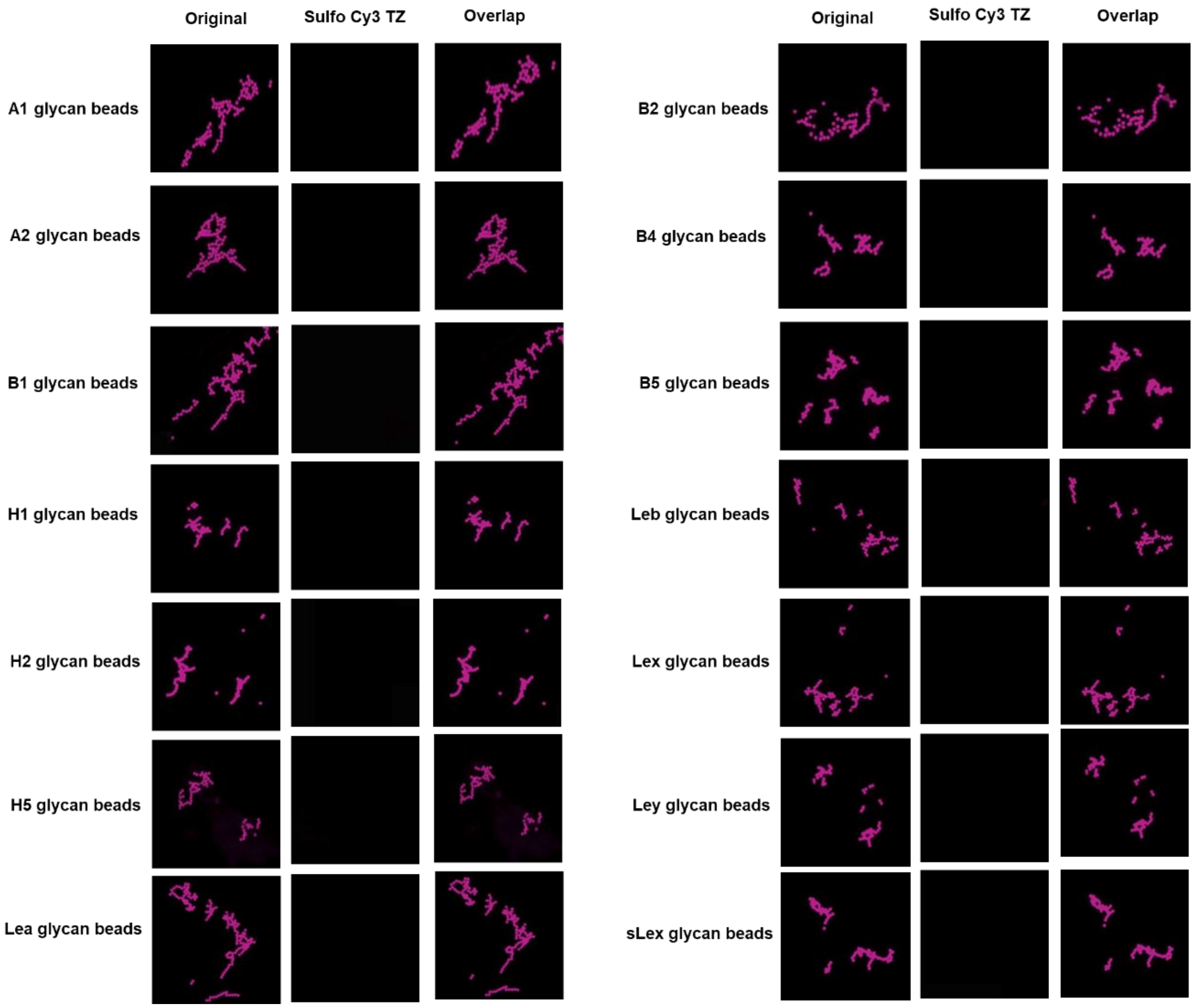

Figure S7. The image of glycan beads stain with sulfo-Cy3-methyltetrazine dye.

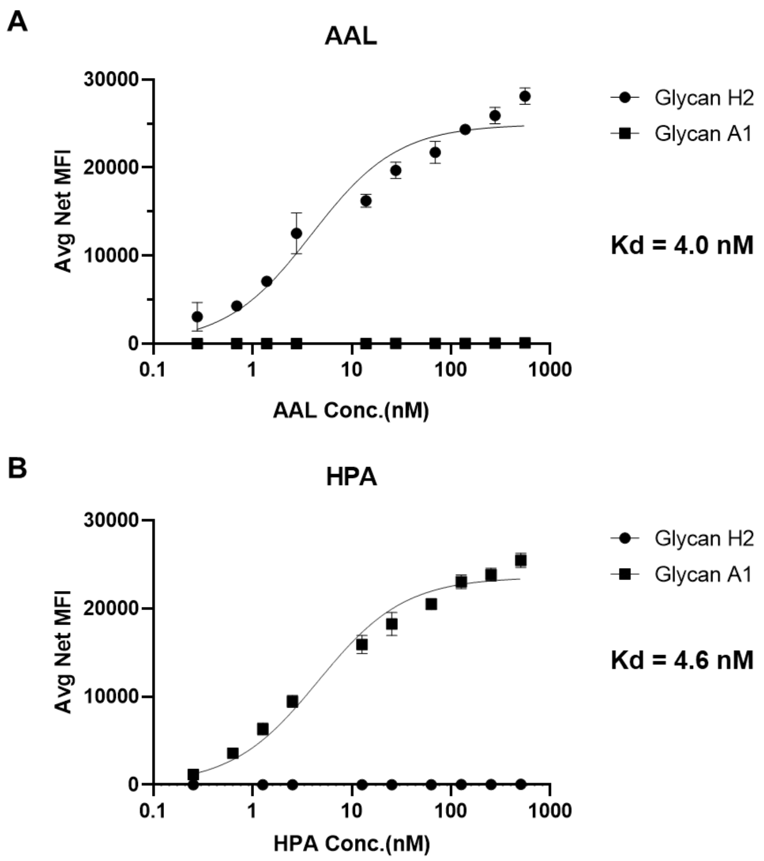

Figure S8. Affinity measurement using varying concentrations of Lectin A) AAL and B) HPA incubated with Glycan H2 and Glycan A1 Luminex beads. The binding affinity (Kd) is defined as the concentration of lectin required to achieve 50% of the maximum binding signal. The y-axis represents the average net (background subtracted) median fluorescence intensity (MFI). The data are presented as an average net MFI of two replicates; each experiment was repeated two times. Error bars = +/- 1 SD.

Figure S9

Sample 1 IgG (blue), IgM (green), IgA (orange)

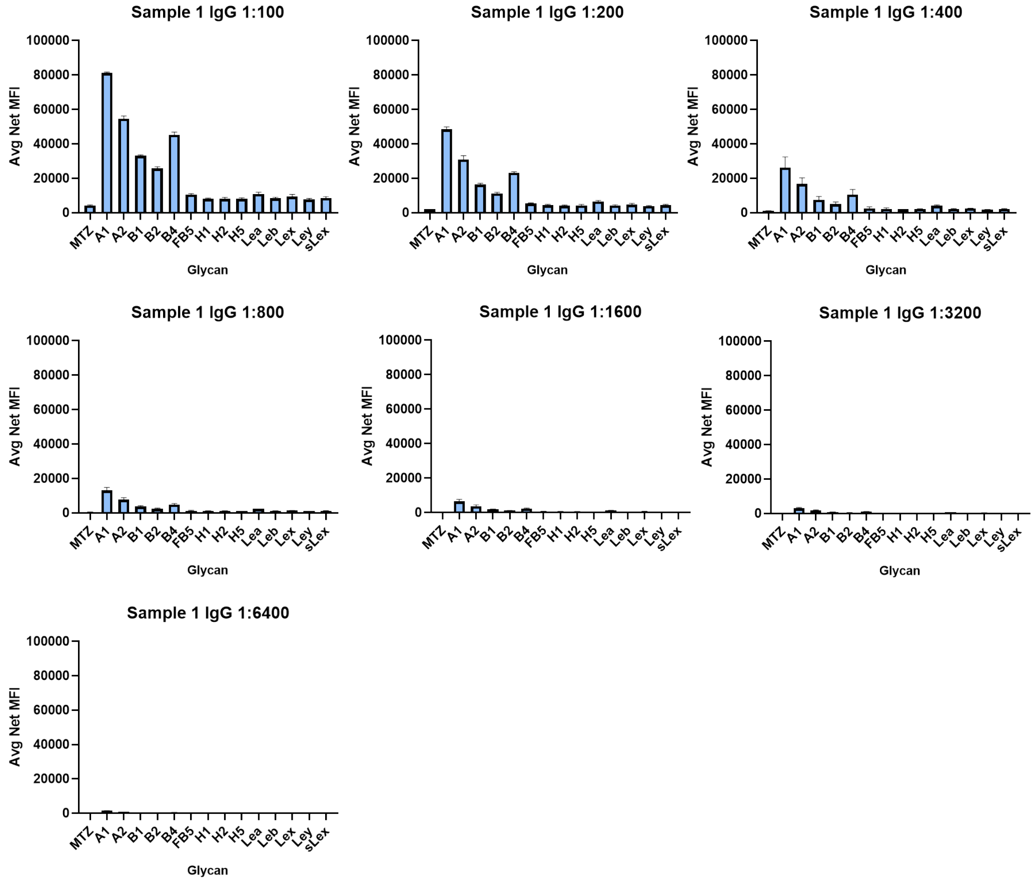

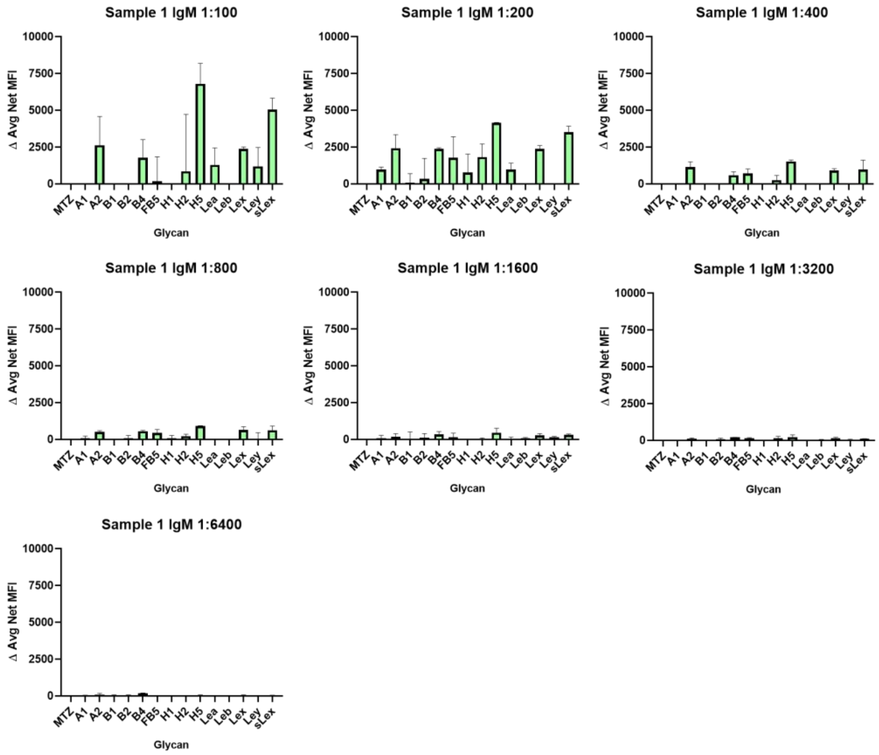
Sample 1 IgM 1:100
Sample 1 IgM 1:200
Sample 1 IgM 1:400
Sample 1 IgM 1:800
Sample 1 IgM 1:1600
Sample 1 IgM 1:3200
Sample 1 IgM 1:6400
Δ Avg Net MFI
Glycan
MTZ A1 A2 B1 B2 B4 FB5 H1 H2 H5 Lea Leb Lex Ley sLex
0 2500 5000 7500 10000

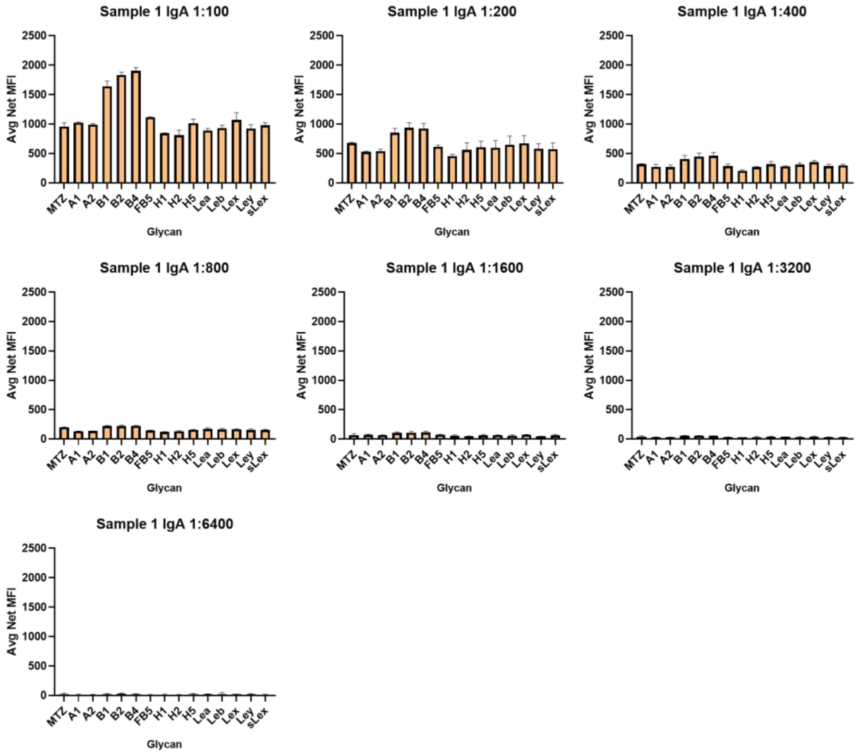
Sample 1 IgA 1:100
Sample 1 IgA 1:200
Sample 1 IgA 1:400
Sample 1 IgA 1:800
Sample 1 IgA 1:1600
Sample 1 IgA 1:3200
Sample 1 IgA 1:6400
Avg Net MFI
Glycan
MTZ A1 A2 B1 B2 B4 FB5 H1 H2 H5 Lea Leb Lex Ley sLex
0 500 1000 1500 2000 2500

Sample 2 IgG, IgM, IgA

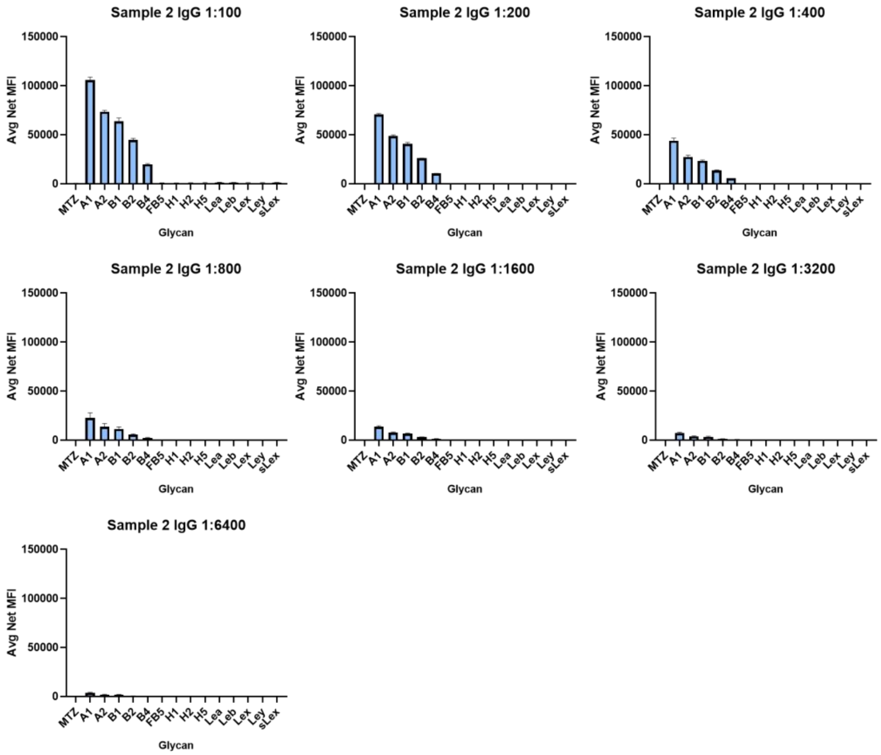

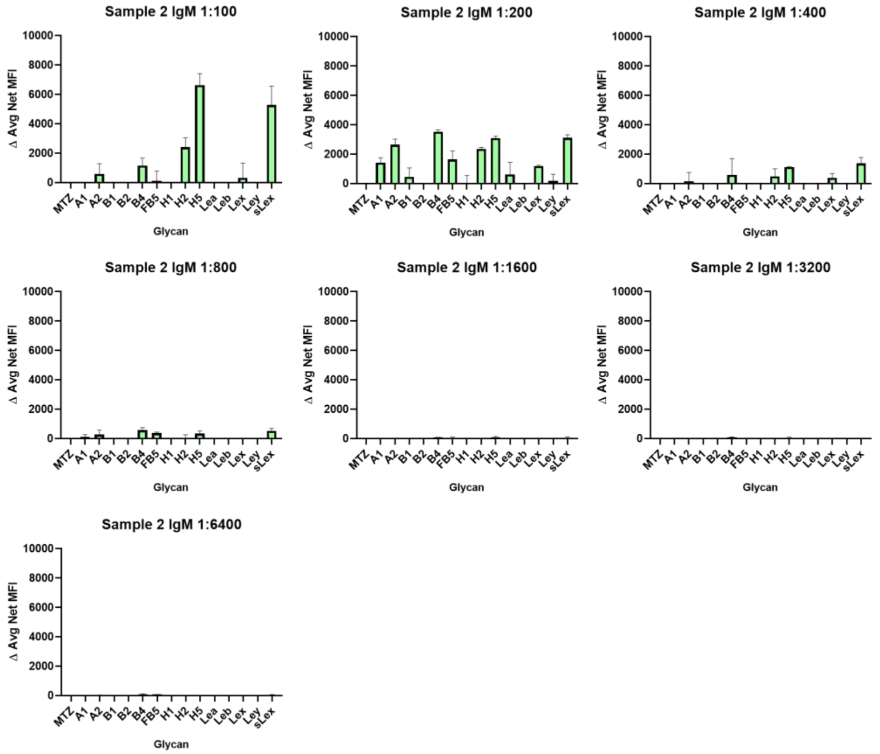

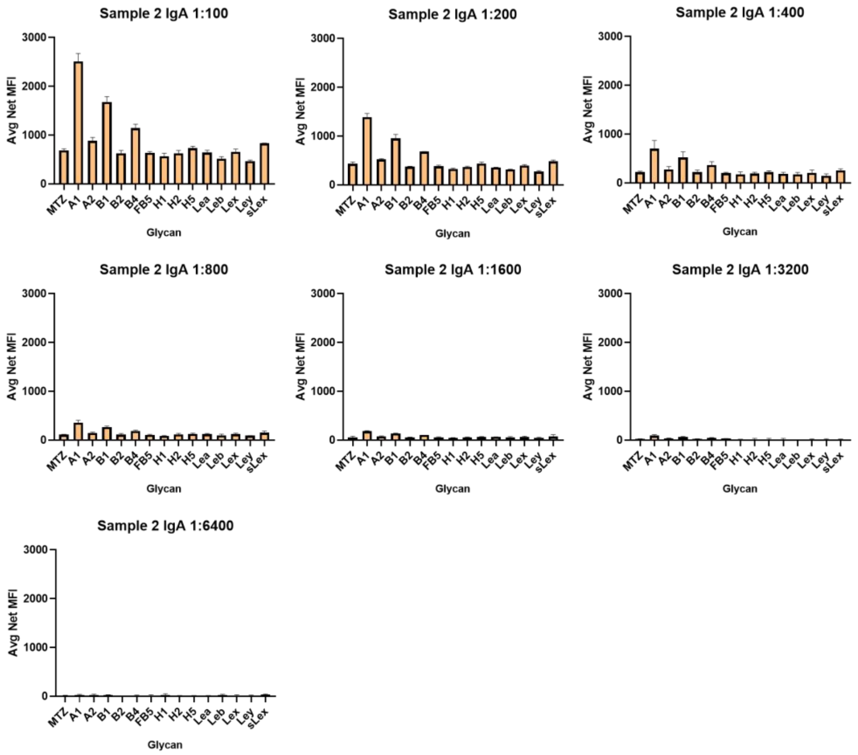

Sample 3 IgG, IgM, IgA

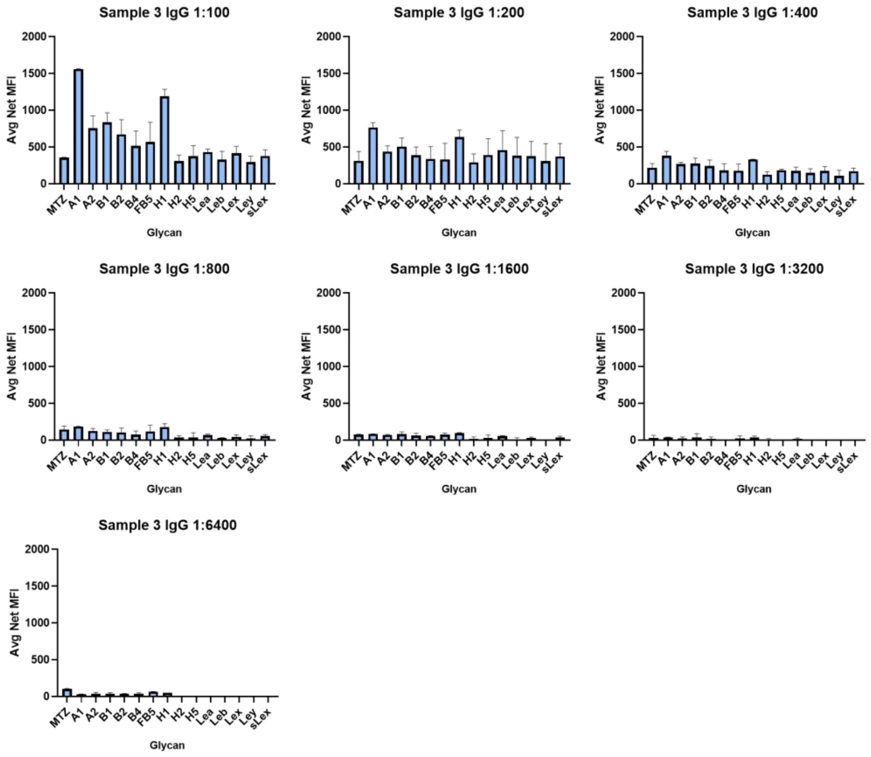

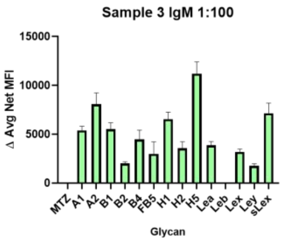
Sample 3 IgM 1:100
Δ Avg Net MFI
Glycan

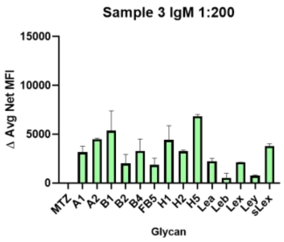
Sample 3 IgM 1:200
Δ Avg Net MFI
Glycan

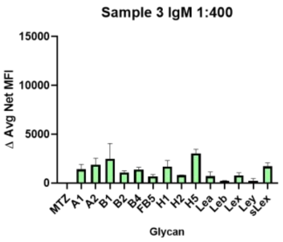
Sample 3 IgM 1:400
Δ Avg Net MFI
Glycan

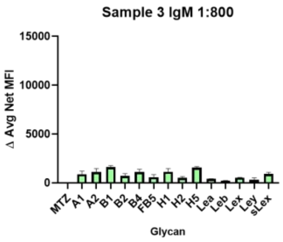
Sample 3 IgM 1:800
Δ Avg Net MFI
Glycan

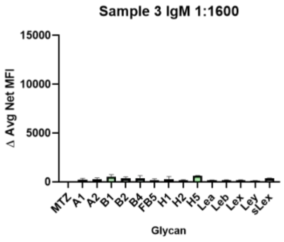
Sample 3 IgM 1:1600
Δ Avg Net MFI
Glycan

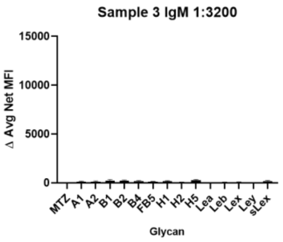
Sample 3 IgM 1:3200
Δ Avg Net MFI
Glycan

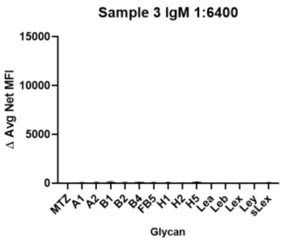
Sample 3 IgM 1:6400
Δ Avg Net MFI
Glycan

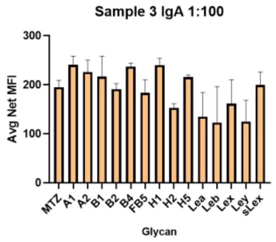
Sample 3 IgA 1:100
Avg Net MFI
Glycan

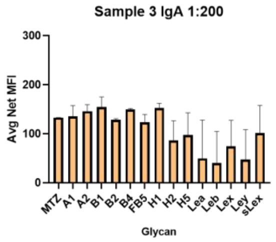
Sample 3 IgA 1:200
Avg Net MFI
Glycan

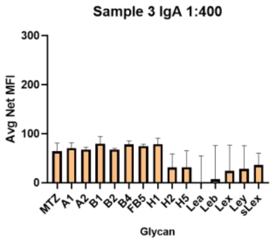
Sample 3 IgA 1:400
Avg Net MFI
Glycan

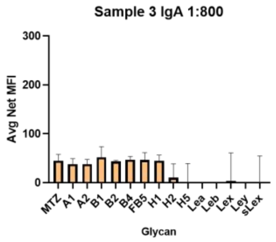
Sample 3 IgA 1:800
Avg Net MFI
Glycan

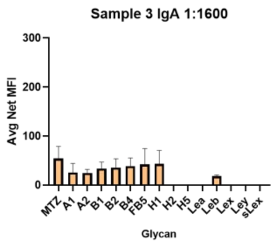
Sample 3 IgA 1:1600
Avg Net MFI
Glycan

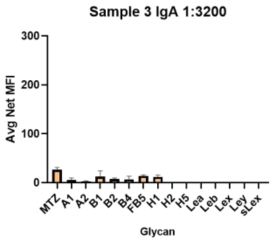
Sample 3 IgA 1:3200
Avg Net MFI
Glycan

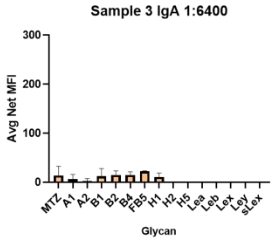
Sample 3 IgA 1:6400
Avg Net MFI
Glycan

Sample 4 IgG, IgM, IgA

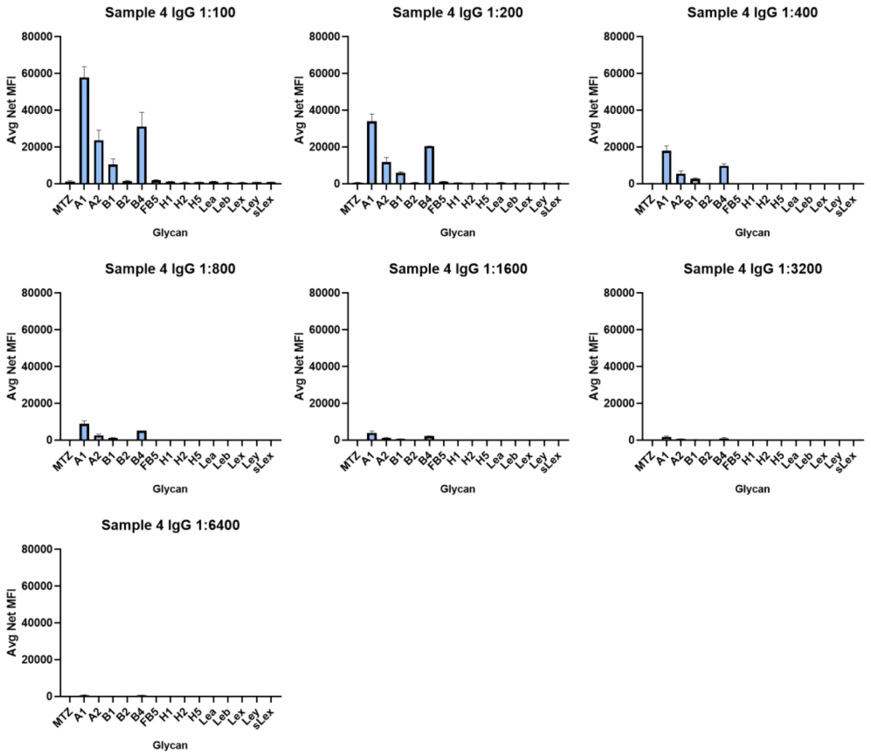

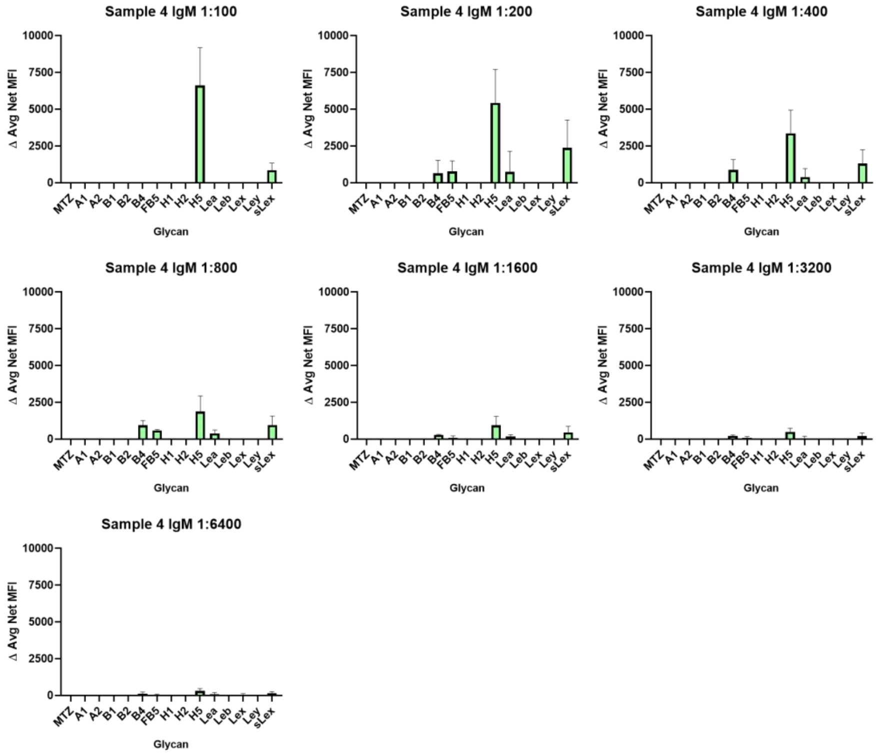

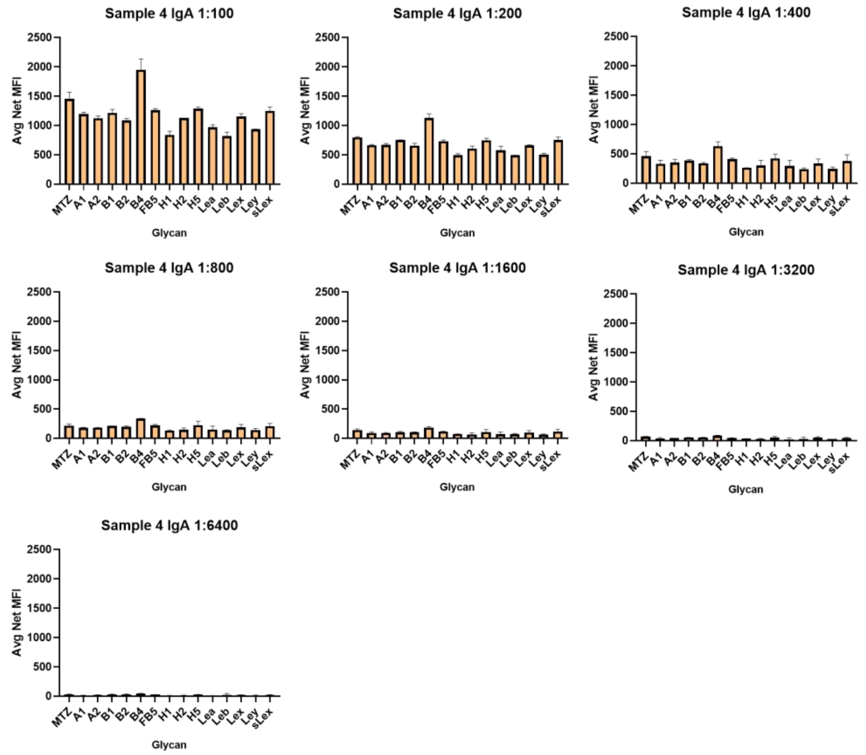

Sample 5 IgG, IgM, IgA

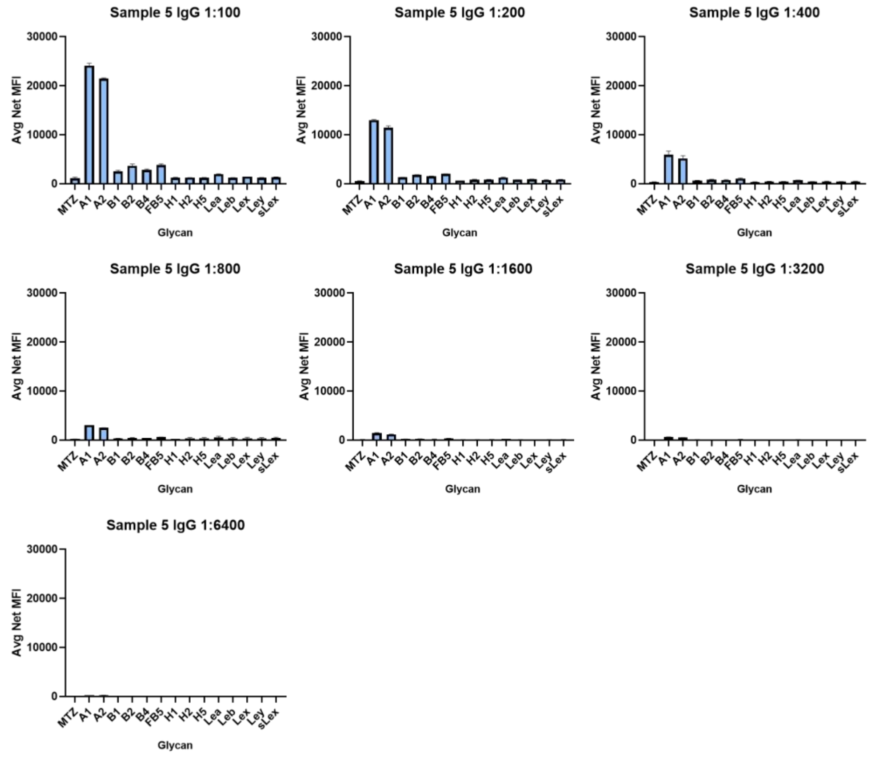

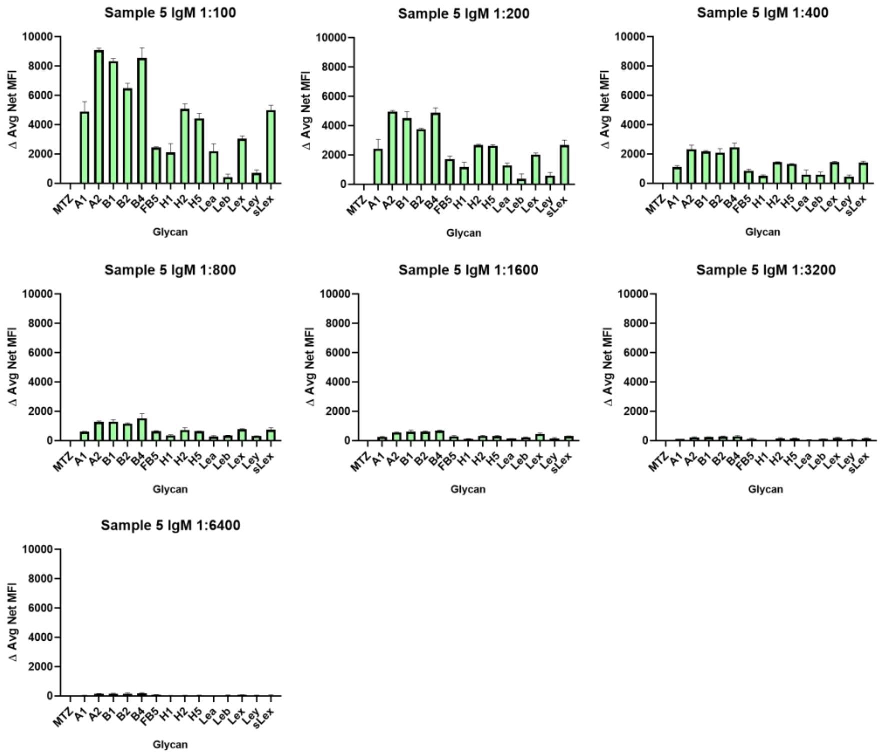
Sample 5 IgM 1:100
Sample 5 IgM 1:200
Sample 5 IgM 1:400
Sample 5 IgM 1:800
Sample 5 IgM 1:1600
Sample 5 IgM 1:3200
Sample 5 IgM 1:6400
Δ Avg Net MFI
0
2000
4000
6000
8000
10000
MTZ
A1
A2
B1
B2
B4
FB5
H1
H2
H5
Lea
Leb
Lex
Ley
sLex
Glycan

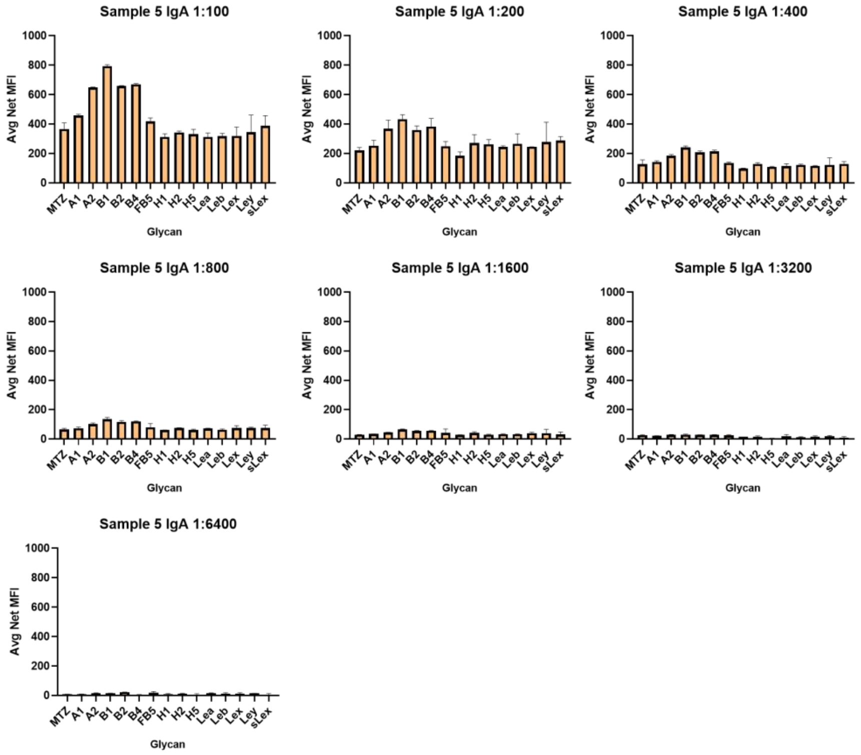
Sample 5 IgA 1:100
Sample 5 IgA 1:200
Sample 5 IgA 1:400
Sample 5 IgA 1:800
Sample 5 IgA 1:1600
Sample 5 IgA 1:3200
Sample 5 IgA 1:6400
Avg Net MFI
0
200
400
600
800
1000
MTZ
A1
A2
B1
B2
B4
FB5
H1
H2
H5
Lea
Leb
Lex
Ley
sLex
Glycan

Sample 6 IgG, IgM, IgA

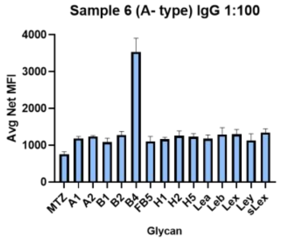

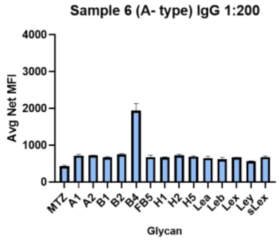

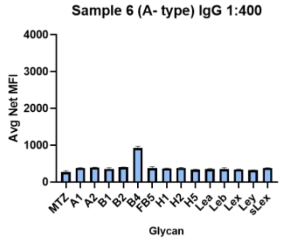

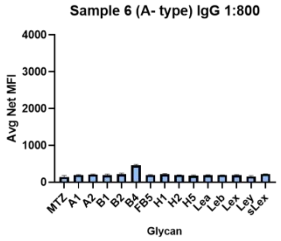

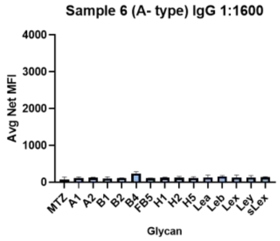

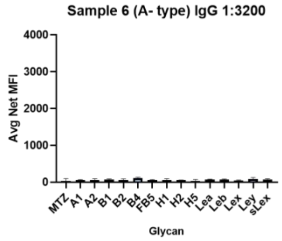

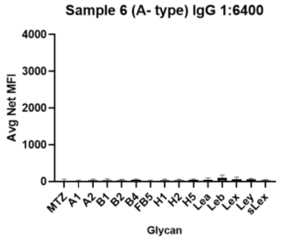

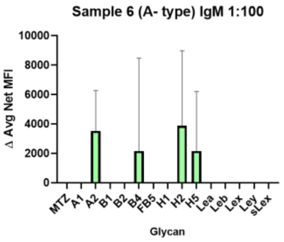

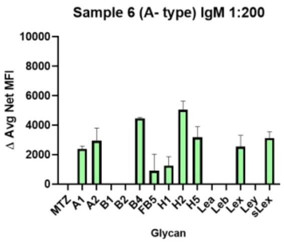

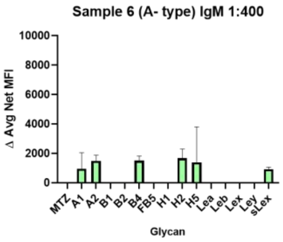

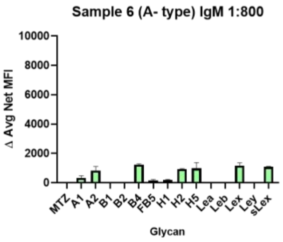

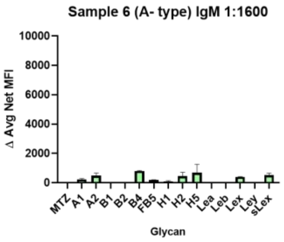

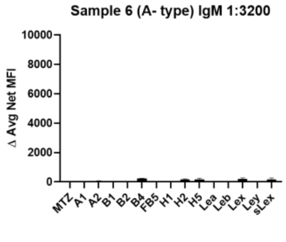

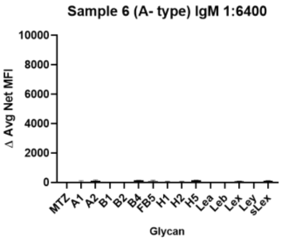

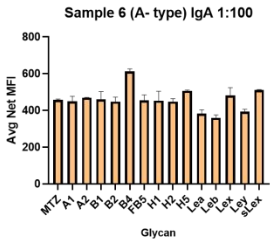

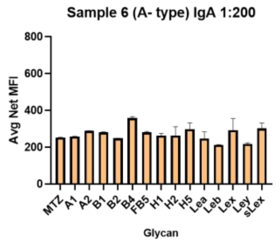

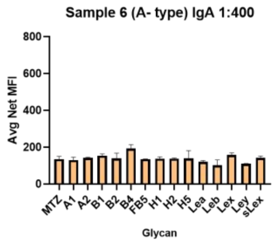

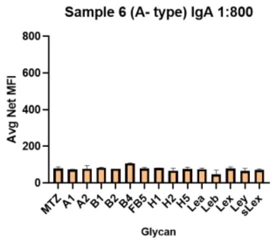

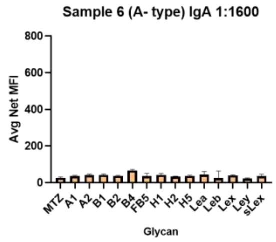

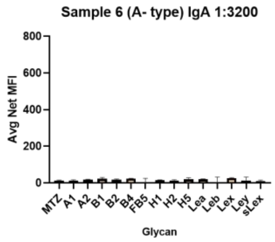

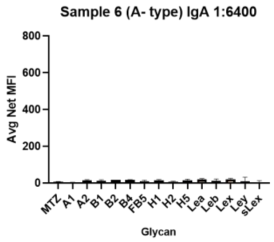

Sample 7 IgG, IgM, IgA

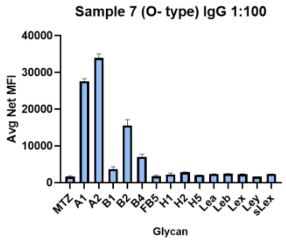

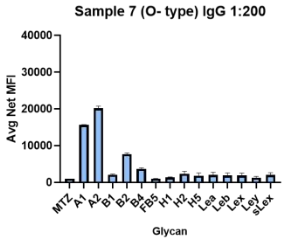

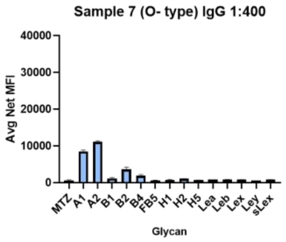

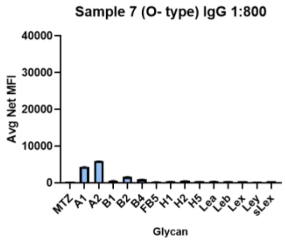

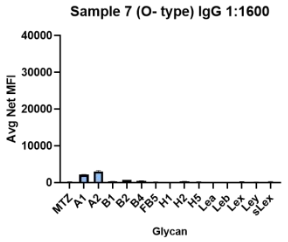

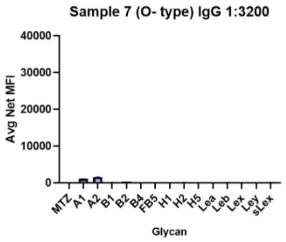

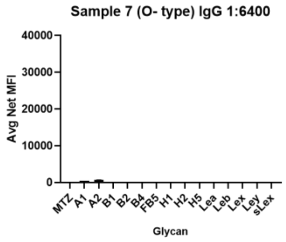

Sample 7 (O- type) IgM 1:100

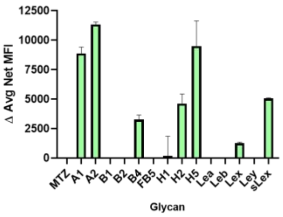

Sample 7 (O- type) IgM 1:200

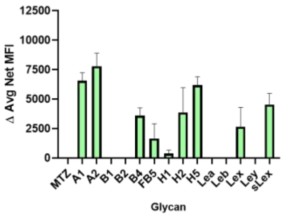

Sample 7 (O- type) IgM 1:400

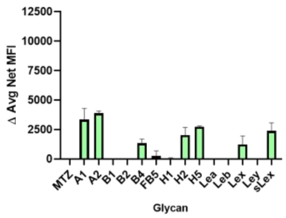

Sample 7 (O- type) IgM 1:800

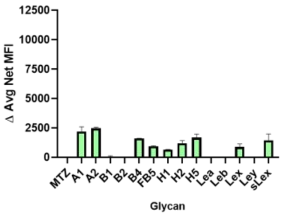

Sample 7 (O- type) IgM 1:1600

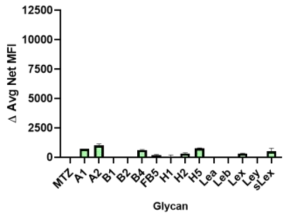

Sample 7 (O- type) IgM 1:3200

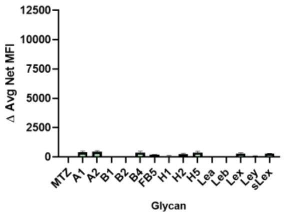

Sample 7 (O- type) IgM 1:6400

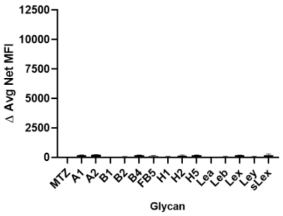

Sample 7 (O- type) IgA 1:100

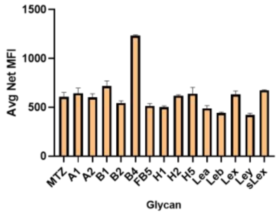

Sample 7 (O- type) IgA 1:200

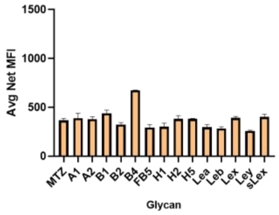

Sample 7 (O- type) IgA 1:400

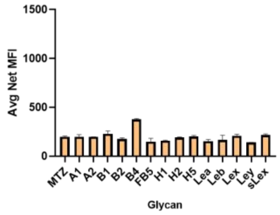

Sample 7 (O- type) IgA 1:800

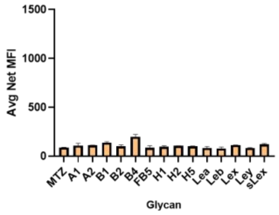

Sample 7 (O- type) IgA 1:1600

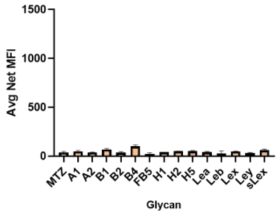

Sample 7 (O- type) IgA 1:3200

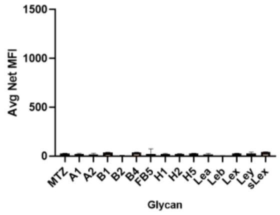

Sample 7 9O- type) IgA 1:6400

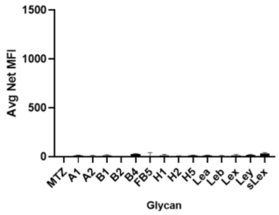

Sample 8 IgG, IgM, IgA

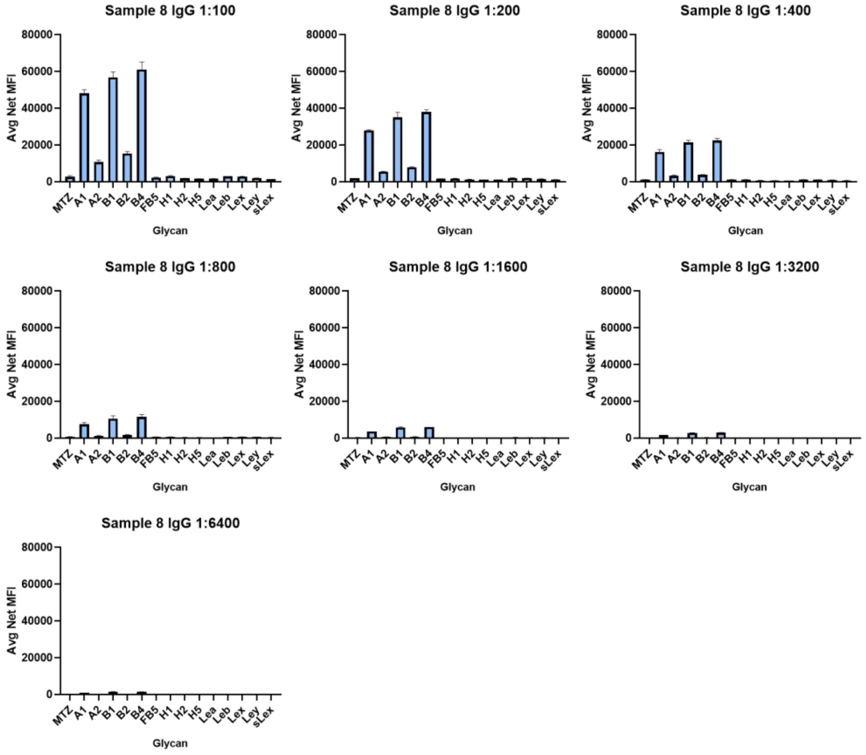

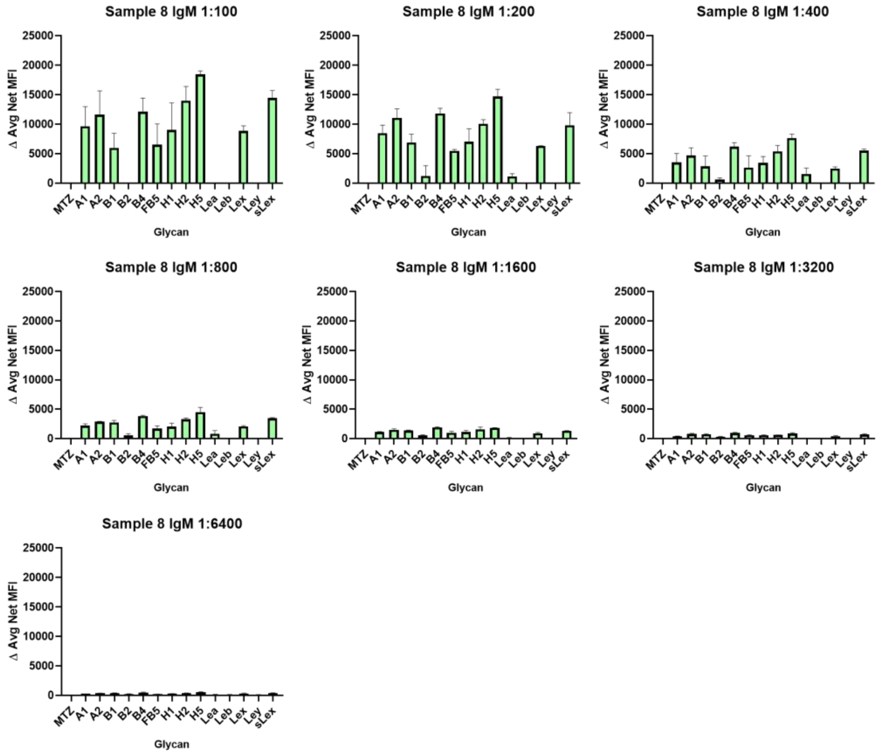

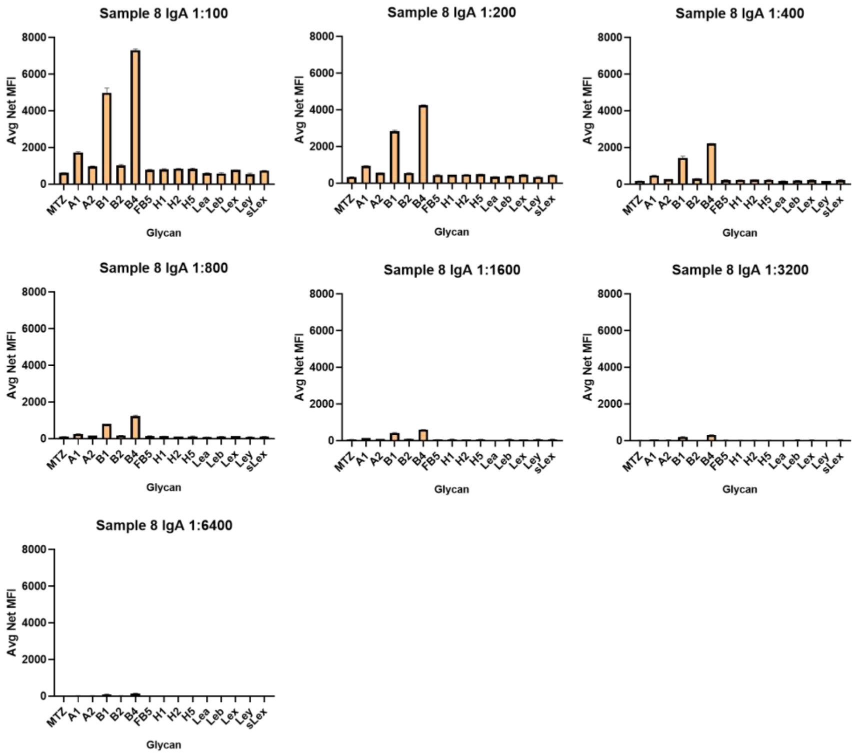

Sample 9 IgG, IgM, IgA

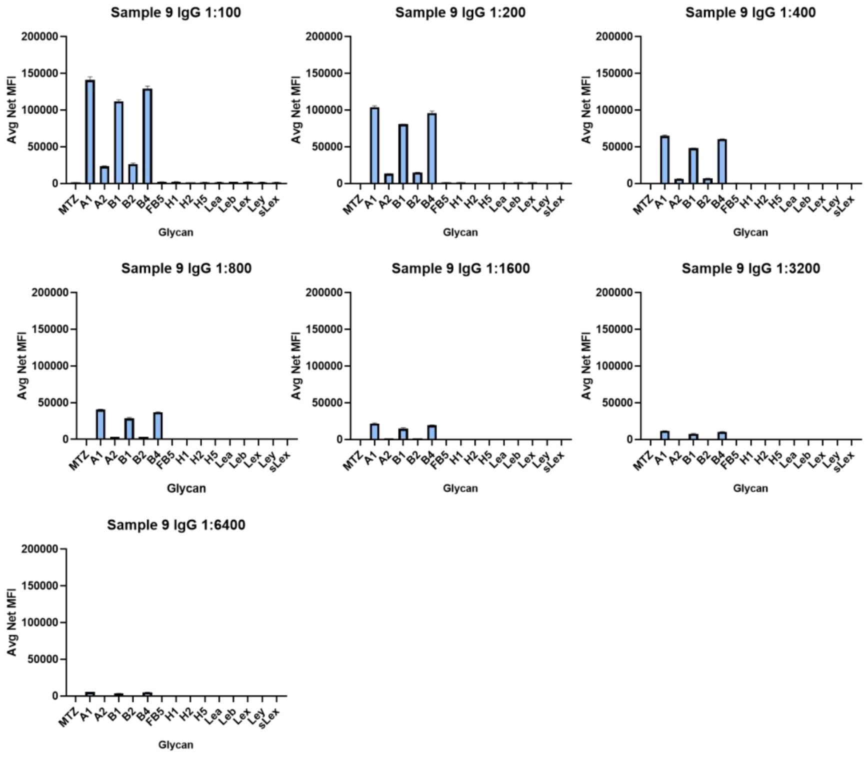

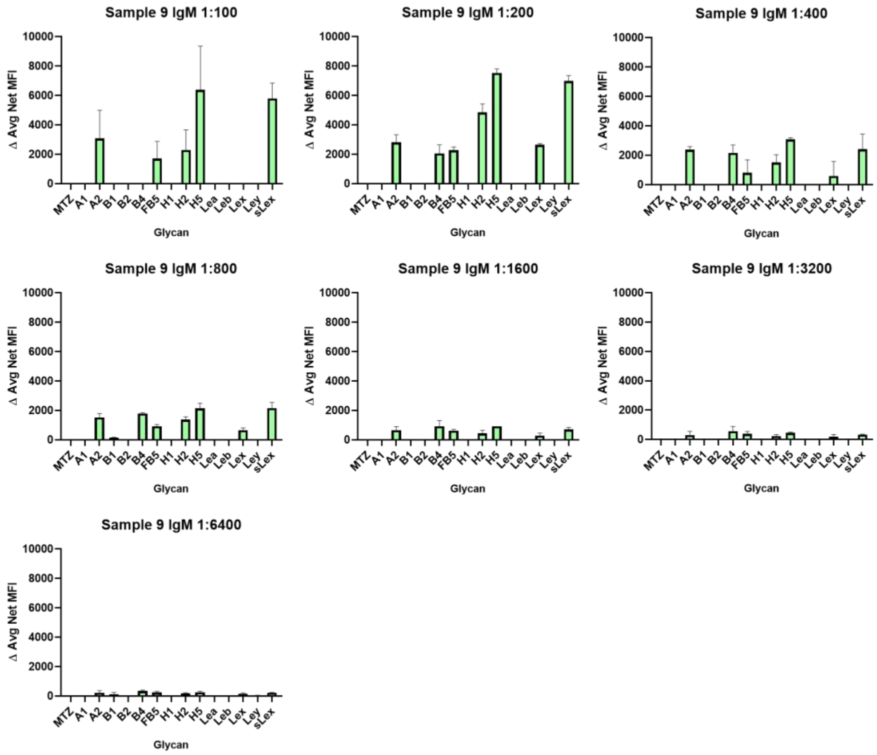
Sample 9 IgM 1:100
Sample 9 IgM 1:200
Sample 9 IgM 1:400
Sample 9 IgM 1:800
Sample 9 IgM 1:1600
Sample 9 IgM 1:3200
Sample 9 IgM 1:6400
Δ Avg Net MFI
10000
8000
6000
4000
2000
0
MTZ
A1
A2
B1
B2
B4
FB5
H1
H2
H5
Lea
Leb
Lex
Ley
sLex
Glycan

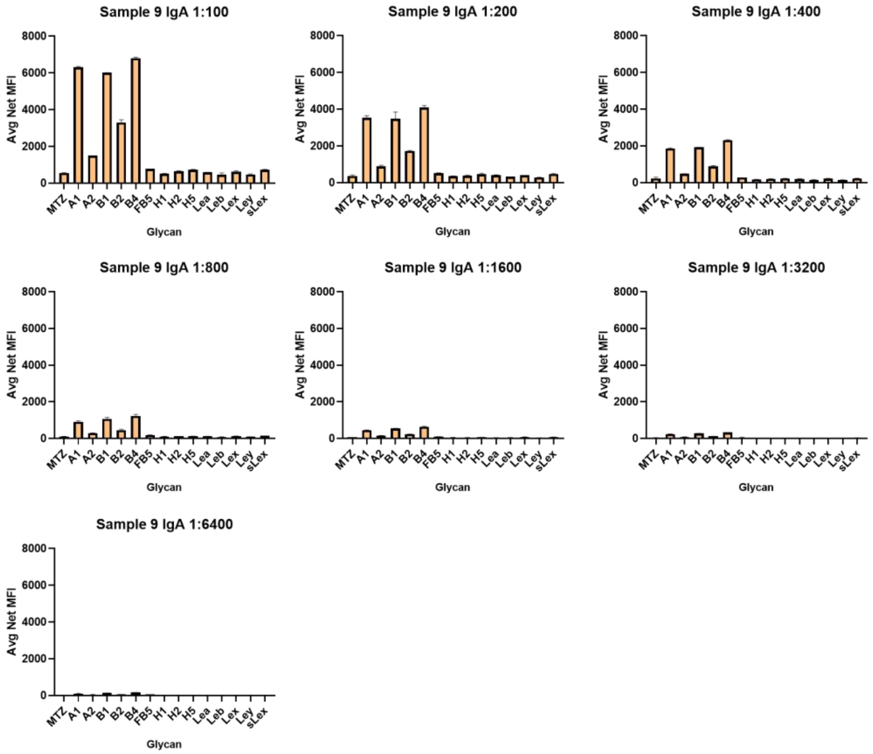
Sample 9 IgA 1:100
Sample 9 IgA 1:200
Sample 9 IgA 1:400
Sample 9 IgA 1:800
Sample 9 IgA 1:1600
Sample 9 IgA 1:3200
Sample 9 IgA 1:6400
Avg Net MFI
8000
6000
4000
2000
0
MTZ
A1
A2
B1
B2
B4
FB5
H1
H2
H5
Lea
Leb
Lex
Ley
sLex
Glycan

Sample 10 IgG, IgM, IgA

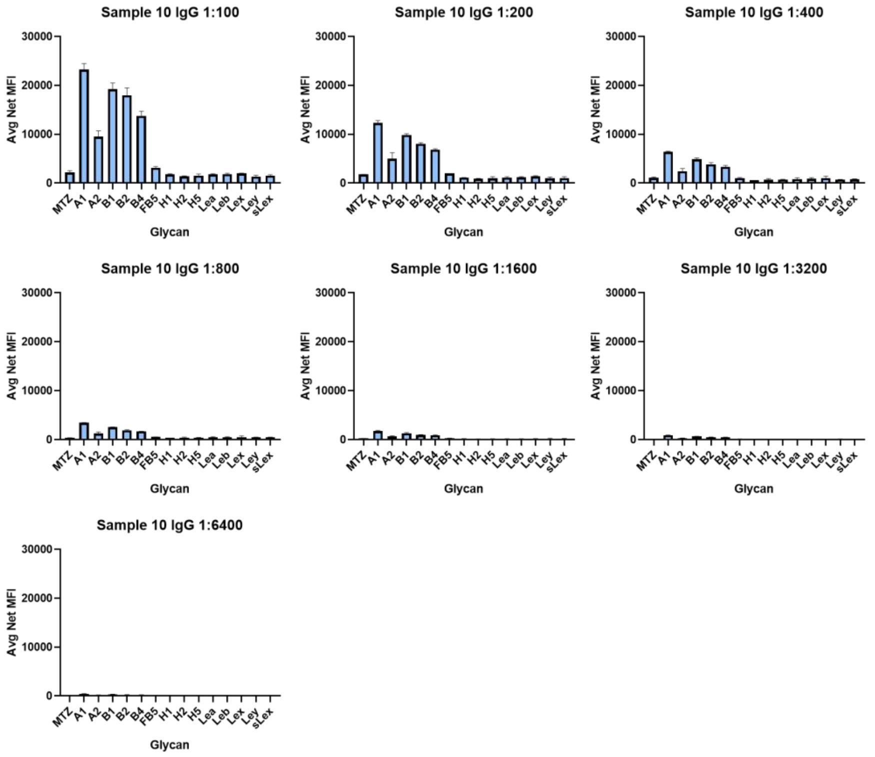

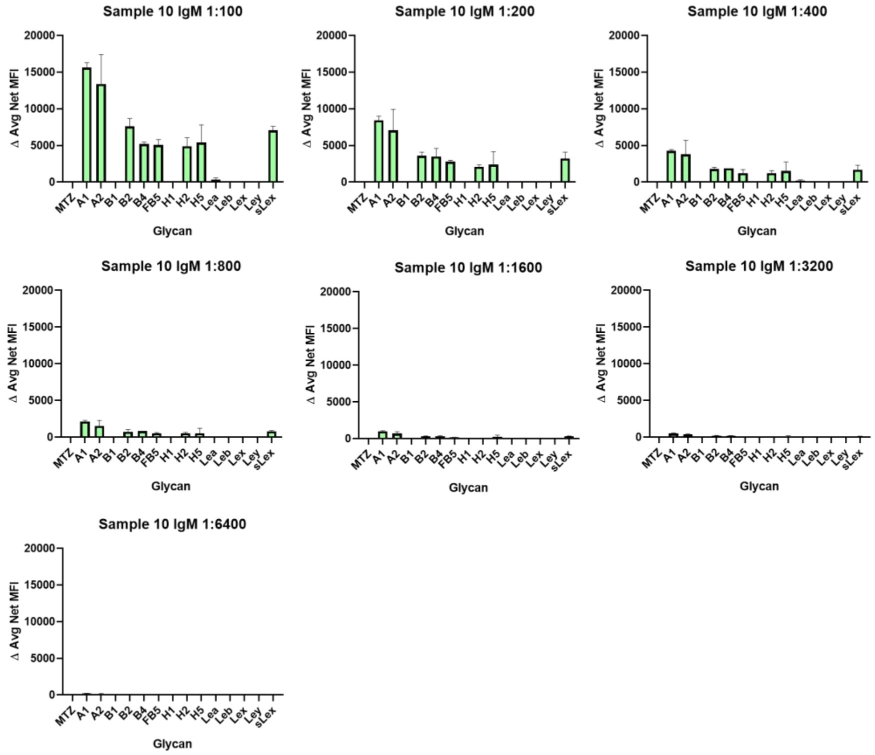

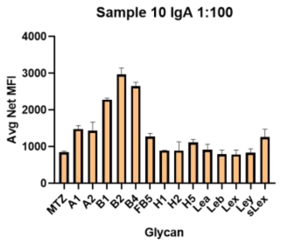

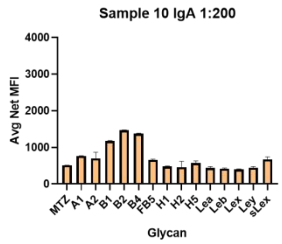

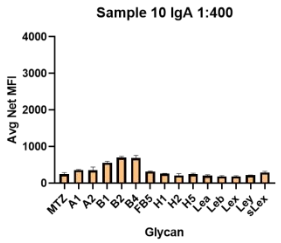

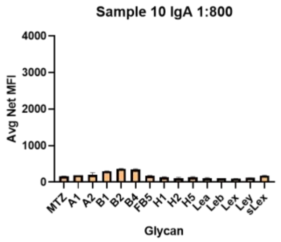

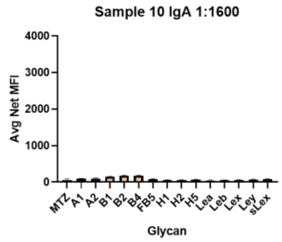

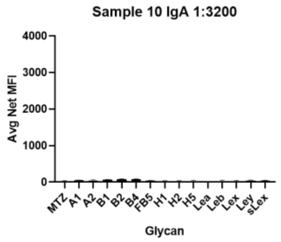

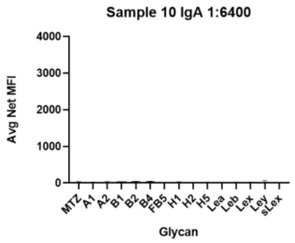

Sample 11 IgG, IgM, IgA

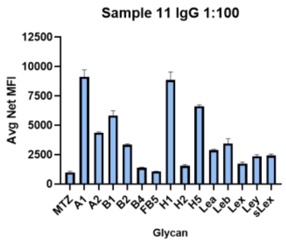

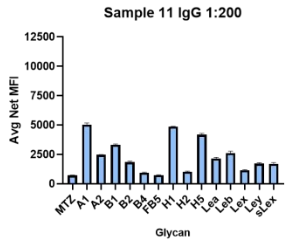

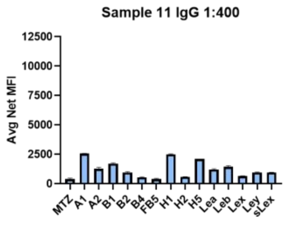

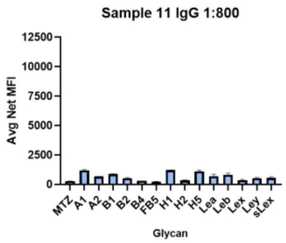

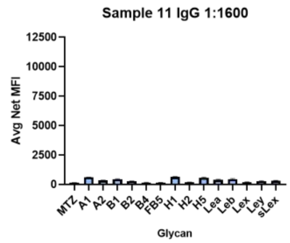

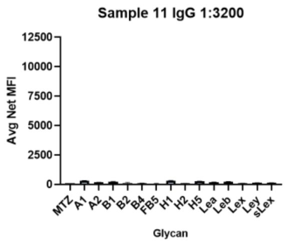

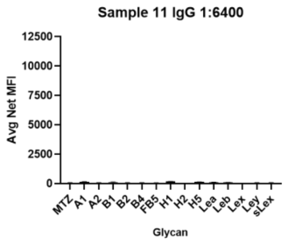

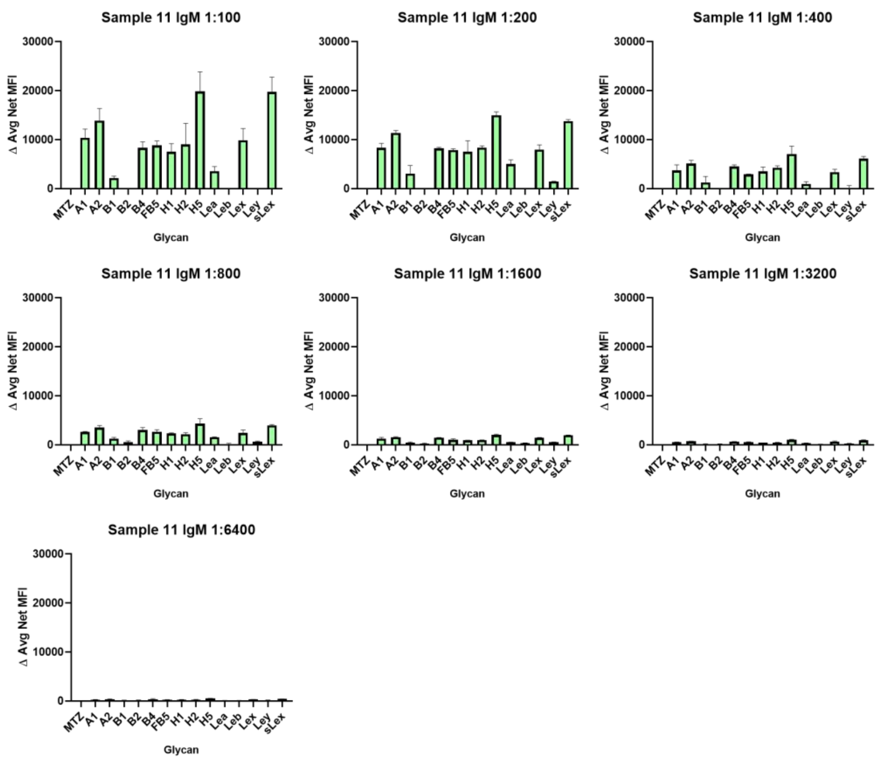
Sample 11 IgM 1:100
Sample 11 IgM 1:200
Sample 11 IgM 1:400
Sample 11 IgM 1:800
Sample 11 IgM 1:1600
Sample 11 IgM 1:3200
Sample 11 IgM 1:6400
Δ Avg Net MFI
0
10000
20000
30000
MTZ A1 A2 B1 B2 B4 FB5 H1 H2 H5 Lea Leb Lex Ley sLex
Glycan

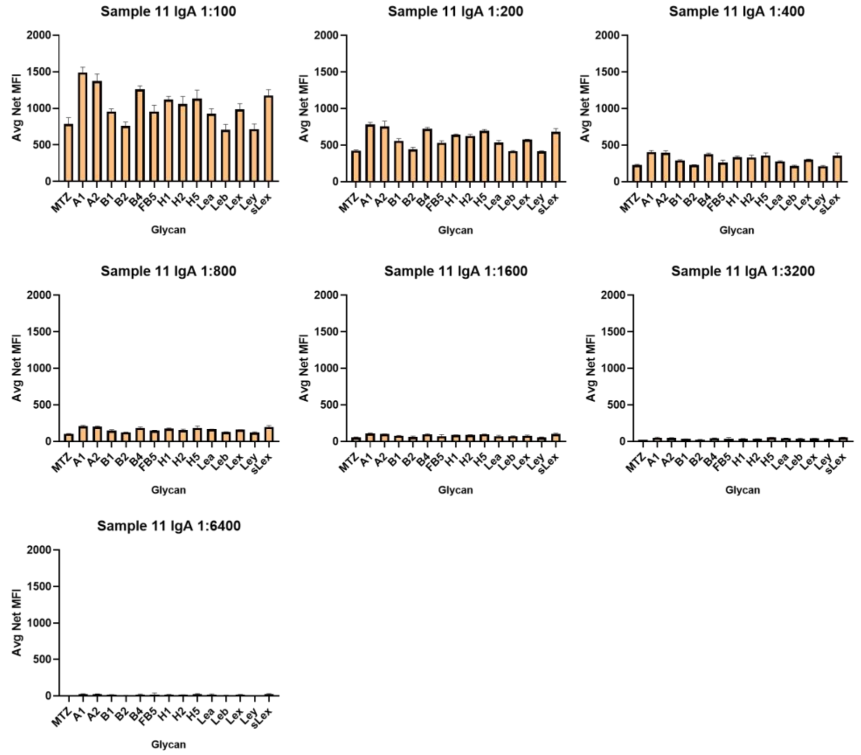
Sample 11 IgA 1:100
Sample 11 IgA 1:200
Sample 11 IgA 1:400
Sample 11 IgA 1:800
Sample 11 IgA 1:1600
Sample 11 IgA 1:3200
Sample 11 IgA 1:6400
Avg Net MFI
0
500
1000
1500
2000
MTZ A1 A2 B1 B2 B4 FB5 H1 H2 H5 Lea Leb Lex Ley sLex
Glycan

Sample 12 IgG, IgM, IgA

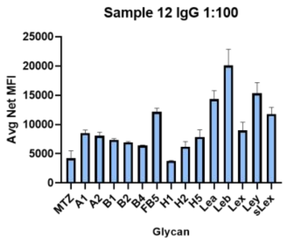

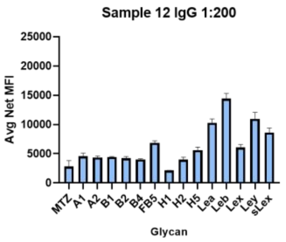

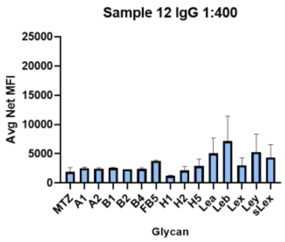

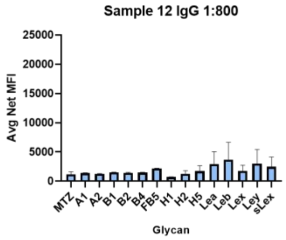

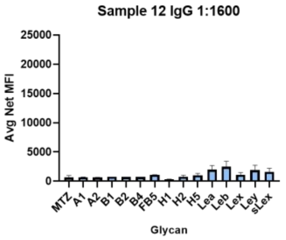

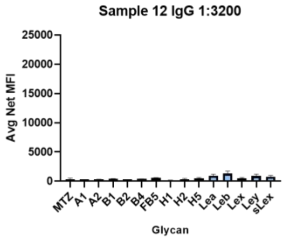

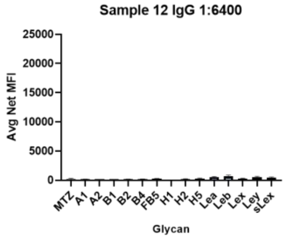

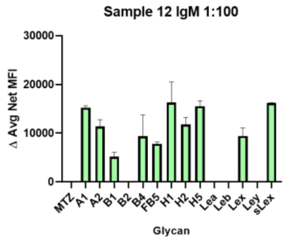

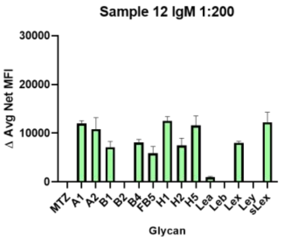

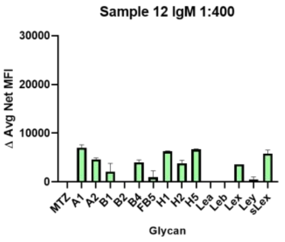

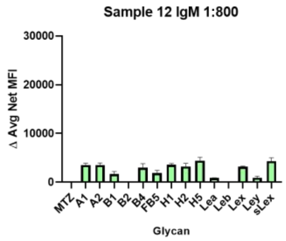

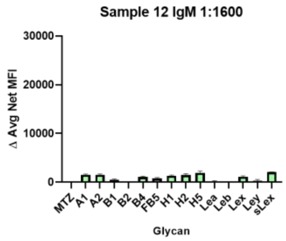

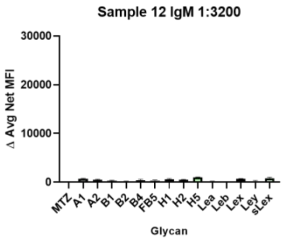

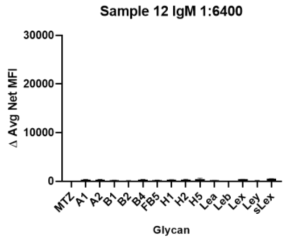

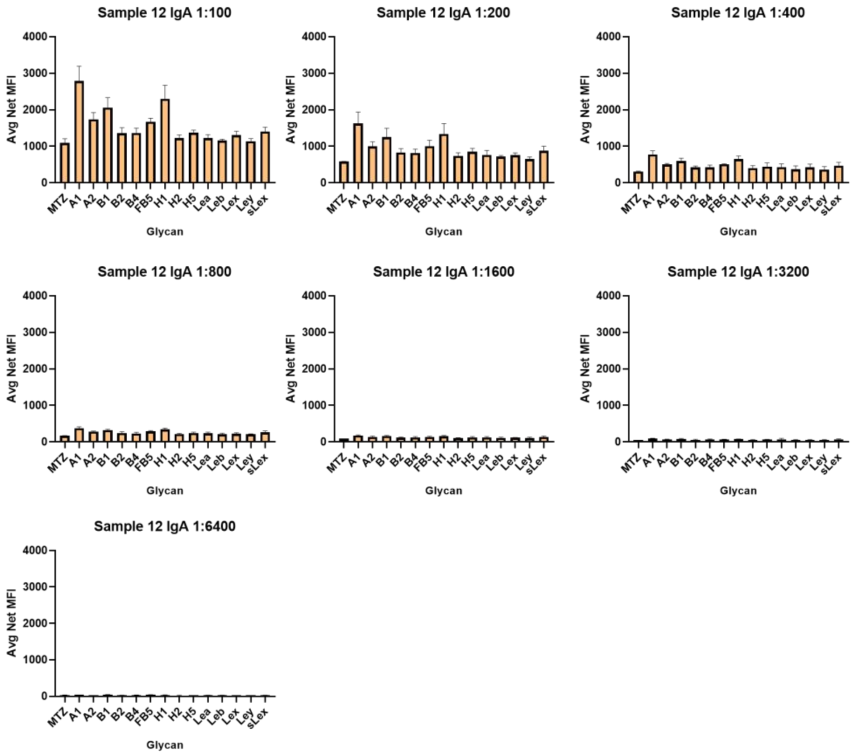

Sample 13 IgG, IgM, IgA

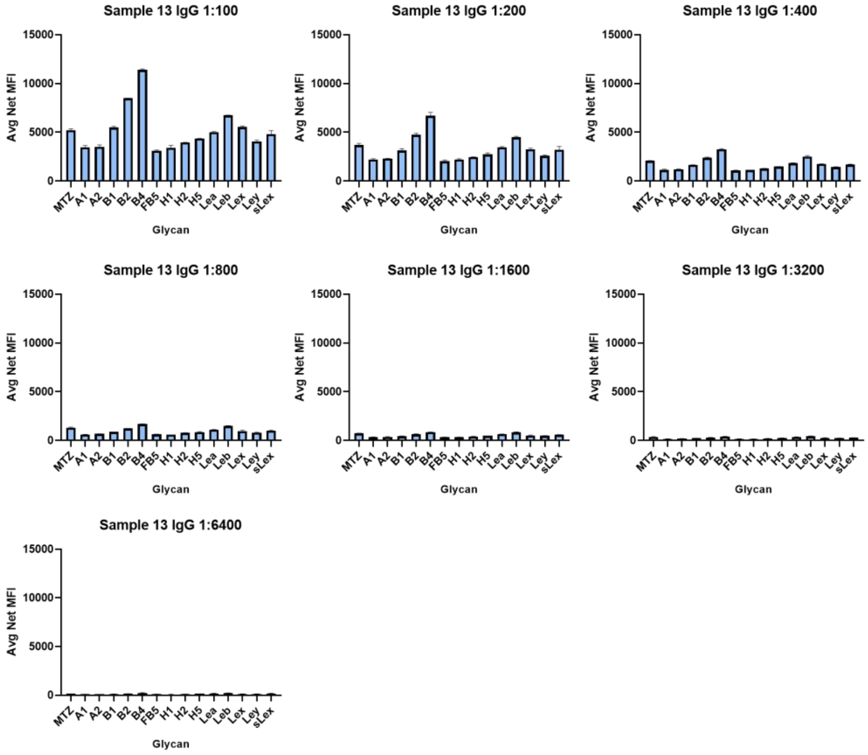

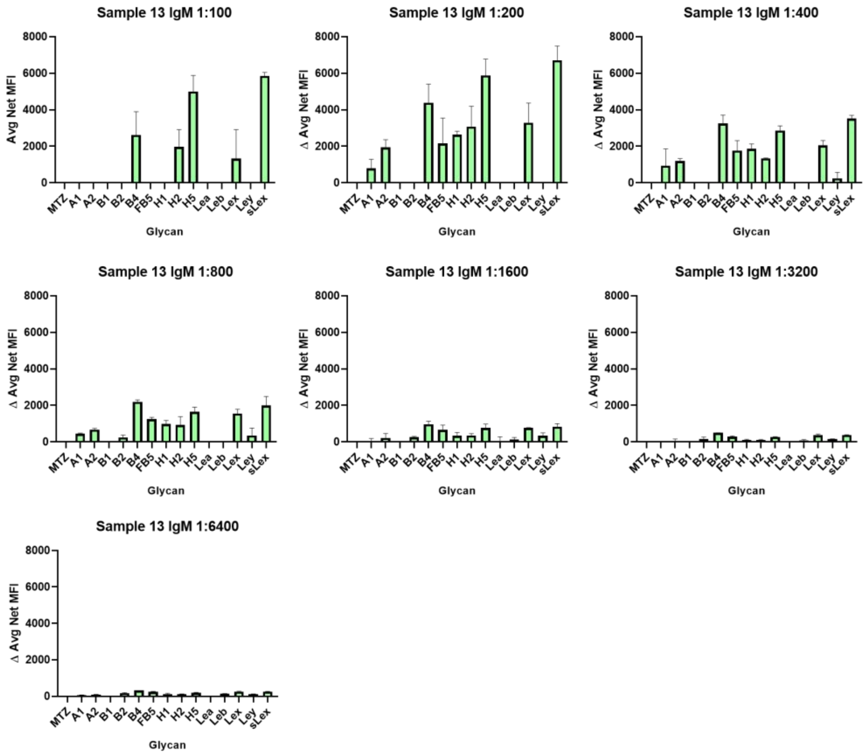
Sample 13 IgM 1:100
Avg Net MFI
Glycan
Sample 13 IgM 1:200
Δ Avg Net MFI
Glycan
Sample 13 IgM 1:400
Δ Avg Net MFI
Glycan
Sample 13 IgM 1:800
Δ Avg Net MFI
Glycan
Sample 13 IgM 1:1600
Δ Avg Net MFI
Glycan
Sample 13 IgM 1:3200
Δ Avg Net MFI
Glycan
Sample 13 IgM 1:6400
Δ Avg Net MFI
Glycan
0
2000
4000
6000
8000
MTZ
A1
A2
B1
B2
B4
FB5
H1
H2
H5
Lea
Leb
Lex
Ley
sLex

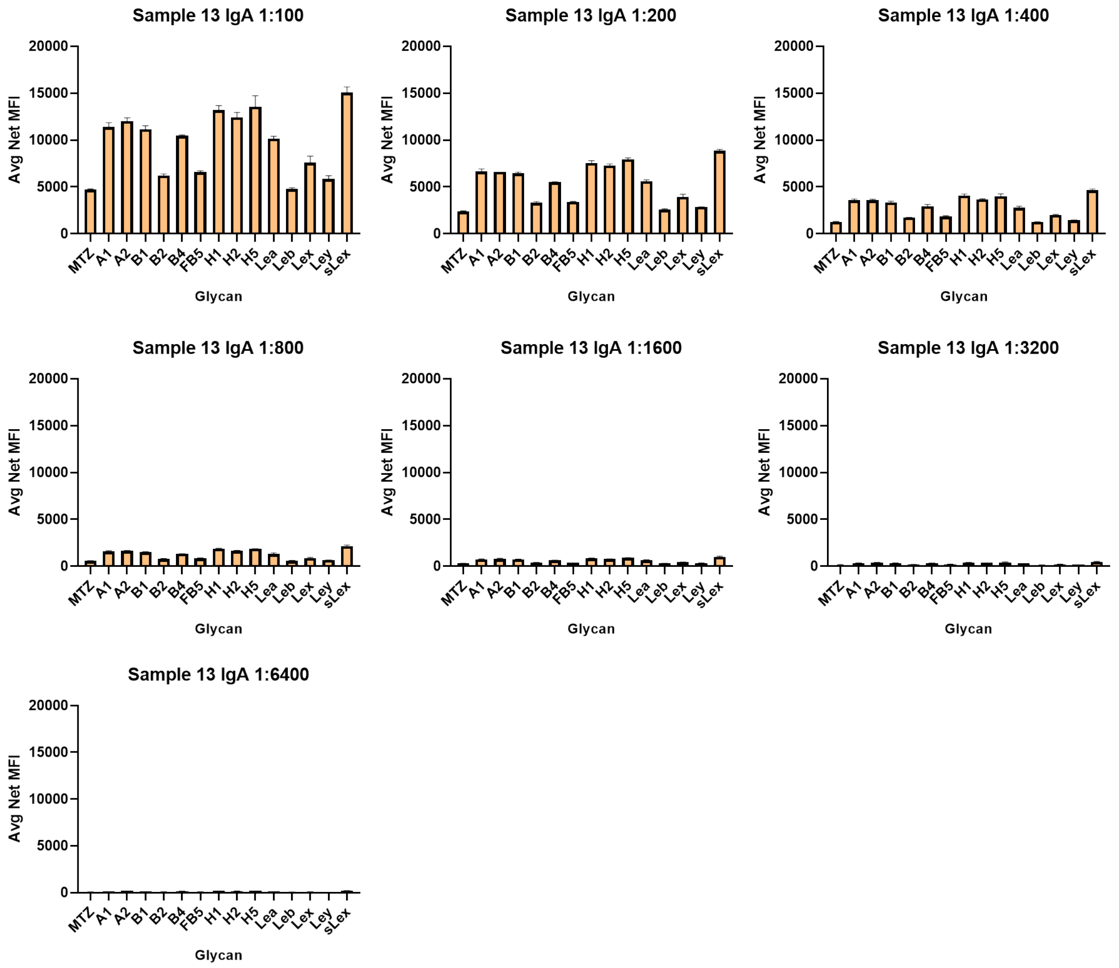

Figure S9. Profiling of anti-glycan IgG, IgM, and IgA in a series of 7 dilutions of 13 human fluid samples. The y-axis represents the average net (background subtracted) median fluorescence intensity (MFI) in anti-glycan IgG and IgA antibody. The y-axis represents the average net MFI subtracted MTZ (no glycan control) in anti-glycan IgM antibody. The data are presented as an average net MFI of two replicates; each experiment was repeated two times. Glycan names on x-axis correspond to **Figure 1C**. Error bars = +/- 1 SD.

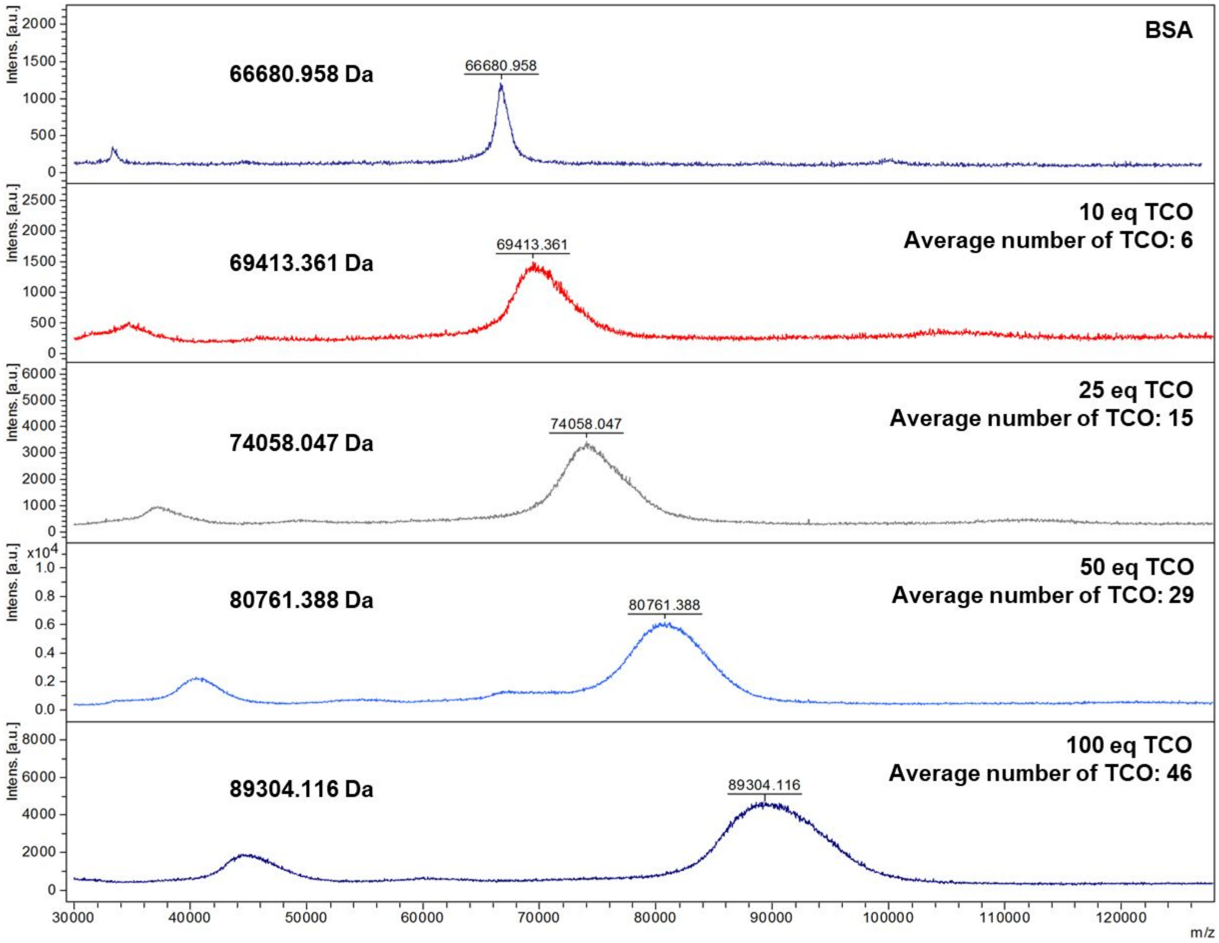

Figure S10. MALDI-TOF Mass spectrometry of BSA-PEG6-TCO.

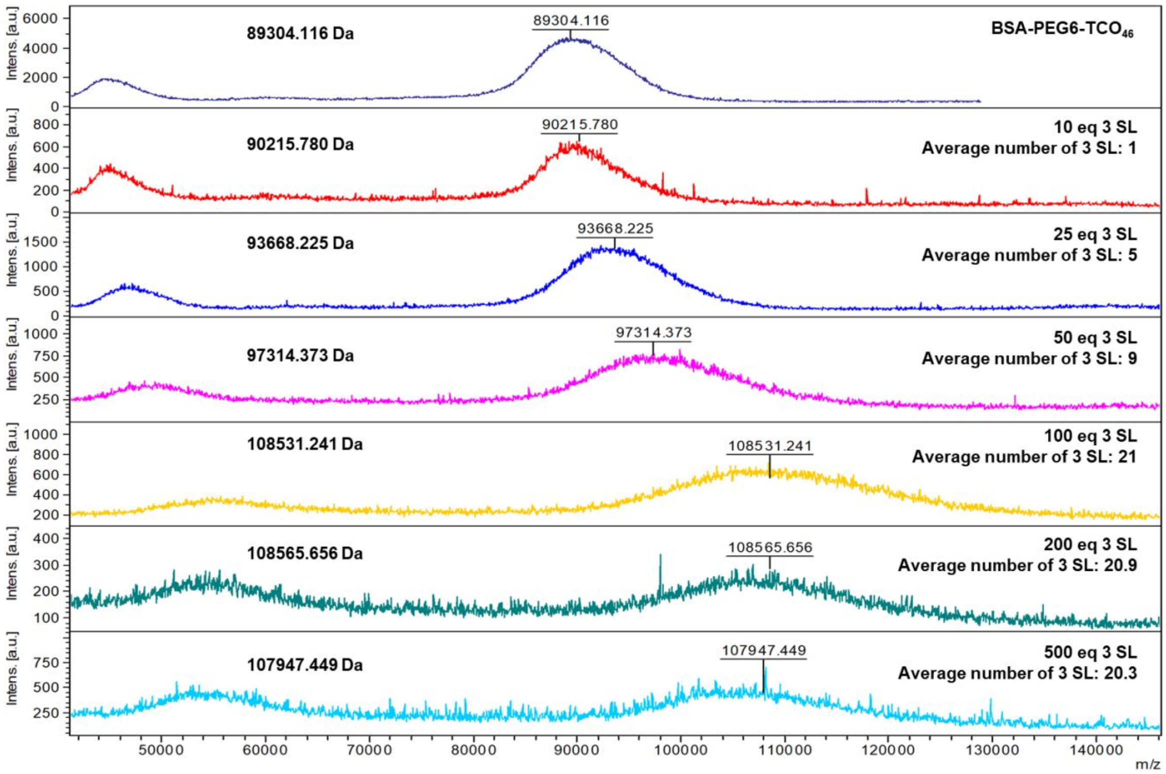

Figure S11. MALDI-TOF Mass spectrometry of BSA-3'-SL.

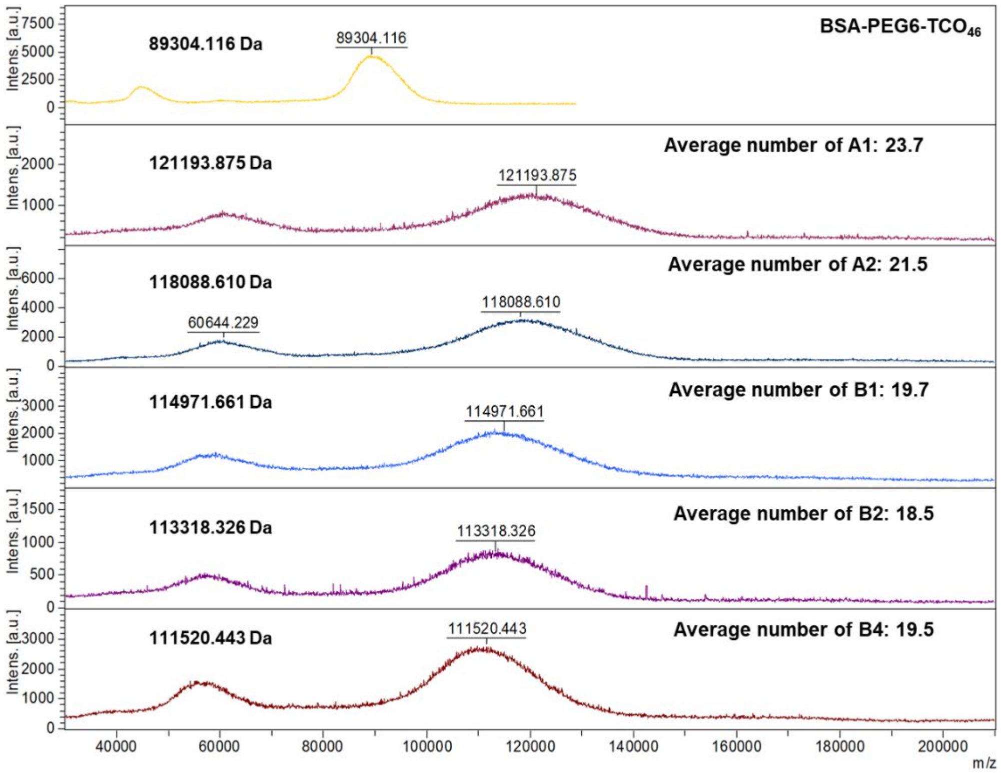
BSA-PEG6-TCO$_{46}$
89304.116 Da
89304.116
Average number of A1: 23.7
121193.875 Da
121193.875
Average number of A2: 21.5
118088.610 Da
60644.229
118088.610
Average number of B1: 19.7
114971.661 Da
114971.661
Average number of B2: 18.5
113318.326 Da
113318.326
Average number of B4: 19.5
111520.443 Da
111520.443
Intens. [a.u.]
m/z

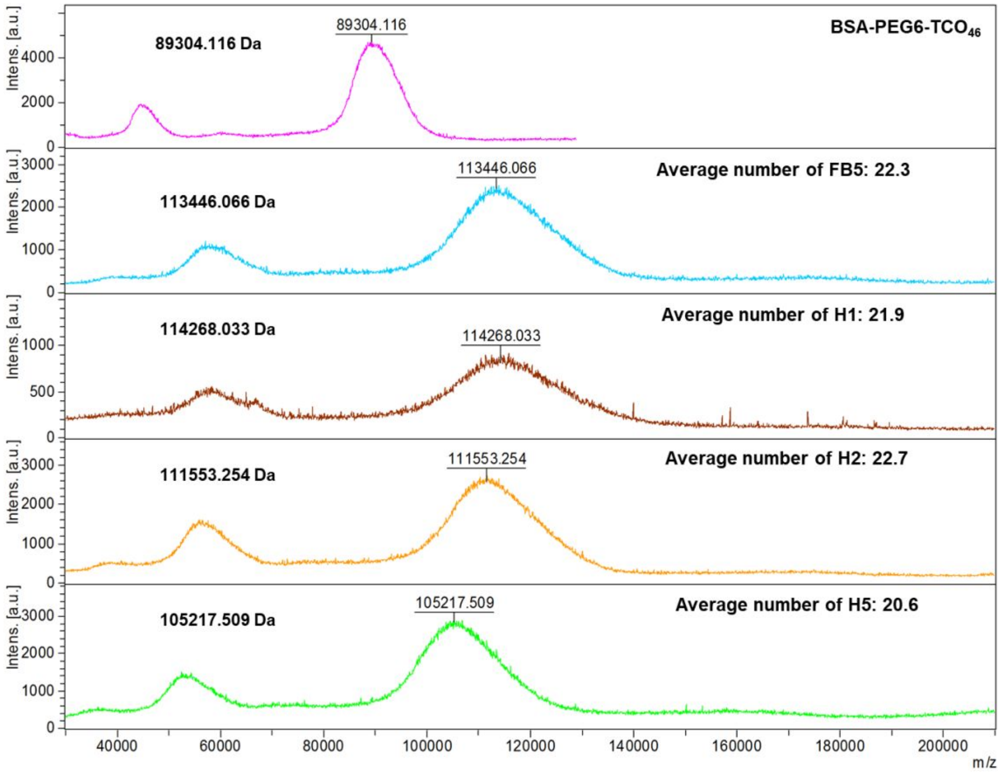
BSA-PEG6-TCO$_{46}$
89304.116 Da
89304.116
Average number of FB5: 22.3
113446.066 Da
113446.066
Average number of H1: 21.9
114268.033 Da
114268.033
Average number of H2: 22.7
111553.254 Da
111553.254
Average number of H5: 20.6
105217.509 Da
105217.509
Intens. [a.u.]
m/z

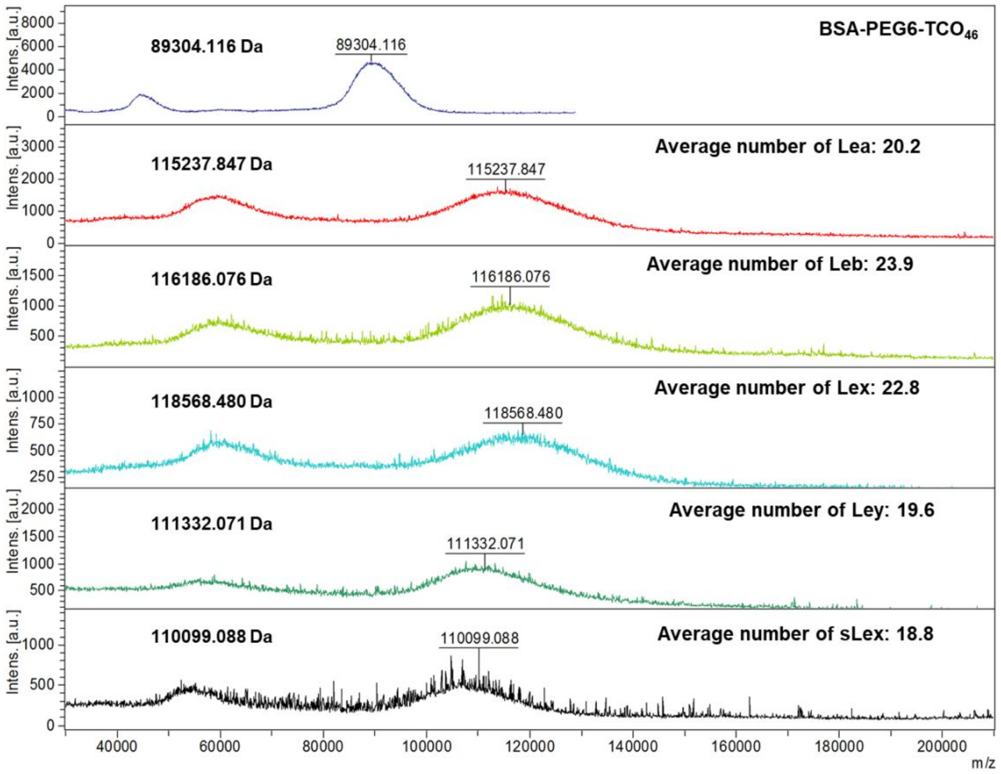

Figure S12. MALDI-TOF Mass spectrometry of neoglycoprotein library.

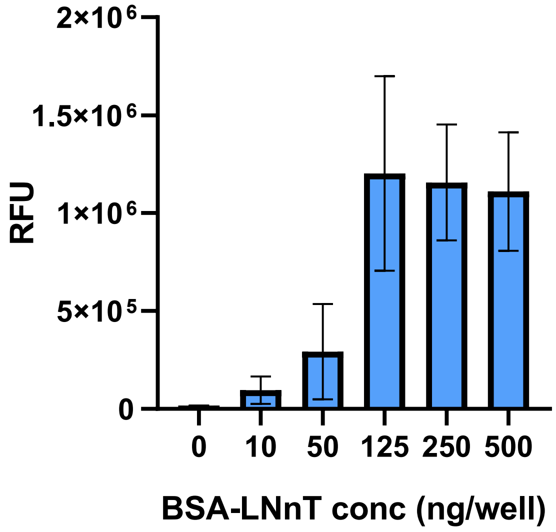

Figure S13. The optimization condition of immobilization of neoglycoprotein BSA-LNnT in 96-well plate. The data are presented as an average RFU of two replicates; each experiment was repeated two times. Error bars = +/- 1 SD. RFU = relative fluorescence units.

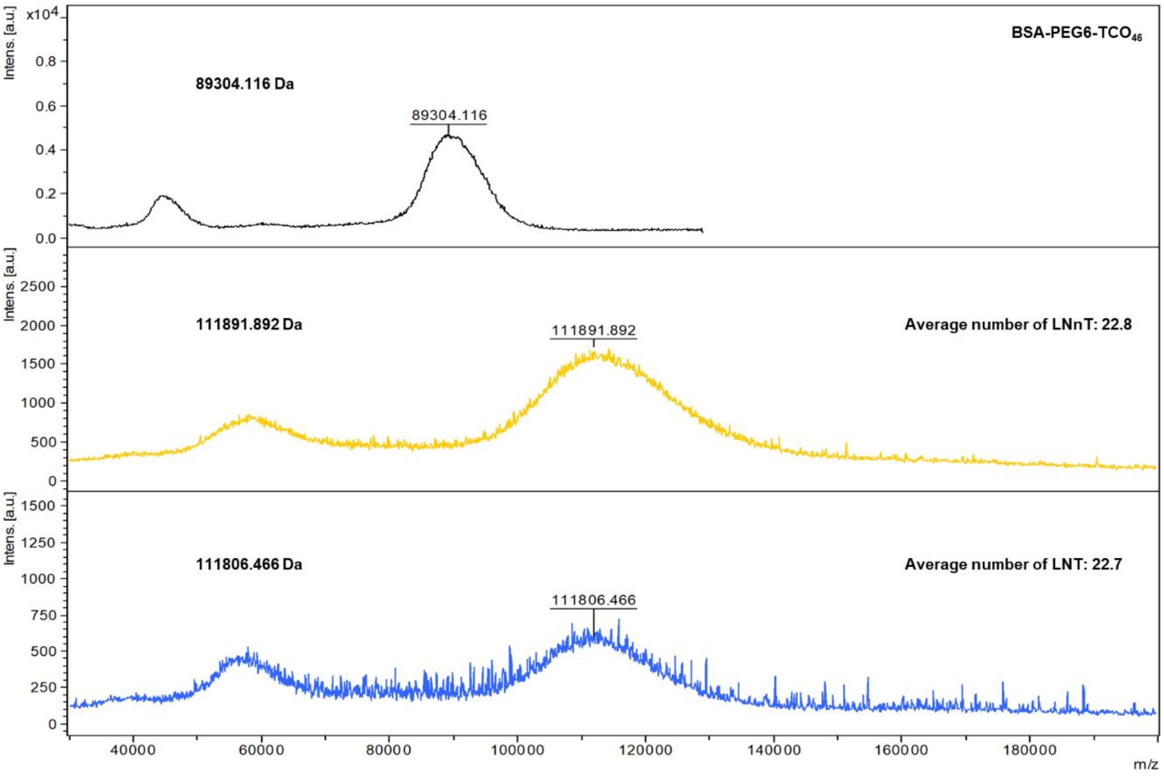

Figure S14. MALDI-TOF Mass spectrometry of BSA-LNnT and BSA-LNT.

Figure S15. NMR Spectra

Compound 2 ^{1}H NMR $CDCl_3$, 400 MHz

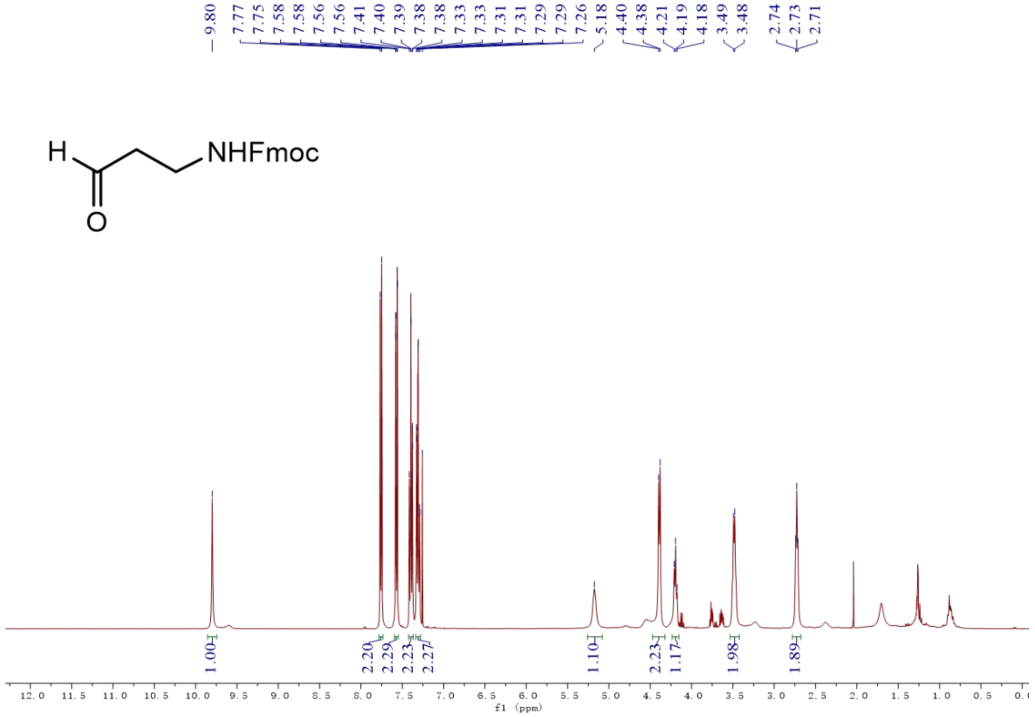

Compound 2 ^{13}C NMR $CDCl_3$, 100 MHz

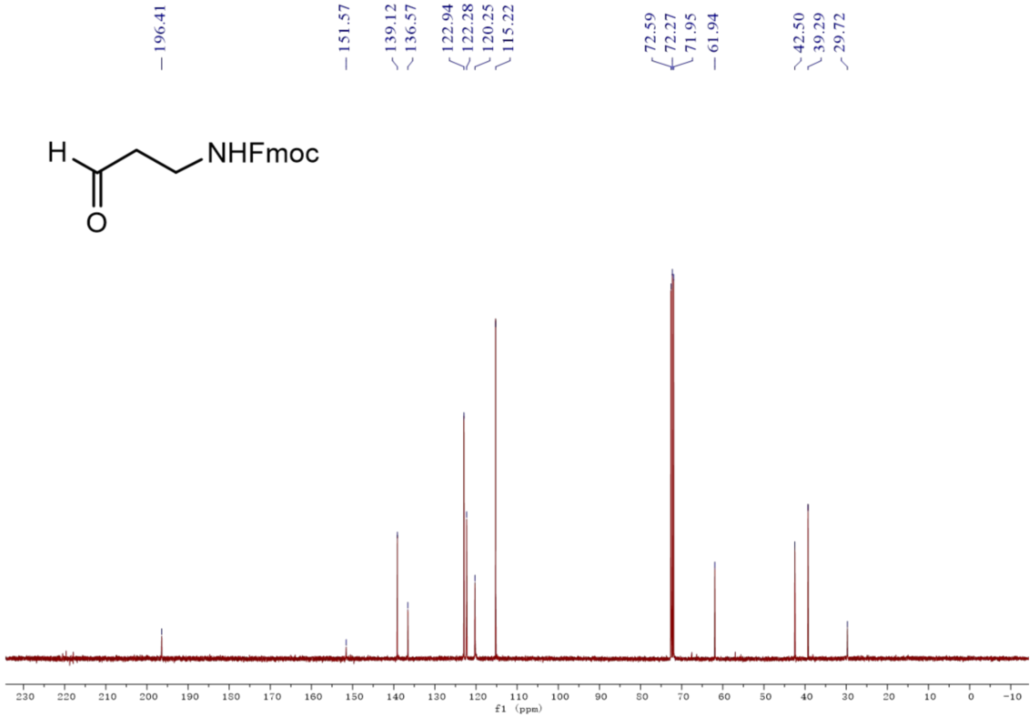

Compound 3 ^{1}H NMR $CDCl_3$, 400 MHz

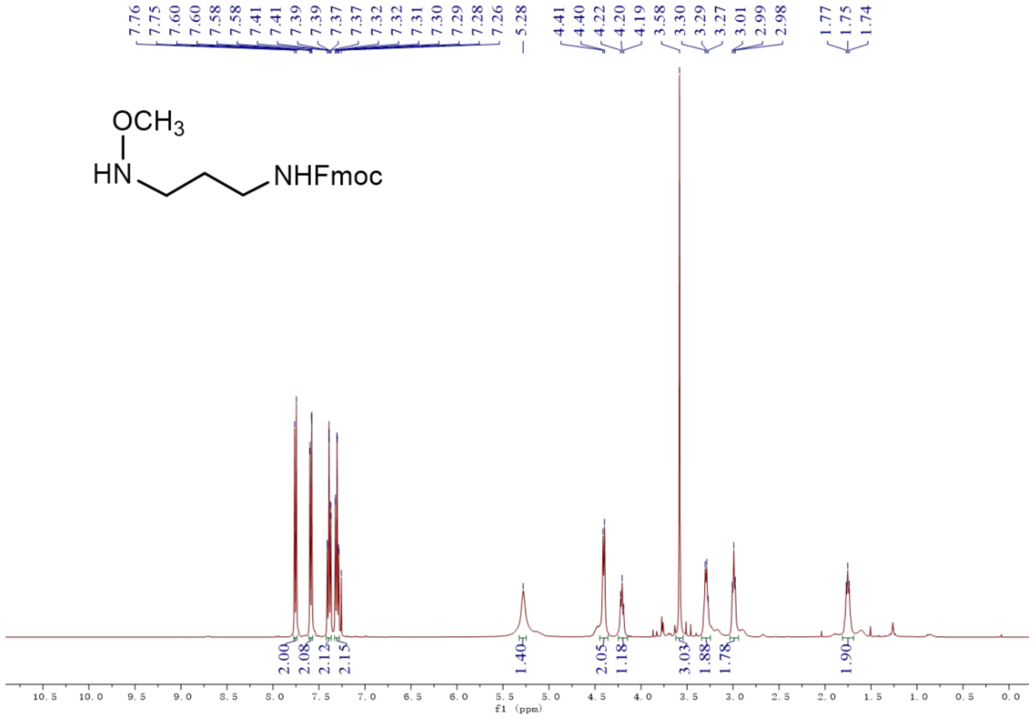

Compound 3 ^{13}C NMR $CDCl_3$, 100 MHz

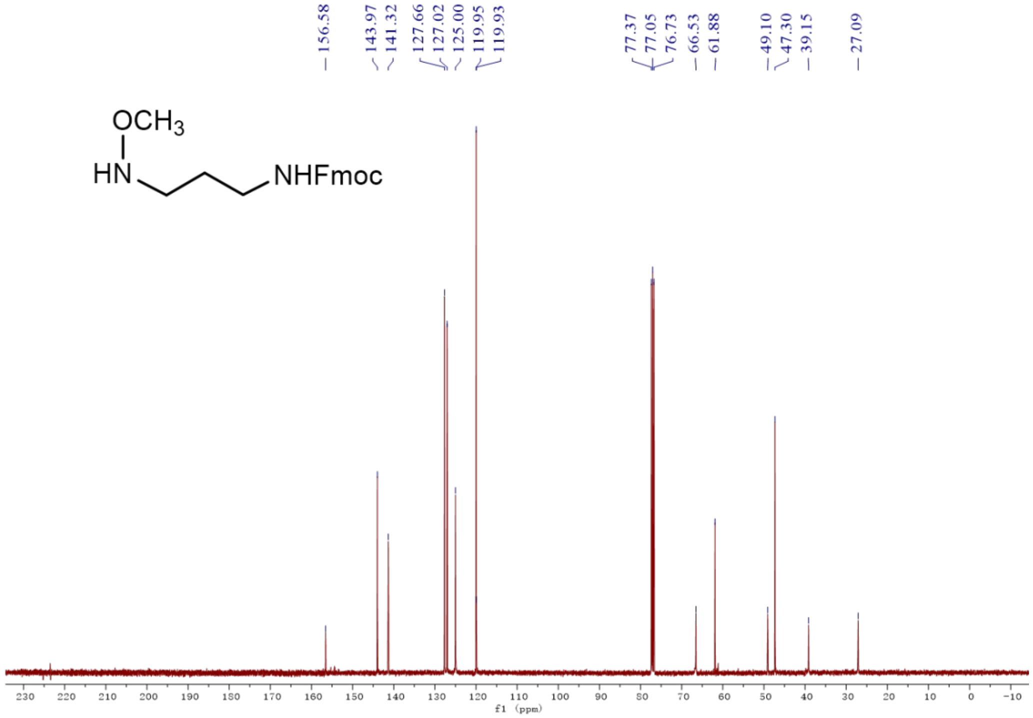

Compound 4 ^{1}H NMR $CDCl_3$, 400 MHz

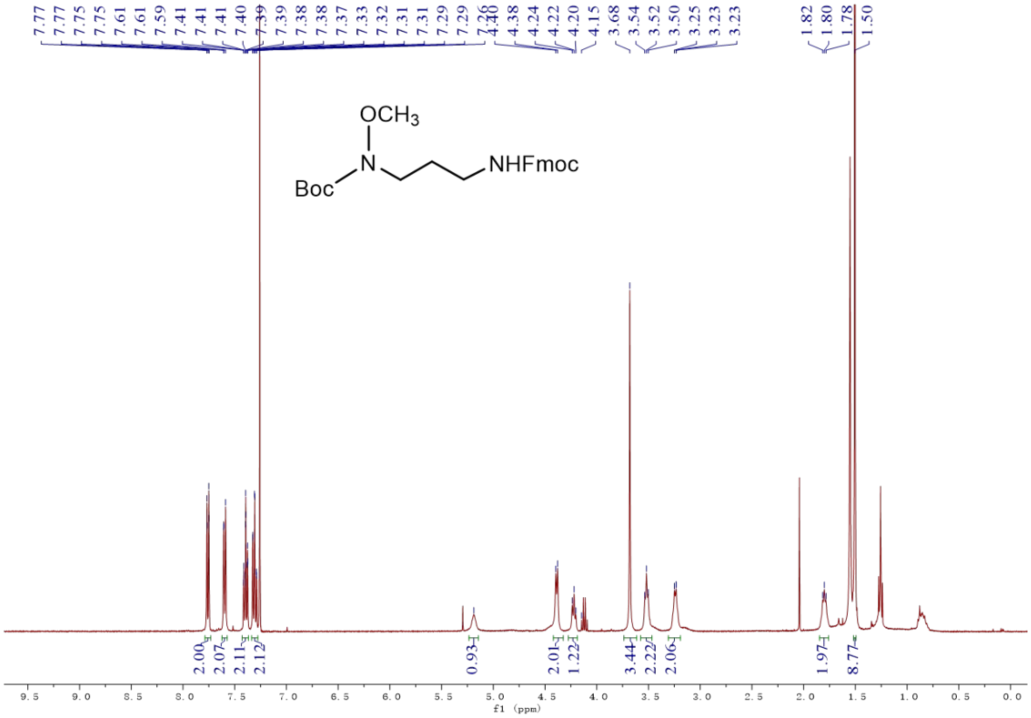

Compound 4 ^{13}C NMR $CDCl_3$, 100 MHz

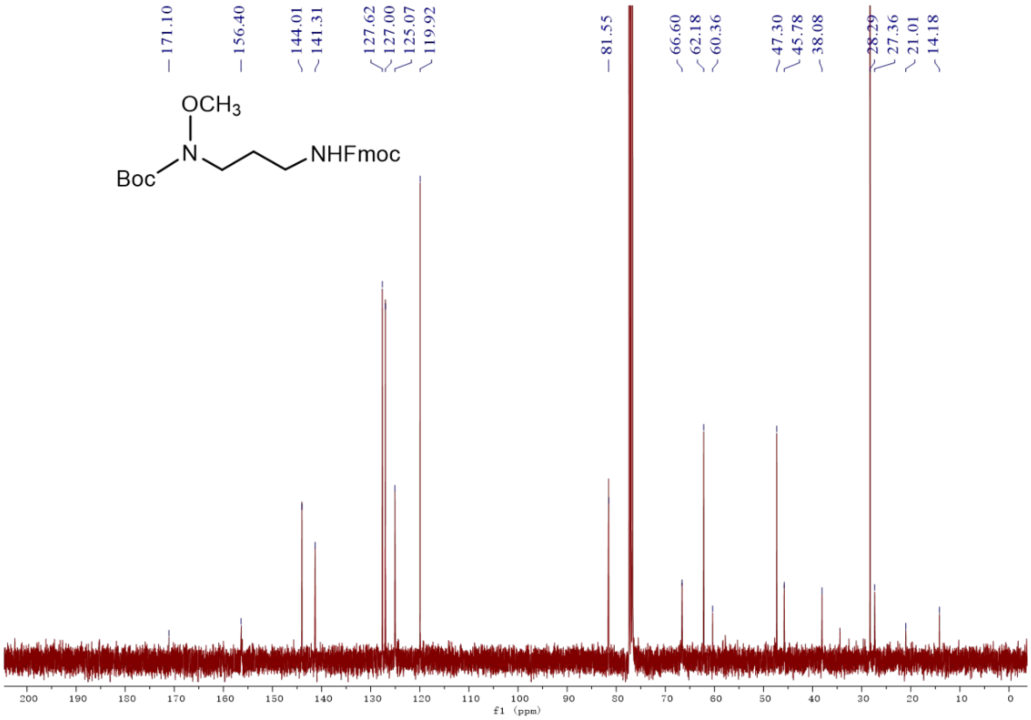

Compound 5 ^{1}H NMR $CDCl_3$, 400 MHz

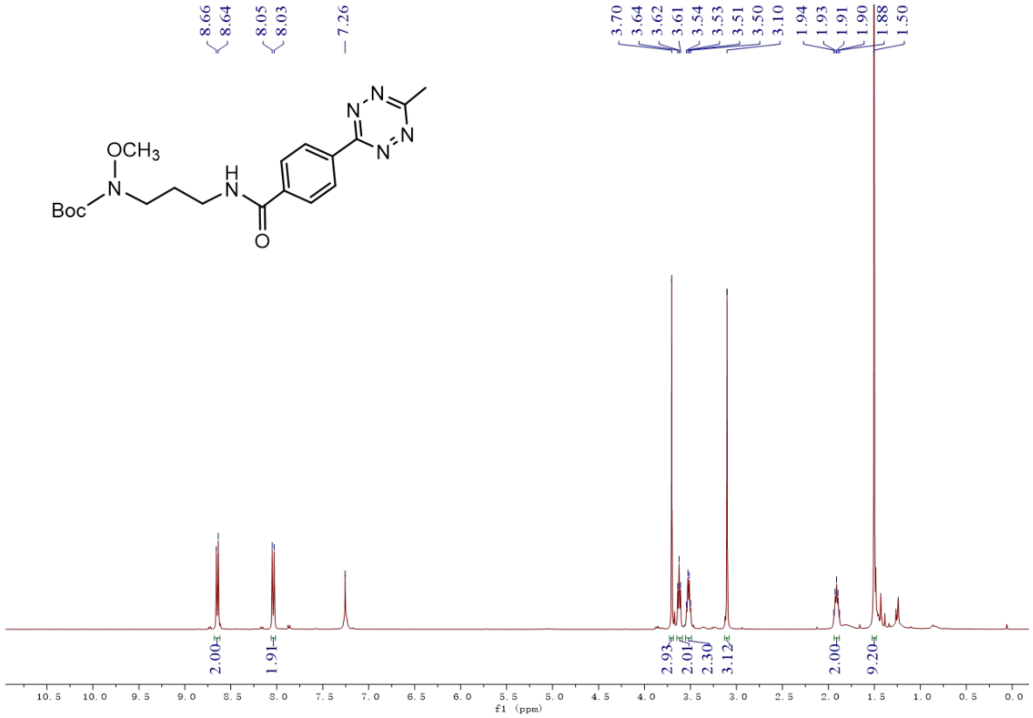

Compound 5 ^{13}C NMR $CDCl_3$, 100 MHz

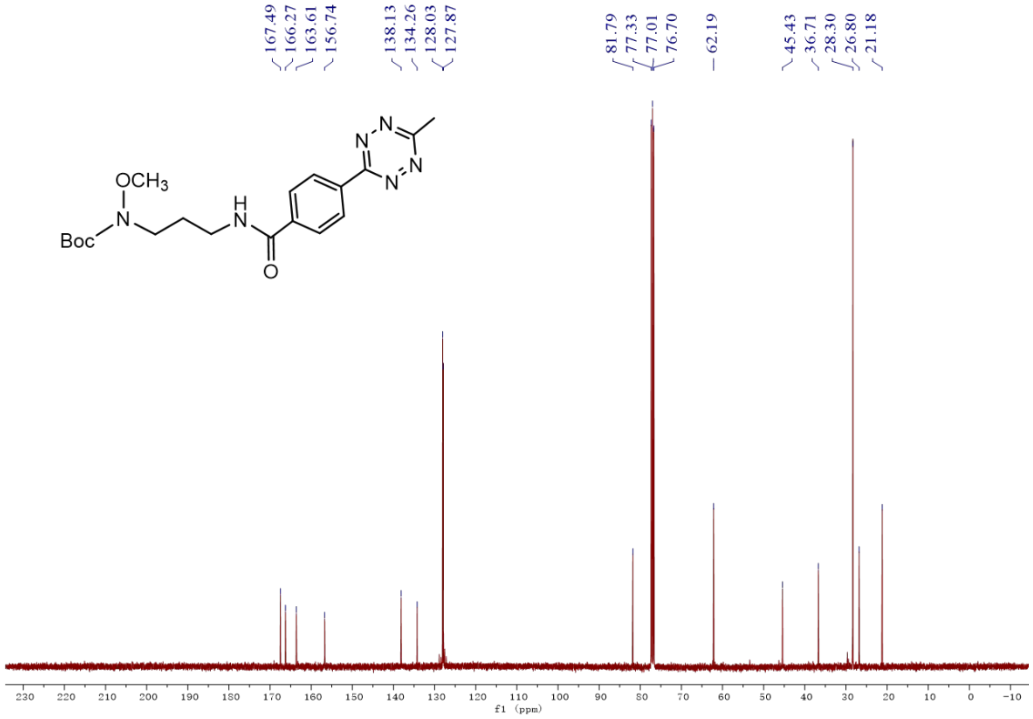

MTZ HCl salt ^{1}H NMR D_2O, 400 MHz

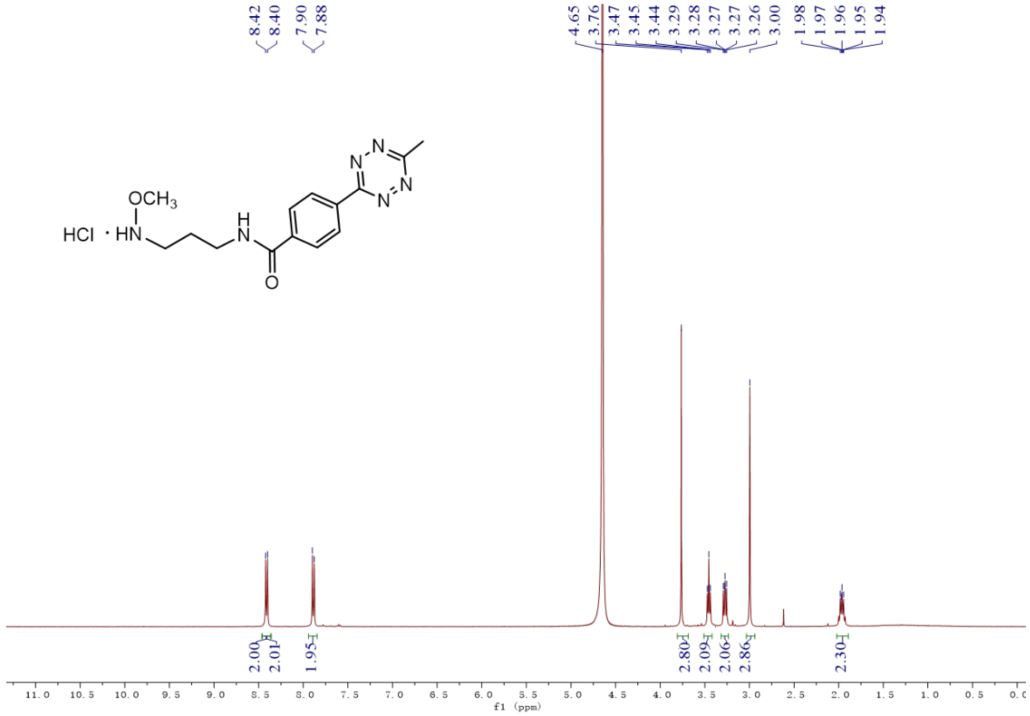

MTZ HCl salt ^{13}C NMR D_2O, 100 MHz

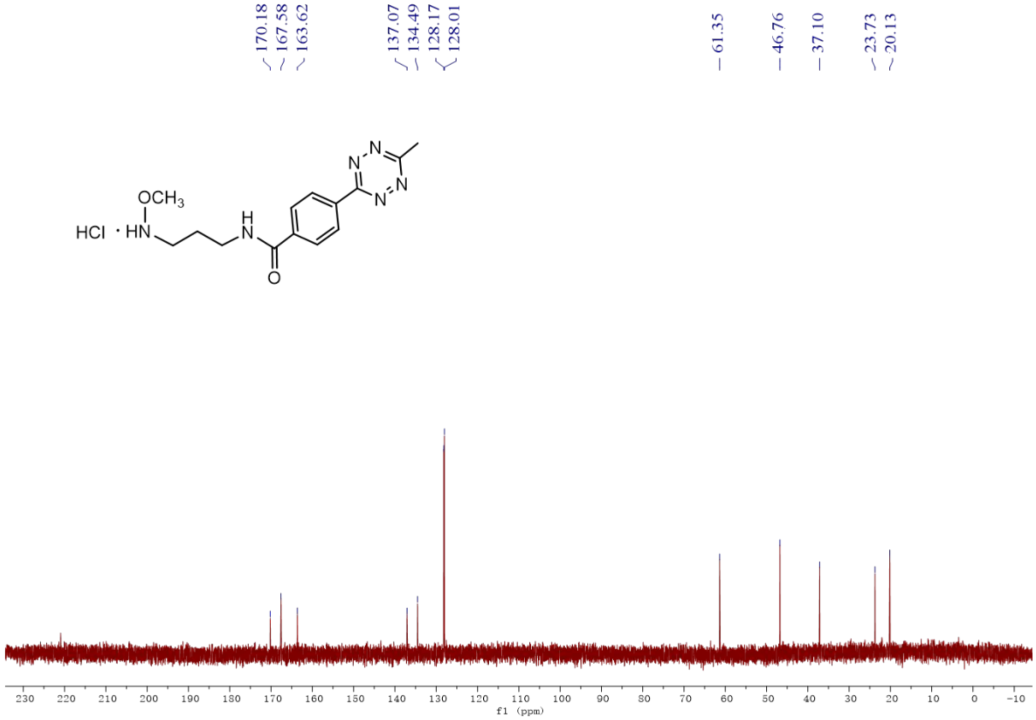

Supplement: Supplementary file 1 [file oc4c02124_si_001.pdf]
